# Social diffusion sources can escape detection

**DOI:** 10.1016/j.isci.2022.104956

**Published:** 2022-08-19

**Authors:** Marcin Waniek, Petter Holme, Manuel Cebrian, Talal Rahwan

**Affiliations:** 1 Computer Science, Division of Science, New York University Abu Dhabi, Saadiyat Island, Abu Dhabi 129188, UAE; 2 Department of Computer Science, Aalto University, Otakaari 1B, 02150 Espoo, Finland; 3 Center for Computational Social Science, Kobe University, 2-1 Rokkodai-cho, Nada-ku, Kobe 657-8501, Japan; 4 Center for Humans and Machines, Max Planck Institute for Human Development, Lentzeallee 94, 14195 Berlin, Germany; 5 Department of Statistics, Universidad Carlos III de Madrid, Calle Madrid 126, 28903 Getafe, Spain; 6 UC3M-Santander Big Data Institute, Calle Madrid 135, 28903 Getafe, Spain

**Keywords:** Applied sciences, Computer science, Internet-based information systems, Computer systems organization, Internet, Social sciences

## Abstract

Influencing others through social networks is fundamental to all human societies. Whether this happens through the diffusion of rumors, opinions, or viruses, identifying the diffusion source (i.e., the person that initiated it) is a problem that has attracted much research interest. Nevertheless, existing literature has ignored the possibility that the source might strategically modify the network structure (by rewiring links or introducing fake nodes) to escape detection. Here, without restricting our analysis to any particular diffusion scenario, we close this gap by evaluating two mechanisms that hide the source—one stemming from the source’s actions, the other from the network structure itself. This reveals that sources can easily escape detection, and that removing links is far more effective than introducing fake nodes. Thus, efforts should focus on exposing concealed ties rather than planted entities; such exposure would drastically improve our chances of detecting the diffusion source.

## Highlights

• We study the problem of hiding the diffusion source from source detection algorithms
• Finding an optimal way of hiding the source is usually computationally intractable
• In many cases, the source is well hidden without any strategic modifications
• If the source is exposed, simple heuristic solutions allow it to avoid detection

## Graphical abstract

**Figure:**
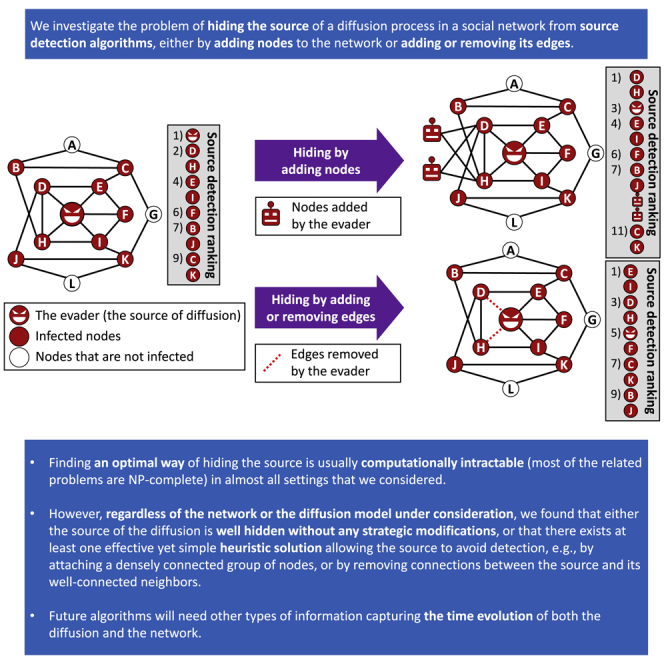

## Introduction

As humans, we are perpetually involved in, and affected by, things spreading in networks—from infections (Block et al., 2020; Chiu et al., 2020) to ideas, from financial distress to fake news (Barrat et al., 2008; Lehmann and Ahn, 2018). Furthermore, we live in an increasingly networked world—the networks of our society are becoming denser, meaning that spreading phenomena happen with accelerating speed (Barabási, 2016). Occasionally, we do not know who started the spreading. However, recent research has shown that we can often infer the source with great accuracy (Lokhov et al., 2013; Jain et al., 2016; Shah et al., 2020). If the spreading has an illicit intent or negative consequences for the source—like bioterrorism, disinformation, or whistleblowing in an authoritarian society—the source would want to hide from such source detection algorithms. In this article, we study the conditions under which such hiding can be successful. To keep the results as widely applicable as possible, and to conform to the praxis of Network Science (Lokhov et al., 2013; Jain et al., 2016; Shah et al., 2020; De Domenico et al., 2013; Was et al., 2020; Waniek et al., 2018), we avoid making domain-specific assumptions about the nature of the social diffusion. Thus, our models, simulations, and theoretical analyses are in principle applicable to a diverse set of scenarios. The diffusion process under consideration could represent a piece of fake news spreading on social media, a vicious rumor circulating via word of mouth, or a virus infecting a computer network. We will also occasionally borrow the language from infectious disease epidemiology. More speculatively, our analysis could also apply to disease spreading, but that would rely on uncertain assumptions about the available mechanisms of both evasion and tracing.

The main challenge to understand the possible obfuscation of the diffusion source is that it is a problem with two components. First, networks have an innate ability to hide the source; although most research in the literature has focused on designing source detection algorithms, the efficiency of these algorithms strongly depends on the network structure. Second, by changing its local network surrounding, the source can hide its identity. So far, no theory has been able to separate these two factors, and understand how they are affected by the dynamics of the spreading and the timing of the source detection. This article builds such a theory from the systematic simulations, with and without active obfuscation of the source. For the sake of parsimony, we base our study on simple contagion (Lehmann and Ahn, 2018; Goffman and Newill, 1964), while varying the network structure, source detection, and timing of these.

We begin our analysis by investigating the theoretical limits of the problem of hiding the source of diffusion. We mathematically prove that identifying an optimal solution to this problem is practically impossible. Based on this, we turn our attention to feasible—albeit not optimal—ways in which adversaries may hide the source. The first way involves executing several heuristics that either introduce new nodes or rewire (i.e., add or remove) existing links. The second way relies on the network structure itself, without any intervention from the source. We evaluate both ways by running simulations in real-life networks, as well as synthetic networks with varying structure and density. This evaluation is done via exact methods when the network is of moderate size and approximation when the network is massive. Although most of our analysis focuses on a simple contagion model, we also perform simulations with alternative diffusion models, including variants of complex contagion. We also study how the source’s actions affect other nodes part of the same cascade and how the source detection algorithms are influenced, given imperfect knowledge about the network structure. We conduct several sensitivity analyses, varying how the source is selected, the parameters for generating synthetic networks, and the approximations by which we evaluate the heuristics. Finally, we validate our findings using a large-scale dataset of new hashtags introduced to Twitter, where both the structure of the network and the information cascades are real.

The main contributions of our work can be summarized as follows. We present the first systematic analysis of hiding the source of social diffusion. We formally prove the computational intractability of the decision problems faced by the source who wishes to conceal their identity, thus effectively eliminating the possibility of using optimal hiding strategies. At the same time, we explore a wide variety of settings and in each of them show that even if the source is not already obscured by the structure of the network itself, there exists heuristic solutions that can hide the source from the prying eyes of source detection algorithms in an efficient, albeit not optimal way. Altogether, our analysis strongly suggests that the source of social diffusion can indeed escape detection.

## Results

### Problem overview and theoretical analysis

We consider the problem of hiding the source of a diffusion process in a network. In particular, we consider an undirected network G=(V,E), where one of the nodes, $v^{†}\in V$, starts a diffusion process, resulting in a subset of nodes I becoming infected. In this work, we assume that this process follows the Susceptible-Infected (SI) model (Kermack and McKendrick, 1927); see STAR Methods for a formal description of the network notation and the SI model. We consider situations where $v^{†}$ wishes to avoid being detected as the source of the diffusion process. Hence, we call the node $v^{†}$ *the evader*. We also assume the existence of another entity, called *the seeker*, whose goal is to identify the origin of the diffusion using source detection algorithms. In our analysis, we consider source detection algorithms that return a ranking of network nodes (Comin and Costa, 2011; Shah and Zaman, 2011; Jain et al., 2016; Antulov-Fantulin et al., 2015), where the node at the top position in the ranking is identified as the source; see STAR Methods for more details. The goal of the evader is then to introduce modifications to the network structure (after the diffusion has taken place) in order to avoid being identified by the seeker as the source. We consider the two (as we argue below) most realistic types of such modifications: (1) adding nodes and (2) modifying (i.e., adding or removing) edges.

Next, we provide more details about the two types of modifications, starting with the one in which nodes are added to the network. For instance, these can be individuals working for the evader, who deliberately position themselves in strategic locations within the social network to confuse the seeker. We refer to such individuals as “confederates” throughout the article (although this term suggests that they are willingly and knowingly helping the evader, this does not have to be the case). Then, the problem faced by the evader is to determine the contacts of each confederate. Thus, although the evader wishes to hide by adding nodes, the optimization problem faced by the evader is to choose which edges, not nodes, to add to the network. Note that this is a variation of the well-known Sybil attack (Douceur, 2002), where an entity affects a system by using multiple identities. The action of adding a new node to the network might be interpreted in various ways, depending on the setting under consideration. For example, if the diffusion process is an information cascade on a social media platform, the added node could be a bot account that follows certain existing accounts and reposts content shared by the evader. On the other hand, if the diffusion process is an infectious disease, the added node could be an infected person paid to falsely claim that they visited a certain location at a certain time. We also consider an alternative way in which the evader may conceal their true nature as the source of the diffusion. Instead of adding confederates to the network, the evader can modify (i.e., add or remove) the network edges after the diffusion has taken place. For instance, the evader who wishes to obfuscate the origins of a process spreading in the real world (such as an infectious disease) could claim to have met someone when in reality they have not, or vice versa, hoping that such modifications would mislead the source detection algorithms. On the other hand, if the spreading process represents a piece of information (such as fake news), the evader could deliberately stage a telephone conversation with someone (which would represent edge addition), or cease to follow someone on social media (which would represent edge removal). When it comes to the capabilities of the evader, in the general version of the problem it could be assumed that the evader is able to add or remove any edge in the network. However, in our simulations, we analyze a more realistic setting, in which the evader can only modify edges in their direct network vicinity. To be precise, we assume that the evader can only remove edges between themselves and their neighbors, and can only add edges between themselves and the neighbors of their neighbors.

**Figure 1:**
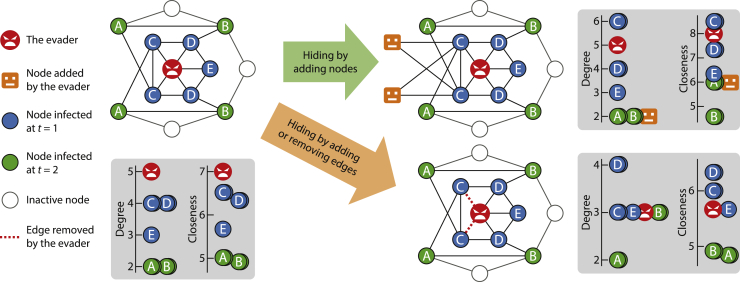
An overview of the hiding process The network on the left represents the original structure before the hiding process. Here, the red node is the evader, and the blue nodes are those infected in the first round of diffusion, i.e., at time step t=1, while the green nodes are those infected in t=2. The gray frame next to this network depicts the rankings computed by two source detection algorithms, namely Degree and Closeness (nodes that have the same ranking were given the same label, e.g., see the two nodes labeled C). The numbers on the scale represent the score assigned by each algorithm, implying that the node positioned at the top is the most likely source according to that algorithm. The network in the top-right corner represents a possible scenario where the evader tries to hide by adding two confederates, and connecting them to the nodes labeled C. The network in the bottom-right corner represents another scenario where the evader tries to hide by removing the dotted edges. The gray frames next to these networks show how the rankings change as a result of these modifications.

Figure 1 illustrates an example of the hiding process. The network on the left represents the original structure, i.e., the one in which the diffusion takes place. Here, we focus on two source detection algorithms, namely Degree (which counts the number of infected neighbors that one has) and Closeness (which measures one’s distance to other infected nodes); see STAR Methods for formal definitions of these algorithms. According to both algorithms, the evader (represented as the red node) is identified as the source of diffusion (see how the evader occupies the top position in the ranking produced by each algorithm). The networks on the right illustrate two possible scenarios in which the evader hides its identity. The evader could avoid detection by introducing newly infected nodes, as exemplified in the top-right network. After adding the two confederates, the evader connects them to the nodes labeled C (e.g., asks them to claim they were in contact with the nodes labeled C). Consequently, the evader drops to the third position in the rankings produced by both the Degree and Closeness algorithms. Alternatively, the evader could try to avoid detection by removing the edges between themselves and the two nodes labeled C as exemplified in the bottom-right network (a possible interpretation of this action is that the evader denies having met these two individuals). As a result, the evader drops to the third position in the ranking produced by the Degree source detection algorithm, and to the fifth position in the ranking produced by the Closeness source detection algorithm, thereby concealing their identity as the source of diffusion. As can be seen, by performing a relatively small number of network modifications, the evader can lower their chances of being identified as the source. It should be noted that the scenario illustrated in Figure 1 is just an example; in principle, the added nodes do not necessarily have to be connected to the source’s neighbors, but can be connected to any other node in the network. Likewise, modifying the network edges does not have to be done by removing existing ones; it could also be done by adding edges to the network. The effectiveness of the different choices will be evaluated in our subsequent experiments.

The first question we investigate is: *How difficult is it to find an optimal way of hiding the source of diffusion?* See STAR Methods for formal definitions of the decision problems faced by the evader. Our theoretical findings regarding the computational complexity of these problems are summarized in Table 1, while the formal proofs can be found in the study by Waniek et al. (2021a). As can be seen in the table, in almost all cases, the problems under consideration are NP-complete (Non-deterministic Polynomial-time complete), implying that no known algorithm can solve them in polynomial time relative to the network size. Hence, finding an optimal way of preventing the evader from being identified as the source of diffusion is a computationally intractable task that cannot be completed efficiently, especially for large networks. It is worth noting that we formulated the problem faced by the evader as a decision version, i.e., identifying whether it is possible to achieve a certain level of concealment from source detection algorithms given a certain budget. The problem faced by the evader can also be formulated as an optimization version, i.e., identifying the budget required to achieve a certain level of concealment. We decided to focus on the decision version, as it is the most popular framework of computational complexity analysis (Arora and Barak, 2009).

**Table 1:** Summary of our computational complexity results

Source detection algorithm	Adding nodes	Modifying edges
Degree	P	NP-complete
Closeness	NP-complete	NP-complete
Betweenness	NP-complete	NP-complete
Rumor	NP-complete	NP-complete
Random Walk	NP-complete	NP-complete
Monte Carlo	NP-complete	NP-complete

For different source detection algorithms, we consider the decision problem that the evader must solve in order hide optimally from the algorithm by either adding nodes or modifying edges. P = solvable in polynomial time; NP-complete = Non-deterministic Polynomial-time complete, implying that no known algorithm can solve it in polynomial time.

### Heuristics

Given the computational complexity of identifying an optimal way of hiding the source of diffusion, we will now focus on heuristic methods instead. The first class of heuristics that we consider is adding confederates to the network. We use the term “supporters” to describe the nodes that are already present in the network and are willing to accept connections from the confederates. These supporters do not necessarily need to be the evader’s associates, and are not restricted to those who are intentionally cooperating to hide the source. Then, the evader must optimize the list of contacts of each confederate, which can include any of the supporters and any of the other confederates. Let us first consider how contacts are chosen from the list of supporters. Assuming that the evader wishes to connect each confederate to k supporters, we consider three alternative heuristics:

• **Hub**—for each confederate, connect it to the k supporters with the greatest degrees (this way, all confederates get connected to the same k supporters);
• **Degree**—for each confederate, connect it to the k supporters with the greatest degree out of those who are not yet connected to any other confederate (if no such supporters exist, select from the ones connected to the smallest number of confederates);
• **Random**—for each confederate, connect it to k supporters chosen uniformly at random.
Each of the above heuristics has two versions, depending on how the confederates are connected to each other. In particular, we consider two possibilities:
• **Just supporters**—every confederate is connected only to supporters, implying that there are no edges between confederates;
• **Clique**—every confederate is connected to every other confederate, implying that the confederates form a clique.

For each heuristic, we add the word “clique” to indicate that the confederates are connected to each other, e.g., by writing “Hub clique”. Otherwise, if there are no connections between the confederates, we write the name as it is, e.g., “Hub”.

Now that we have presented our first class of heuristics, which add confederates to the network, let us now consider the second class of heuristics, which modify edges in the network. We assume that the evader can only add or remove edges between themselves and a specific subset of nodes. To determine which of those nodes to connect to, and which to disconnect from, we consider three alternative heuristics:

• **Max degree**—choose the nodes with the greatest degree;
• **Min degree**—choose the nodes with the smallest degree;
• **Random**—choose nodes uniformly at random.

All ties are broken uniformly at random. Each of the above heuristics has two versions, depending on whether the evader is adding new connections, or removing existing ones. We write the word “adding” to indicate that the evader is adding new connections, e.g., by writing “Adding max degree”. Otherwise, we write the word “removing” to indicate that the evader is removing existing connections, e.g., “Removing max degree”.

It is worth noting that all heuristics that we consider in our experiments are generic, i.e., they are not designed to mislead a specific source detection algorithm, but rather have a fighting chance against a wide variety of them. We focus on this class of heuristics as we assume a situation in which the evader may not be sure what tools and techniques are going to be used by the seeker to identify the source of diffusion. Thus, the evader would be more interested in a technique effective against a portfolio of source detection algorithms, rather than dedicated to counteracting only one of them. Designing such highly focused countermeasures is a potential direction of future work.

### Simulation analysis

The experimental procedure for a given network G=(V,E) is as follows. First, we select the evader $v^{†}$ uniformly at random from the top 10% of nodes according to degree ranking, provided that its degree is at least 10. We imposed this restriction because some of our heuristics remove edges that are incident to the evader, implying that the evader needs to have enough edges to actually execute these heuristics. Besides, the spreaders of fake news tend to be well connected (Bovet and Makse, 2019). After the evader is selected, we spread the diffusion starting from $v^{†}$ to obtain the set of infected nodes I. In our simulations, we use the SI model with the probability of diffusion being p=0.15 and the number of rounds being T=5. We then perform the hiding process using different heuristics, recording the position of $v^{†}$ in the rankings generated by each source detection algorithm after each step of the hiding process.

The majority of the source detection algorithms considered in our experiments disregard the nodes that are not infected. Hence, when choosing the confederate’s contacts, it makes sense for the evader to consider only infected supporters. In our simulations, we assume that all infected nodes other than the evader are supporters. Furthermore, we assume that the evader can only remove edges that they are part of and only add edges between themselves and their neighbors’ neighbors.

**Figure 2:**
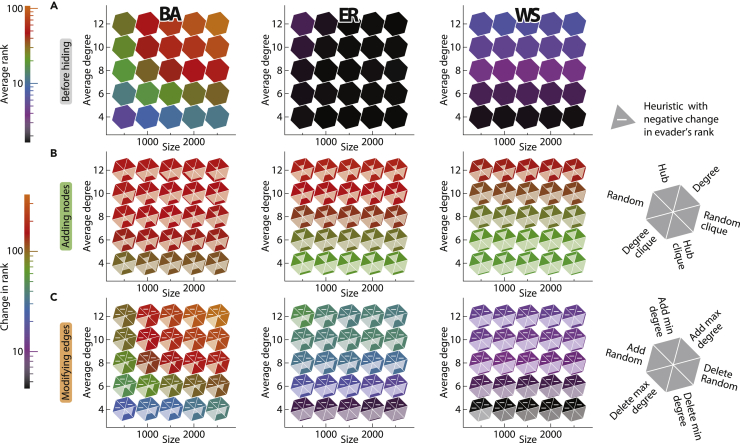
The efficiency of hiding from the Eigenvector source detection algorithm in networks with varying structure, size, and density In each color-coded tessellation, the x axis represents the number of nodes in the network, while the y axis represents the average degree. The first column corresponds to scale-free networks generated using the Barabási-Albert (BA) model, the second corresponds to random networks generated using the Erdős-Rényi (ER) model, and the third corresponds to small-world networks generated using the Watts-Strogatz (WS) model. The results are presented as an average over 100 networks and over 10 evaders in each network. The colors of the first row (A) give the average position of the evader in the ranking computed using the Eigenvector source detection algorithm before the hiding process on a logarithmic scale (the lower the ranking, the more exposed is the evader). The second (B) and third (C) rows depict the average difference in the evader’s ranking as a result of the hiding process, where each hexagon indicates the efficiency of six different hiding heuristics, and the color represents the effectiveness of the best heuristic (on a logarithmic scale). More specifically, row (B) depicts the effect of adding 50 confederates connected to 3 supporters each, while row (C) depicts the effect of either adding or removing 5 edges, depending on the heuristic being used. Additionally, each triangular sector is filled up with a lighter shaded area (overlaid the hexagons), representing the relative effectiveness of the corresponding heuristic compared to the best one (the greater the shaded area, the better the heuristic). As such, triangular sectors that have no shaded area have zero effectiveness. However, there are cases where the effectiveness is even less than zero, i.e., it backfires and ends up exposing the evader even more. In such cases, the triangular sector has no shaded area and is also marked with a minus.

In our experiments, we will first disentangle two different aspects of hiding. The first aspect relates to the network topology itself, which can provide some concealment even without the evader manipulating it. The second aspect comes from an evader strategically manipulating the network after the inception of the diffusion process. To separate the two notions of hiding, we ran experiments on networks with varying structure, size, and density; see Figure 2. The figure presents the results for the Eigenvector source detection algorithm, in particular. We chose this example since it is one of the best-performing algorithms and yields the most pronounced differences between the best and worst hiding heuristics; see Figures S1–S6 for the results pertaining to other source detection algorithms. Figure 2A presents the results for the first notion of hiding, i.e., the one stemming from the network structure itself, whereas Figures 2B and 2C present the results for the second notion of hiding, which stems from strategically adding confederates or modifying edges, respectively. In all of our simulations, we report the absolute, rather than relative, ranking of the evader according to the source detection algorithm in question. However, in principle, one could suspect an individual to be the source of diffusion if that individual is, e.g., among the top 10 nodes, or the top 1% of nodes. Since the two are correlated, we chose one of them—the absolute ranking—and used it throughout all of our experiments.

As can be seen in Figure 2A, out of the three network structures considered in our experiments—scale-free (Barabási and Albert, 1999), small-world (Watts and Strogatz, 1998), and random (Erdős and Rényi, 1959)—the one that provides the greatest level of concealment to the evader is the scale-free structure. Moreover, independent of the network model, the denser the network, the more concealed is the evader. As for the network size, having a larger number of nodes results in a greater level of concealment for scale-free networks, but results in a negligible effect for small-world or random networks. When it comes to strategic hiding via network manipulations, Figures 2B and 2C show that it is generally more efficient to strategically hide in networks with greater density. When comparing the different structures in terms of how they facilitate the strategic hiding, our heuristics are most efficient in scale-free networks, and least efficient in small-world networks, regardless of whether the evader is adding confederates, or modifying edges. Finally, commenting on how the network size affects the efficiency of strategic hiding, the effect is relatively small. The only exception is when hiding by modifying edges in scale-free networks, which is considerably more effective in larger networks. Next, we compare heuristics of the same type, starting with the ones that add confederates, to determine whether they should create a clique among themselves or remain disconnected from one another, and determine which supporters to connect to which confederates. As for the former question, creating a clique is consistently superior (see how the shaded area of the triangular sectors in Figure 2B is greater for heuristics with “clique” in their name). As for the question of which supporters to connect to which confederates, when confederates form a clique, it is more fruitful to connect confederates to different supporters (using either the Random clique or Degree clique heuristics) than connecting them all to the same supporters (using the Hub clique heuristic). On the other hand, when confederates are disconnected from each other, the results vary depending on the source detection algorithm being used; see Figures S1–S6. Having compared the heuristics that add confederates, we now compare the heuristics that modify (some of) the edges that are incident to the evader, to determine whether we should add or remove edges. Our results indicate that the latter is significantly more effective. In fact, adding new edges often backfires, and ends up exposing the evader even more to the source detection algorithm. The only remaining question is to determine which edges to remove from the network. Our results show that the most effective choice is to disconnect the evader from the neighbors with the greatest degrees, and the least effective choice is to disconnect from those with the lowest degrees. All the results in Figure 2 are shown after the heuristics have made all the modifications to the network. Figures S7–S14 are alternative versions of Figure 2 that are meant to facilitate an easier comparison between different heuristics, network structures, and source detection algorithms. To see how the evader’s ranking changes after each such modification, see Figures S15–S17 for the heuristics that add confederates, and Figures S18–S22 for the heuristics that modify edges. Moreover, Figures S7–S22 contain results for networks with exponential degree distribution $P\left(d\right)∼d^{-2.3}$ generated using the Newman configuration model (Newman, 2003). In general, results for these networks show similar trends to Barabási-Albert networks, since they are different variations of scale-free networks with power-law degree distribution (with the Barabási-Albert model exhibiting a preferential-attachment structure).

**Figure 3:**
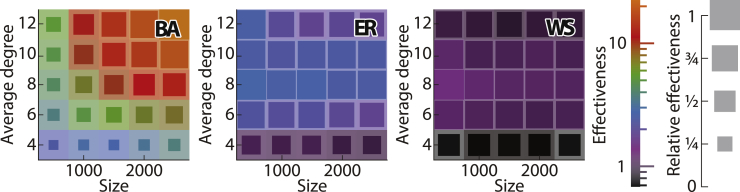
Comparing the effectiveness of adding confederates vs. modifying edges when hiding from the Eigenvector source detection algorithm The effectiveness of modifying an edge is calculated as the number of confederates that must be added to achieve the same change in the evader’s ranking (assuming that the modification of edges and the addition of confederates are done using the best respective heuristics). The heatmaps evaluate the effectiveness of modifying an edge while varying the network structure. In particular, the leftmost heatmap corresponds to scale-free networks generated using the Barabási-Albert (BA) model, the central heatmap corresponds to random networks generated using the Erdős-Rényi (ER) model, and the rightmost heatmap corresponds to small-world networks generated using the Watts-Strogatz (WS) model. In each heatmap, the x axis represents the number of nodes in the network, while the y axis represents the average degree. The color of each cell corresponds to the average effectiveness of modifying an edge. The size of the unshaded (darker) area in each cell corresponds to the average effectiveness of modifying an edge in that cell, relative to the maximum effectiveness across all cells of that panel. The results are presented as an average over 100 networks and over 10 evaders in each network, after adding 50 confederates or modifying 5 edges. The colors in the heat maps reflect a logarithmic scale.

It is difficult to compare the effectiveness of adding nodes and modifying edges based solely on Figure 2, since the figure shows only the impact of adding 50 nodes and modifying 5 edges. To facilitate this comparison, Figure 3 shows how many nodes must be added to the network in order to have the same effect as modifying a single edge. As can be seen, in the majority of cases, the effect of modifying a single edge is equivalent to adding several confederates. In fact, the number of confederates needed to achieve the same effect as modifying a single edge is surprisingly large (and may even reach tens) in scale-free networks. The only exception is the case of sparse, small-world networks, where adding one confederate affects the evader’s ranking more than modifying a single edge (as indicated by the values smaller than 1 in the heatmap). The results depicted in the figure are for the Eigenvector source detection algorithm; the results for other source detection algorithms are qualitatively similar as shown in Figure S23.

**Figure 4:**
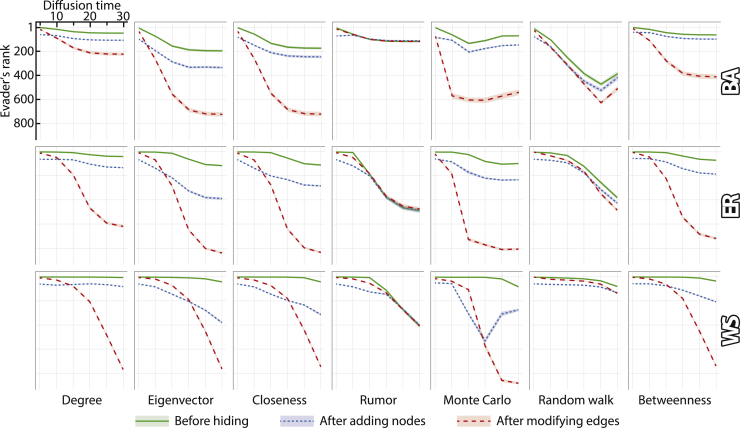
The impact of extending the diffusion time on the effectiveness of hiding In each plot, the x axis represents the diffusion time, i.e., the total number of rounds in the diffusion process. The y axis represents the evader’s ranking according to the source detection algorithm; this ranking is computed before the hiding process (green line), after running the best heuristic that adds confederates (blue line), and after running the best heuristic that modifies edges (red line). Each column corresponds to a different source detection algorithm, while each row corresponds to a different model used to generate the network in which the diffusion takes place. Results are averaged over 10 evaders and over 100 networks generated using either the Barabási-Albert (BA), the Erdős-Rényi (ER), or the Watts-Strogatz (WS) model, consisting of 1,000 nodes each, with the average degree being 4. The axes in all plots are identical to those used in the upper-left corner. Shaded areas (which are often too small to see) represent 95% confidence intervals.

Another aspect that may impact the effectiveness of hiding the evader is the diffusion time, i.e., the total number of rounds completed in the diffusion process before the source detection algorithm analyzes the network. The results of this analysis can be found in Figure 4. In the vast majority of cases, the evader becomes more hidden as diffusion time increases. This is true not only when the evader performs no modifications to the network but also when they modify the network by adding confederates or by removing edges following the most effective heuristic (although the effectiveness of removing edges grows at a greater rate than that of adding confederates). This suggests that if our goal is to identify the source of diffusion, we should start our investigation as early as possible. Shah et al. (2020) reported similar findings, but for different diffusion models than ours, namely Susceptible-Infected-Recovered (SIR) and Susceptible-Exposed-Infected-Recovered. Next, we compare the source detection algorithms to each other. As can be seen from the figure, the diffusion time’s sensitivity varies greatly from one algorithm to another. When the evader performs no modifications, the Degree and Betweenness algorithms prove to be the most resilient. Similar results are observed when the evader adds confederates to the network. In contrast, when modifying edges, the most resilient algorithms are Rumor and Random Walk, i.e., they are the least affected by changing the diffusion time. Interestingly, all three types of network structures show relatively similar patterns, suggesting that the source detection algorithms’ inner workings play a more important role in determining how the diffusion time affects the effectiveness of hiding, rather than the network characteristics.

**Figure 5:**
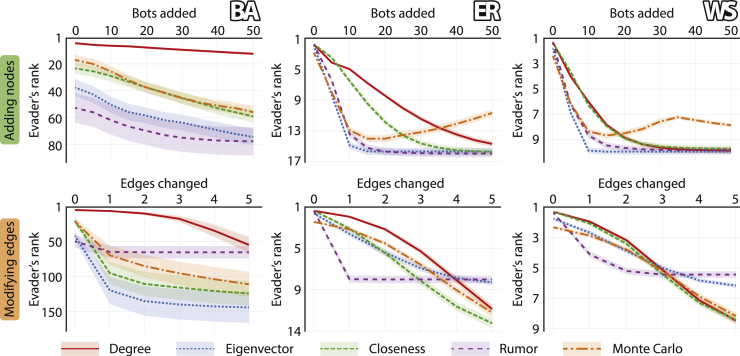
The effectiveness of hiding in massive networks Given networks of 100,000 nodes with an average degree of 4, the figure depicts the evader’s ranking (y axis) as a function of the number of network modifications (x axis), using the best heuristic for adding nodes (first row) or modifying edges (second row). Different colors represent different source detection algorithms. The results are presented as averages taken over 100 networks generated using either the Barabási-Albert (BA), the Erdős-Rényi (ER), or the Watts-Strogatz (WS) model, and over 10 different evaders in each network. Shaded areas represent 95% confidence intervals.

So far in our analysis, we only considered networks of up to 2,500 nodes, since the analysis involved taking an average over a large number of cases, and increasing the number of nodes would have taken excessive time. Fortunately, when it comes to evaluating the impact of the hiding process, we can approximate it even for massive networks. Based on this, we increased the number of nodes to 100,000, and approximated the evader’s ranking after each step of the hiding process. The approximation is done by computing the ranking of the evader not among all nodes, but rather among 10,000 nodes, consisting of the 5,000 infected nodes with the greatest degrees and another 5,000 infected nodes chosen uniformly at random from the remaining ones. Furthermore, in our approximation, we do not consider the Betweenness and Random walk source detection algorithms, since their ranking cannot be efficiently computed for just a selected subset of nodes. The results of this analysis are presented in Figure 5. In networks generated using the Erdős-Rényi model and the Watts-Strogatz model, the evader usually occupies the top position of the ranking before hiding, whereas in networks generated using the Barabási-Albert model, they are in the top 50 positions. Moreover, in the former two types of networks, the hiding process seems much less effective than in the latter, regardless of whether the hiding is done by adding nodes or by modifying edges. Notice that the effectiveness of hiding in these massive networks is considerably reduced compared to smaller networks with 1,000 nodes, the results for which were presented in previous figures. These findings are all based on the best heuristic of each type; see Figures S24–S28 for an evaluation of the remaining heuristics.

**Figure 6:**
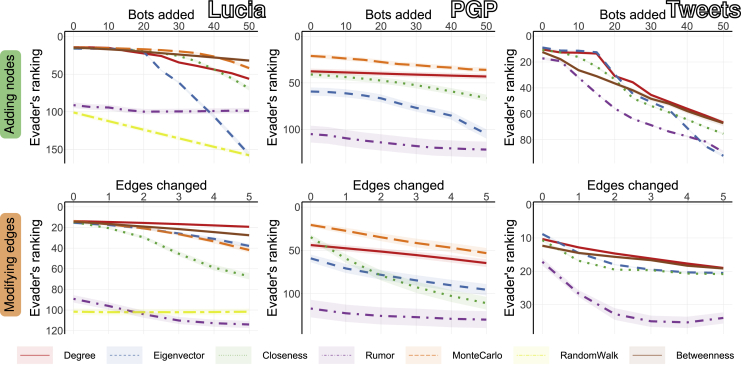
The effectiveness of hiding in real-life networks Given various real-life networks, the figure depicts the evader’s ranking (y axis) as a function of the number of network modifications (x axis), using the best heuristic for adding nodes (first row) or modifying edges (second row). Different colors represent different source detection algorithms. The results are presented as averages taken over 100 different evaders in each network. Shaded areas represent 95% confidence intervals.

All findings presented thus far are based on cascades taking place in randomly generated networks. In contrast, Figure 6 presents the results of hiding from source detection algorithms in real-life networks. The first structure that we consider is a colocation network collected at the St Lucia campus of the University of Queensland (Thomas et al., 2016) (302 nodes and 1149 edges), where nodes represent students, while an edge between any two students indicates that they were in the same location at the same time. A diffusion process in this network could be an infection that requires physical proximity to spread. The second network represents the trust relationships of the Pretty-Good-Privacy (PGP) algorithm for secure information exchange (Boguná et al., 2004) (10,680 nodes and 24,316 edges). Nodes represent users of the algorithm, while an edge between two nodes indicates that they mutually signed their public keys within the protocol. A diffusion process in this network could represent a security breach at the source node spreading to users connected by trust relationships. Admittedly, the above two examples represent case studies in which the networks under consideration are real, but the cascades are not, since they are generated using the SI model. The last column of Figure 6 analyzes a more realistic case in which both the network and the cascade are real. To this end, we use the Twitter dataset created by Mønsted et al. (2017) (241,698 nodes and 366,539 edges), where each node corresponds to a Twitter account, and an edge between two nodes indicates that one is a follower or a friend of the other, or that one retweeted content posted by the other. In this real-life example, the authors started spreading eight hashtags that previously did not exist on Twitter, capturing realistic social media information cascades. Altogether, the results for the above three real-life networks seem to be largely consistent with observations made earlier for random networks. In some cases, the evader is hidden even without any strategic manipulations, suggesting that correctly identifying the source of diffusion in the real world might be even harder than our simulations on synthetic data indicate. Moreover, our heuristics are capable of reducing the effectiveness of source detection algorithms in these real-life networks. Figures S29–S38 present results for additional real-life networks (while using the SI model), and Figures S39–S41 present more detailed results where both the network and the cascades are taken from real data.

We conclude our analysis by exploring different variations of the experimental setup. First, we ran simulations with alternative models of diffusion and network generation (Figures S42–S45); the results were qualitatively similar to those obtained from our main experimental setup. Second, we investigated how the evader’s ranking is affected when another node, v, located in the evader’s direct network vicinity runs our heuristics (Figure S46); we found that the evader becomes hidden as a result ofv’s actions, albeit not as effectively as in the case when the evader is the one running the heuristic. Third, we investigated how the completeness of the seeker’s knowledge about the network’s structure affects the effectiveness of the evader’s hiding (Figure S47); we found that in the vast majority of cases the evader’s hiding becomes significantly more effective as the knowledge of the seeker dwindles. Fourth, instead of selecting the evader randomly from the 10% of nodes with the highest degrees (as in our basic experiments), we analyzed the case where the evader is randomly selected out of all nodes (Figures S48–S52). The results were broadly similar to those observed in our basic experiments, i.e., when the evader does not try to hide, they are ranked high according to source detection algorithms, but when the evader runs the hiding heuristics, they are able to significantly reduce the likelihood of being identified. Fifth, we performed various sensitivity analyses by modifying the parameters used in our original simulations. To this end, we started by analyzing how the hiding process is affected by the way in which the evader is selected. Specifically, instead of selecting the evader from the top 10% of nodes with the highest degrees (as was the case with our main experiments), we selected the evader from the top x% where 10≤x≤90; the results suggest that the value of x has a relatively small effect on the outcome (Figures S53 and S54). Next, instead of setting the rewiring probability of the Watts-Strogatz model to be 0.25, we varied this probability from 0.05 to 0.5, and found that the hiding process tends to be slightly more effective given greater values of this parameter (Figure S55). Finally, instead of approximating the evader’s ranking by computing it among 10,000 infected nodes, we varied this number between 2,000 and 18,000, and found this to have a relatively small effect on the outcome (Figures S56 and S57). See STAR Methods for detailed descriptions of the variations of the experimental setup.

## Discussion

In this work, we analyze the possibility of obfuscating the origin of diffusion, both as a result of spreading it in a specific type of network structure, and via strategic network manipulations. On the one hand, our theoretical analysis indicates that finding an optimal way of hiding the source of diffusion, either by adding confederates or by modifying the networks’ edges, is computationally intractable. On the other hand, our computational experiments demonstrate that even without any strategic manipulations, the structure of the network itself can greatly hinder the efforts to identify the diffusion source. This seems to be the case especially in scale-free networks—an observation that is particularly alarming since many real-life social networks exhibit this property. We also find that the task of identifying the source of diffusion is more challenging in networks that are dense, allowing the culprit to hide in the crowd. Moreover, an adversarial agent can utilize several network modifications simultaneously to obfuscate the source even more. Particularly effective strategies in this regard are based on attaching a densely connected group of confederates to the network and removing connections between the source of the diffusion and its most well-connected neighbors after the diffusion has started. Our analysis also confirms previous findings derived from alternative diffusion models (Shah et al., 2020), indicating that the longer the diffusion takes, the more difficult it is to pinpoint its origin, highlighting the importance of prompt reaction to any potential epidemic threat. Finally, our experiments indicate that finding the diffusion source is easier in massive, sparse networks, regardless of whether the evader strategically manipulates the network to hide its identity. Altogether, regardless of the network or the diffusion model under consideration, we found that either the source of the diffusion is well hidden without any strategic modifications, concealed by the structure of the network itself, or that there exists at least one effective yet simple heuristic solution allowing the source to avoid detection. These universal patterns imply that, given current source detection algorithms, if the diffusion source tries to hide, it probably will succeed. Future algorithms will need other types of information capturing the time evolution of both the diffusion and the network.

There exists a growing literature on avoiding detection by a wide range of social network analysis tools. Such hiding techniques can be used to prevent a closely cooperating group of nodes from being identified by community detection algorithms (Waniek et al., 2018), or prevent the leader of the organization from being recognized by centrality measures in standard (Waniek et al., 2017, 2021b; Was et al., 2020), multilayer (Waniek et al., 2020), and temporal (Waniek et al., 2022) networks. Similar techniques can be used to prevent an undisclosed relationship from being exposed by link prediction algorithms (Waniek et al., 2019; Zhou et al., 2019). Nevertheless, none of the existing works considered strategically hiding the source of diffusion from source detection algorithms. What is more, they typically only study hiding by adding edges to, and removing edges from, a network while disregarding the possibility of avoiding detection by adding nodes to the network. Another body of work that is relevant to our study is the one considering the reconstruction of diffusion cascades. Typically in this literature, the party analyzing the network has at its disposal more information than what is available to the seeker in our setting. Specifically, many works require knowledge about either the exact moment when each node was reached by the diffusion process (Taxidou and Fischer, 2014; Farajtabar et al., 2015; Xiao et al., 2018b) or the order in which the nodes became infected (Ghosh and Lerman, 2011). Others consider settings where available data include edges over which the diffusion took place (Sadikov et al., 2011). Some studies assume that the underlying structure is a temporal network (Rozenshtein et al., 2016), where each edge exists only in a specific moment in time, or assume the availability of an API serving data about the diffusion (Cogan et al., 2012). Yet another body of literature tries to infer which nodes are actually in the infected state, based on partial information (Xiao et al., 2018a; Sundareisan et al., 2015). Finally, we mention a growing literature focusing on the reconstruction of the network structure based on information about diffusion cascades (Shen et al., 2014; Gomez Rodriguez et al., 2013; Braunstein et al., 2019; Pajevic and Plenz, 2009; Chwistek and Butail, 2020; Li and Li, 2017).

In our study, we kept the models, the simulations, and the theoretical analysis as generic as possible, without making assumptions about the nature of the social diffusion under consideration. Nevertheless, it should be noted that the effectiveness of our heuristic depends on the specifics of the scenario at hand. For instance, hiding certain facts (such as coming in contact with a certain individual) might be easier if the diffusion is taking place in the real world as opposed to the virtual world. Consider social media platforms such as Twitter or Facebook, where all the online activities are logged by the platform. In such cases, the source can no longer hide its actions from the platform administrators (since they have access to these logs). Having said that, the source may still be able to hide from other observers who cannot access such logs and can only view publicly available information. Another noteworthy aspect of our analysis is that we only consider source detection from the perspective of identifying the node (e.g., the Facebook account) that initiated the diffusion process. This should not be confused with the problem of identifying the actual entity represented by the source node (e.g., the person or organization behind the Facebook account); such identification is out of the scope of our study. Finally, we comment on the applicability of source detection algorithms in the real world. As mentioned earlier, we avoided incorporating domain-specific details in our experiments to keep the implications as broad as possible. In practice, however, we suspect that source detection algorithms would be augmented by additional, domain-specific information. Having said that, our evaluation of these algorithms on real-life cascades suggests that, even without such information, the algorithms can be effective, at least in narrowing down the search for the source.

The direct policy implication of our work is that one has to be sure the source has not been trying to hide itself to trust the source detection algorithms of today. This is because, as our experiments have shown, such algorithms can easily be fooled by a strategic source who is actively attempting to escape detection. Having said that, our work also points to the future—the necessary elements of the next generation’s source detection algorithms. In particular, because hiding by manipulating edges is so efficient compared to adding fake nodes, new algorithms need to identify spurious links—connections added since the start of the diffusion. Arguably, the need for such algorithms is more pressing than ever. Developing tamper-proof source detection algorithms would improve our chances of detecting the source of a viral cascade in the future.

### Limitations of the study

We now discuss the limitations of our approach.

First, although we assume that the evader is strategic (i.e., it is aware of the analysis conducted by the seeker and acts strategically to mislead this analysis), the seeker is assumed to be non-strategic. That is, the seeker simply applies a particular source detection algorithm without considering potential attempts made by the evader to escape detection. Introducing a strategic seeker would turn our model into a game-theoretic setting, where both the seeker and the evader employ different strategies trying to outsmart one another. The analysis of this kind of game requires a vastly different set of techniques than these employed in our work, and we leave it as a potential future extension.

Second, we assume that the knowledge of the seeker is limited to the topology of the network and the state of each node (i.e., whether it is susceptible or infected). The reason behind this assumption is that this is precisely the information required by the source detection algorithms that we take into consideration in our work. Nevertheless, there might exist situations in which the seeker has access to additional, domain-specific information that might be utilized to improve the source detection methods.

Third, while we quantify the effectiveness of various heuristic solutions using simulations, we do not provide any worst-case guarantees on their performance. In some cases, it is possible to prove that a solution computed by a given algorithm is guaranteed to be within certain bounds from the optimal solution. However, obtaining this kind of result often requires making additional assumptions about the network under consideration, whereas we prefer to focus on as general a setting as possible.

## STAR★Methods

### Key resources table

**Table:** 

REAGENT or RESOURCE	SOURCE	IDENTIFIER
**Deposited data**		

Code	This paper	https://github.com/mjwaniek/HidingSource
St Lucia dataset	Thomas et al. (2016)	https://github.com/brycethomas/bthomas2016plosone
Facebook dataset	Leskovec and Mcauley (2012)	https://snap.stanford.edu/data/ego-Facebook.html
Copenhagen dataset	Ahmed et al. (2010)	https://nodexlgraphgallery.org/Pages/Graph.aspx?graphID=260916
PGP dataset	Boguná et al. (2004)	http://konect.cc/networks/arenas-pgp/
Prostitution dataset	Rocha et al. (2010)	http://konect.cc/networks/escorts/
Twitter dataset	Mønsted et al. (2017)	http://sodas.ku.dk/contact/

**Software and algorithms**		

Java version 16.0.2	Oracle	https://www.oracle.com/java/
R version 4.1.1	R Foundation for Statistical Computing	https://cran.r-project.org/

### Resource availability

#### Lead contact

Further information and requests for materials should be directed to and will be fulfilled by the lead contact, Talal Rahwan (talal.rahwan@nyu.edu).

#### Materials availability

This study did not generate new unique reagents.

### Experimental model and subject details

This work does not use experimental models typical in the life sciences.

### Method details

#### Basic network notation

Let us denote by G=(V,E)∈G a network, where V is the set of n nodes and E⊆V×V is the set of edges. We denote an edge between nodes v and w by (v,w), and we only consider *undirected* networks, implying that we do not discern between edges (v,w) and (w,v). Moreover, we assume that networks do not contain self-loops, i.e., $\forall_{v\in V}\left(v,v\right)\notin E$. We denote by $\bar{E}$ the set of all non-edges, i.e., $\bar{E}=\left(V\times V\right)\setminus \left(E\cup \cup_{v\in V}\left(v,v\right)\;\right)$. A path in a network G=(V,E) is an ordered sequence of distinct nodes, $\left\langle v_{1},\ldots ,v_{k}\right\rangle$, in which every two consecutive nodes are connected by an edge in E. We consider the length of a path to be the number of edges in that path. The set of all shortest paths between a pair of nodes, v,w∈V is denoted by $\Pi_{G}\left(v,w\right)$, while the distance between a pair of nodes v,w∈V, i.e., the length of a shortest path between them, is denoted by $d_{G}\left(v,w\right)$. Furthermore, a network is said to be *connected* if and only if there exists a path between every pair of nodes in that network. We denote by $N_{G}\left(v\right)$ the set of *neighbors* of v in G, i.e., $N_{G}\left(v\right)=\left\{w\in V:\left(v,w\right)\in E\right\}$. We denote by $G^{V^{\prime }}$ the subnetwork of G induced by the nodes in $V^{\prime }\subseteq V$, i.e., $G^{V^{\prime }}=\left(V^{\prime },E\cap \left(V^{\prime }\times V^{\prime }\right)\right)$. Finally, for $E^{\prime }\subseteq V\times V$ we denote by $G\cup E^{\prime }$ the effect of adding set of edges $E^{\prime }$ to G, i.e., $G\cup E^{\prime }=\left(V,E\cup E^{\prime }\right)$. To make the notation more readable, we will often omit the network itself from the notation whenever it is clear from the context, e.g., by writing d(v,w) instead of $d_{G}\left(v,w\right)$. This applies not only to the notation presented thus far, but rather to all notation in this article.

#### Susceptible-Infected model and source detection algorithms

In the Susceptible-Infected (SI) model, every node in the network is in one of two states: either susceptible (prone to be affected by the phenomenon) or infected (already affected by the phenomenon). The modeled process consists of discrete rounds. At the beginning of the process only the nodes belonging to the *seed set* are in the infected state (in this work, the seed set consists of only the evader $v^{†}$). In every round t, every infected node makes each of its susceptible neighbors infected with probability p. The process ends after a certain number of rounds T. We denote the set of infected nodes after T rounds by I.

A source detection algorithm is a procedure that, based on the network G and the set of infected nodes I, aims at determining the source of diffusion. Every source detection algorithm considered in this work can be represented as a function $\sigma :V\times G\times 2^{V}\to R$ that assigns the score σ(v,G,I) to any node v, where the node with the highest score is selected by the algorithm as the most probable source of diffusion. We will assume that for any node v∉I and any source detection algorithm we have σ(v,G,I)=−∞ (as the seed node has to be infected and there is no mechanism of coming back to the susceptible state). In this work we focus on the source detection algorithms that are designed to detect the source of a diffusion process with a seed set consisting of only one node (see Shelke and Attar (2019) for a review of multiple source detection algorithms). More specifically, we consider the following source detection algorithms:

• Degree (Comin and Costa, 2011)—the score assigned to a given v∈I is the degree centrality of v in $G^{I}$, i.e.:

$$\sigma_{degr}\left(v,G,I\right)=\left|N_{G^{I}}\left(v\right)\right|;$$

• Closeness (Comin and Costa, 2011)—the score assigned to a given v∈I is the closeness centrality of v in $G^{I}$, i.e.:

$$\sigma_{clos}\left(v,G,I\right)=\frac{1}{\sum_{w\in I}d_{G^{I}}\left(v,w\right)};$$

• Betweenness (Comin and Costa, 2011)—the score assigned to a given v∈I is the betweenness centrality of v in $G^{I}$, i.e.:

$$\sigma_{betw}\left(v,G,I\right)=\sum_{u\neq w:u,w\in I\backslash \left\{v\right\}}\frac{\left|\left\{\pi \in \Pi_{G^{I}}\left(u,w\right):v\in \pi \right\}\right|}{\left|\Pi_{G^{I}}\left(u,w\right)\right|};$$

• Eigenvector (Comin and Costa, 2011)—the score assigned to a given v∈I is the eigenvector centrality of v in $G^{I}$, i.e.:

$$\sigma_{eig}\left(v,G,I\right)=x_{v}$$

where x is the eigenvector corresponding to the largest eigenvalue of the adjacency matrix of $G^{I}$;

• Rumor (Shah and Zaman, 2011)—the score assigned to a given v∈I is the rumor centrality of v in $G^{I}$, i.e.:

$$\sigma_{rumor}\left(v,G,I\right)=\frac{\left|I\right|!}{\prod_{w\in I}\Theta_{w}^{v}}$$

where $\Theta_{w}^{v}$ is the size of the subtree of w in the BFS (Breadth-First Search) tree of $G^{I}$ rooted at v;

• Random Walk (Jain et al., 2016)—intended to approximate diffusion by random walks. The score of a given node v is:

$$\sigma_{rwalk}\left(v,G,I\right)=\left\{ \begin{array}{ll} \varphi_{0}\left(v\right) & \text{if }\forall_{w\in I}d_{G}\left(v,w\right)\leq T\\ 0 & \text{otherwise} \end{array}\right.$$

where T is the number of rounds in the SI model and φ is as:

$$\varphi_{t}\left(v\right)=\left\{ \begin{array}{ll} 1 & \text{if }t=T\\ \left(1-p\right)\varphi_{t+1}\left(v\right)+\sum_{w\in N\left(v\right)\cap I}\;\frac{p}{\left|N\left(v\right)\right|}\varphi_{t+1}\left(w\right) & \text{otherwise} \end{array}\right.$$

where p is the probability of infection in the SI model.

• Monte Carlo (Antulov-Fantulin et al., 2015)—where for each node we repeated run a diffusion starting with that node and investigate for which of the nodes the infected set is the most similar to I (using Jaccard similarity). The score of a given node v is:

$$\sigma_{mcarlo}\left(v,G,I\right)=\frac{1}{m}\sum_{i=1}^{m}\exp\left(\frac{-{\left(\psi_{J}\left(I,I_{v,i}\right)-1\right)}^{2}}{a^{2}}\right)$$

where m is the number of Monte Carlo samples for each node, $\psi_{J}\left(A,B\right)=\frac{\left|A\cap B\right|}{\left|A\cup B\right|}$ is the Jaccard similarity measure, $I_{v,i}$ is the set of infected nodes in the i-th Monte Carlo sample where the diffusion starts with v, and a is the soft margin parameter.

There also exist more advanced source detection algorithms that are specifically designed to find the source of diffusion in tree networks (Wang et al., 2014, 2015; Cai et al., 2018). However, applying them to general (i.e., cyclic) networks is exceedingly expensive in terms of computation time even for very small structures (Antulov-Fantulin et al., 2015). Other algorithms use different frameworks than the one considered in our work, where they analyze the problem of placing a number of sensors in a network that notify the user when diffusion reaches a specific node (Pinto et al., 2012; Xu and Chen, 2015; Paluch et al., 2018).

#### Formal definitions of the decision problems

We now formally define the computational problems faced by the evader. In what follows, let #(v,G,σ,I) denote the ranking position of v among all nodes in G according to source detection algorithm σ when the set of infected nodes is I. More formally:

#(v,G,σ,I)=|{w∈V:σ(w,G,I)>σ(v,G,I)}|.

The goal of the evader is to hide by decreasing their position in the ranking produced by σ (notice that decreasing the position in the ranking corresponds to maximizing the value of #(v,G,σ,I)).

The first method of hiding that we consider is to add confederates to the network. Then, the problem faced by the evader is to determine the contacts of each confederate. Since not every node in the network is necessarily willing to accept connections from these confederates, we define a subset of nodes, $\hat{S}\subseteq V$, that would accept such connections. More formally, the problem can be defined as follows:

#### Definition 1 (hiding source by adding nodes)

*The problem is defined by a tuple,*$\left(G,v^{†},I,\sigma ,\omega ,b,\nabla ,\hat{S}\right)$, where G=(V,E)*is a network,*$v^{†}\in V$ is the evader, I⊆V *is the set of infected nodes,* σ *is a source detection algorithm,* ω∈N *is a safety threshold specifying the smallest ranking that the evader deems acceptable,* b∈N *is a budget specifying the maximum number of edges that can be added,* ∇ *is the set of confederates to be added to the network, and* $\hat{S}\subseteq V$ *is the set of nodes that the evader can connect to the confederates. The goal is then to identify a set* $A^{*}\subseteq \left(\nabla \times \nabla \right)\cup \left(\nabla \times \hat{S}\right)$ *such that* $|A^{*}|\leq b$*,* ${\left(V\cup \nabla ,E\cup A^{*}\right)}^{I}$ *is connected and:*

$$\#\left(v^{†},\left(V\cup \nabla ,E\cup A\right),\sigma ,I\cup \nabla \right)\geq \omega .$$

*If the algorithm* σ *is non-deterministic, then we require the above condition to be met for every possible realization of the algorithm.*

We also consider an alternative way in which the evader may conceal their true nature as the source of the diffusion. Instead of adding confederates to the network, the evader can modify (i.e., add or remove) the network edges after the diffusion has taken place. In this case, the problem faced by the evader can be defined as follows:

#### Definition 2 (hiding source by modifying edges)

*An instance of the problem is defined by a tuple,*$\left(G,v^{†},I,\sigma ,\omega ,b,\hat{A},\hat{R}\right)$, *where*G=(V,E)*is a network,*$v^{†}\in V$*is the evader,*I⊆V*is the set of infected nodes,*σ*is a source detection algorithm,*ω∈N*is a safety threshold specifying the smallest ranking that the evader deems acceptable,*b∈N*is a budget specifying the maximum number of edges that can be added or removed,*$\hat{A}\subseteq \bar{E}$*is the set of edges that can be added, and*$\hat{R}\subseteq E$*is the set of edges that can be removed. The goal is then to identify two sets,*$A^{*}\subseteq \hat{A}$*and*$R^{*}\subseteq \hat{R}$*, such that*$|A^{*}|+|R^{*}|\leq b$, ${\left(V,\left(E\cup A^{*}\right)\backslash R^{*}\right)}^{I}$*is connected and:*

$$\#\left(v^{†},\left(V,\left(E\cup A\right)\backslash R\right),\sigma ,I\right)\geq \omega .$$

*If the algorithm* σ *is non-deterministic, then we require the above condition to be met for every possible realization of the algorithm.*

#### Real-life networks datasets

We consider the following real-life networks:

• **St Lucia** (Thomas et al., 2016)—colocation network collected at the St Lucia campus of the University of Queensland using the WiFi network. The network consists of 302 nodes and 1,149 edges. Nodes represent students, while an edge between any two students indicates that they were in the same location at the same time.
• **Facebook** (Leskovec and Mcauley, 2012)—fragment of the Facebook network, an ego-network of a student of an American university. The network consists of 333 nodes and 2,523 edges. Nodes represent Facebook users, while an edge between any two users indicates that they are friends on Facebook.
• **Copenhagen** (Ahmed et al., 2010)—retweet network of the tweets regarding the United Nations Climate Change conference held in Copenhagen in December 2009. The network consists of 761 nodes and 1,029 edges. Nodes represent Twitter users, while an edge between two users indicates that at least one of them responded to a tweet by the other.
• **Gnutella** (Ripeanu et al., 2002)—Gnutella peer-to-peer file sharing network snapshot from August 8, 2002. The network consists of 6,299 nodes and 20,776 edges. Nodes represent hosts, while an edge between two nodes indicates that the two hosts exchanged a file.
• **PGP** (Boguná et al., 2004)—the network of users of the Pretty-Good-Privacy (PGP) algorithm for secure information exchange from 2004. The network consists of 10,680 nodes and 24,316 edges. Nodes represent users of the algorithm, while an edge between two nodes indicates that they mutually signed their public keys within the protocol (i.e., they formed a trust relationship).
• **Prostitution** (Rocha et al., 2010)—the giant component of a sexual contacts network between escorts and customers from a Brazilian online community. The network consists of 15,810 nodes and 38,540 edges. A node represents either an escort or a customers, while an edge between an escort and a customer indicates that the former reported a sexual contact with the latter.

The experimental procedure for the first three (smaller) real-life networks remains the same as for random networks with 1,000 nodes, whereas the procedure for the last three (larger) real-life networks is the same as for random networks with 100,000 nodes.

#### Real-life cascades dataset

We use the Twitter dataset created by Mønsted et al. (2017). More specifically, the authors created 39 new Twitter accounts and gathered some followers. Then, using these accounts, the authors started spreading eight hashtags that previously did not exist on Twitter, namely *#getyourflushot*, *#highfiveastranger*, *#somethinggood*, *#HowManyPushups*, *#turkeyface*, *#SFThanks*, *#blackfridaystories*, and *#BanksySF*. All tweets and retweets containing the new hashtags were then recorded, thereby capturing eight realistic information cascades which are included in the dataset.

Using the above dataset, we construct a network whereby a node corresponds to a Twitter account, and an edge between two nodes indicates that one is a follower or a friend of the other, or that one retweeted content posted the other. The resulting network consists of 241,698 nodes and 366,539 edges. For each hashtag listed above, we consider the account that first tweeted it as the source, and all accounts that tweeted or retweeted it at least once as the set of infected nodes. We then attempt to hide the source from source detection algorithms, using the heuristics described in the main article. We note that the Monte Carlo and random walk source detection algorithms are not included in this analysis, since they both require information about the diffusion model used to generate the set of infected nodes.

#### Alternative **diffusion** and network models

While we focus mainly on a single diffusion model, namely SI, and on three network generation models, namely Barabási-Albert, Erdős-Rényi, and Watts-Strogatz, we also consider the following three alternative models:

• Goel et al. (2016)—Goel et al. attempted to create a formal model of the spreading of viral events on Twitter. After evaluating several different models, they came to the conclusion that the one that produces results closest to reality runs the Susceptible-Infected-Recovered (SIR) diffusion in the Newman configuration networks.

The SIR diffusion model Kermack and McKendrick (1927) is similar to the SI model used in the remainder of our work, but an infected node can move to the Recovered state, after which it cannot infect others nor become infected again. Goel et al. use a variation of the SIR model where an infected node moves to the Recovered state one round after it gets infected, and where the probability of infection is equal to $\frac{1}{2d^{*}}$, with $d^{*}$ being the average degree in the network. The authors also assume that, after the diffusion process terminates, the information about whether a node was infected or not is available to the party analyzing the network (i.e., it is possible to discern between the nodes that are in the Susceptible state and those that are in the Recovered state). In our simulations we use the exact same parameterization of this diffusion model.

As for the Newman configuration model Newman (2003), it is a model that generates networks with a given degree distribution. Goal et al. used networks with degrees drawn from the power law distribution $P\left(d\right)∼d^{-\alpha }$, where α=2.3. We use the same model of generating networks in our simulations.

• Kleinberg (2007)—Kleinberg’s model of diffusion is based on the assumption that in order for a node to adopt a certain phenomenon, a given number of the node’s neighbors must adopt it first. Before the diffusion starts, Kleinberg randomly generates for every node v a threshold $\theta_{v}\in \left[0,1\right]$ from a uniform distribution. At the beginning of the diffusion only the source node is active, while the rest of the nodes are inactive. A given inactive node becomes active when the proportion of its active neighbors becomes greater than $\theta_{v}$, i.e., when:

$$\frac{\left|\left\{w\in N\left(v\right):w\in I\right\}\right|}{d\left(v\right)}>\theta_{v}$$

where I is the set of active nodes. The diffusion ends when no new nodes can become active. We run the simulations for this diffusion model in the same networks as for Goel et al. (2016).

• Golub and Jackson (2012)—Golub and Jackson created the “islands” model that generates networks with a strong community structure. The set of nodes is divided into equally-sized groups. Every node forms a connection with another node from the same group with probability $p_{same}$, and with any other nodes from a different group with probability $p_{diff}\leq p_{same}$. In our simulations we consider the islands networks with 10 groups of size 100 each, and with the average degree 4, where each node is on average connected with 3 others from the same group and with 1 node from a different group. With these parameters, we obtain networks of the same size and average degree as those used in the experiments presented in the main article. As for the diffusion model, we use the SI model.

#### **Alternative** experimental settings

We consider the following three alternative simulation settings:

• **Effects of hiding the nearby nodes**—we investigate how the evader’s ranking is affected when another node in the evader’s direct network vicinity runs the hiding process. To this end, we run the basic version of our experiments but this time it is not the evader who runs the heuristic, but rather one of the infected nodes at distance 5 or less from the evader. Here, we evaluate the two heuristics that proved to be the most effective in our basic experiments, i.e., one that adds nodes and one that modifies edges.
• **The seeker with imperfect knowledge**—we investigate how the completeness of the seeker’s knowledge about the network’s structure affects the effectiveness of the evader’s hiding. To this end, we run the simulations where the seeker knows only about a certain percentage of the network’s edges, which are selected uniformly at random at the beginning of each experiment. Then, we evaluate the two heuristics that proved to be the most effective in our basic experiments, i.e., one that adds nodes and one that modifies edges.
• **Randomly selected evader**—instead of selecting the evader randomly from the 10% of nodes with the highest degrees, we select the evader uniformly at random from all nodes in the network. All other parameters of the simulations remain exactly the same as in the basic experiments presented elsewhere in our study.

### Quantification and statistical analysis

All results for random networks (Figures 2, 3, 4, and 5) are reported as a mean over 100 networks generated using each model, and 10 different evaders in each network. The results for real networks (Figure 6) are reported as a mean over 100 different evaders in each network. All confidence intervals are computed for 95% confidence level.

### Additional resources

This work does not include any additional resources.

## Data Availability

This paper analyzes existing, publicly available data. The access links are listed in the Key resources table. All original code is available online. The access link is listed in the Key resources table. Any additional information required to reanalyze the data reported in this paper is available from the lead contact upon request.

## References

[bib1] Ahmed N.K., Berchmans F., Neville J., Kompella R. (2010). SIGKDD MLG.

[bib2] Antulov-Fantulin N., Lančić A., Šmuc T., Štefančić H., Šikić M. (2015). Identification of patient zero in static and temporal networks: robustness and limitations. Phys. Rev. Lett..

[bib3] Arora S., Barak B. (2009).

[bib4] Barabási A.L. (2016).

[bib5] Barabási A.L., Albert R. (1999). Emergence of scaling in random networks. Science.

[bib6] Barrat A., Barthelemy M., Vespignani A. (2008).

[bib7] Block P., Hoffman M., Raabe I.J., Dowd J.B., Rahal C., Kashyap R., Mills M.C. (2020). Social network-based distancing strategies to flatten the COVID-19 curve in a post-lockdown world. Nat. Hum. Behav..

[bib8] Boguñá M., Pastor-Satorras R., Díaz-Guilera A., Arenas A. (2004). Models of social networks based on social distance attachment. Phys. Rev. E Stat. Nonlin. Soft Matter Phys..

[bib9] Bovet A., Makse H.A. (2019). Influence of fake news in twitter during the 2016 us presidential election. Nat. Commun..

[bib10] Braunstein A., Ingrosso A., Muntoni A.P. (2019). Network reconstruction from infection cascades. J. R. Soc. Interface.

[bib11] Cai K., Xie H., Lui J.C.S. (2018). Information spreading forensics via sequential dependent snapshots. IEEE/ACM Trans. Netw..

[bib12] Chiu W.A., Fischer R., Ndeffo-Mbah M.L. (2020). State-level needs for social distancing and contact tracing to contain COVID-19 in the United States. Nat. Hum. Behav..

[bib13] Chwistek K., Butail S. (2020). 2020 American Control Conference (ACC).

[bib14] Cogan P., Andrews M., Bradonjic M., Kennedy W.S., Sala A., Tucci G. (2012). Proceedings of the First ACM International Workshop on Hot Topics on Interdisciplinary Social Networks Research.

[bib15] Comin C.H., Costa L.d.F. (2011). Identifying the starting point of a spreading process in complex networks. Phys. Rev. E Stat. Nonlin. Soft Matter Phys..

[bib16] De Domenico M., Lima A., Mougel P., Musolesi M. (2013). The anatomy of a scientific rumor. Sci. Rep..

[bib17] Douceur J.R. (2002). International workshop on peer-to-peer systems.

[bib18] Erdős P., Rényi A. (1959). On random graphs i. Publ. Math. Debrecen.

[bib19] Farajtabar M., Rodriguez M.G., Zamani M., Du N., Zha H., Song L. (2015). Artificial Intelligence and Statistics.

[bib20] Ghosh R., Lerman K. (2011). Proceedings of the fourth ACM international conference on Web search and data mining.

[bib21] Goel S., Anderson A., Hofman J., Watts D.J. (2016). The structural virality of online diffusion. Manage. Sci..

[bib22] Goffman W., Newill V.A. (1964). Generalization of epidemic theory: an application to the transmission of ideas. Nature.

[bib23] Golub B., Jackson M.O. (2012). Does homophily predict consensus times? testing a model of network structure via a dynamic process. Rev. Netw. Econ..

[bib24] Gomez Rodriguez M., Leskovec J., Schölkopf B. (2013). Proceedings of the sixth ACM international conference on Web search and data mining.

[bib25] Jain A., Borkar V., Garg D. (2016). Fast rumor source identification via random walks. Soc. Netw. Anal. Min..

[bib26] Kermack W.O., McKendrick A.G. (1927). A contribution to the mathematical theory of epidemics. Proc. R. Soc. A.

[bib27] Kleinberg J. (2007). Cascading behavior in networks: algorithmic and economic issues. Algorithmic Game Theory.

[bib28] Lehmann S., Ahn Y.Y. (2018).

[bib29] Leskovec J., Mcauley J.J. (2012). Advances in neural information processing systems.

[bib30] Li X., Li X. (2017). Reconstruction of stochastic temporal networks through diffusive arrival times. Nat. Commun..

[bib31] Lokhov A.Y., Mézard M., Ohta H., Zdeborová L. (2013). Inferring the origin of an epidemic with dynamic message-passing algorithm. Phys. Rev. E.

[bib32] Mønsted B., Sapieżyński P., Ferrara E., Lehmann S. (2017). Evidence of complex contagion of information in social media: an experiment using twitter bots. PLoS One.

[bib33] Newman M.E.J. (2003). The structure and function of complex networks. SIAM Rev. Soc. Ind. Appl. Math..

[bib34] Pajevic S., Plenz D. (2009). Efficient network reconstruction from dynamical cascades identifies small-world topology of neuronal avalanches. PLoS Comput. Biol..

[bib35] Paluch R., Lu X., Suchecki K., Szymański B.K., Hołyst J.A. (2018). Fast and accurate detection of spread source in large complex networks. Sci. Rep..

[bib36] Pinto P.C., Thiran P., Vetterli M. (2012). Locating the source of diffusion in large-scale networks. Phys. Rev. Lett..

[bib37] Matei R., Iamnitchi A., Foster P. (2002). Mapping the gnutella network. IEEE Internet Comput..

[bib38] Rocha L.E.C., Liljeros F., Holme P. (2010). Information dynamics shape the sexual networks of internet-mediated prostitution. Proc. Natl. Acad. Sci. USA.

[bib39] Rozenshtein P., Gionis A., Prakash B.A., Vreeken J. (2016). Proceedings of the 22nd ACM SIGKDD International Conference on Knowledge Discovery and Data Mining.

[bib40] Sadikov E., Medina M., Leskovec J., Garcia-Molina H. (2011). Proceedings of the fourth ACM international conference on Web search and data mining.

[bib41] Shah C., Dehmamy N., Perra N., Chinazzi M., Barabási A.L., Vespignani A., Yu R. (2020). Finding patient zero: learning contagion source with graph neural networks. arXiv.

[bib42] Shah D., Zaman T. (2011). Rumors in a network: who’s the culprit?. IEEE Trans. Inf. Theory.

[bib43] Shelke S., Attar V. (2019). Source detection of rumor in social network: a review. Online Soc. Netw. Media.

[bib44] Shen Z., Wang W.X., Fan Y., Di Z., Lai Y.C. (2014). Reconstructing propagation networks with natural diversity and identifying hidden sources. Nat. Commun..

[bib45] Sundareisan S., Vreeken J., Prakash B.A. (2015). Proceedings of the 2015 SIAM International Conference on Data Mining.

[bib46] Taxidou I., Fischer P.M. (2014). Proceedings of the 23rd International Conference on World Wide Web.

[bib47] Thomas B., Jurdak R., Zhao K., Atkinson I. (2016). Diffusion in colocation contact networks: the impact of nodal spatiotemporal dynamics. PLoS One.

[bib48] Wang Z., Dong W., Zhang W., Tan C.W. (2014). Rumor source detection with multiple observations: fundamental limits and algorithms. SIGMETRICS Perform. Eval. Rev..

[bib49] Wang Z., Dong W., Zhang W., Tan C.W. (2015). Rooting our rumor sources in online social networks: the value of diversity from multiple observations. IEEE J. Sel. Top. Signal Process..

[bib50] Waniek M., Cebrian M., Holme P., Rahwan T. (2021). https://arxiv.org/abs/2102.10539.

[bib51] Waniek M., Michalak T.P., Wooldridge M., Rahwan T. (2021). How members of covert networks conceal the identities of their leaders. ACM Trans. Intell. Syst. Technol..

[bib52] Waniek M., Holme P., Rahwan T. (2022). Hiding in temporal networks. IEEE Trans. Netw. Sci. Eng..

[bib53] Waniek M., Michalak T., Rahwan T. (2020). Proceedings of the AAAI Conference on Artificial Intelligence.

[bib54] Waniek M., Michalak T.P., Rahwan T., Wooldridge M. (2017). Proceedings of the 16th Conference on Autonomous Agents and MultiAgent Systems.

[bib55] Waniek M., Michalak T.P., Wooldridge M.J., Rahwan T. (2018). Hiding individuals and communities in a social network. Nat. Hum. Behav..

[bib56] Waniek M., Zhou K., Vorobeychik Y., Moro E., Michalak T.P., Rahwan T. (2019). How to hide one’s relationships from link prediction algorithms. Sci. Rep..

[bib57] Was T., Waniek M., Rahwan T., Michalak T. (2020). Proceedings of the 19th International Conference on Autonomous Agents and MultiAgent Systems.

[bib58] Watts D.J., Strogatz S.H. (1998). Collective dynamics of ‘small-world’ networks. nature.

[bib59] Xiao H., Aslay C., Gionis A. (2018). 2018 IEEE International Conference on Data Mining (ICDM).

[bib60] Xiao H., Rozenshtein P., Tatti N., Gionis A. (2018). Proceedings of the 2018 SIAM International Conference on Data Mining.

[bib61] Xu W., Chen H. (2015). 2015 11th International Conference on Mobile Ad-hoc and Sensor Networks.

[bib62] Zhou K., Michalak T.P., Waniek M., Rahwan T., Vorobeychik Y. (2019). Proceedings of the 18th International Conference on Autonomous Agents and Multi-Agent Systems.

