# Supplementary material for: Social diffusion sources can escape detection
Source: iScience. 2022 Aug 19;25(9):104956. doi: 10.1016/j.isci.2022.104956 (PMC9459693; doi:10.1016/j.isci.2022.104956)
Supplement: Document S1. Figures S1–S57 [file mmc1.pdf]

iScience, Volume *25*

Supplemental information

Social diffusion sources can escape detection

Marcin Waniek, Petter Holme, Manuel Cebrian, and Talal Rahwan

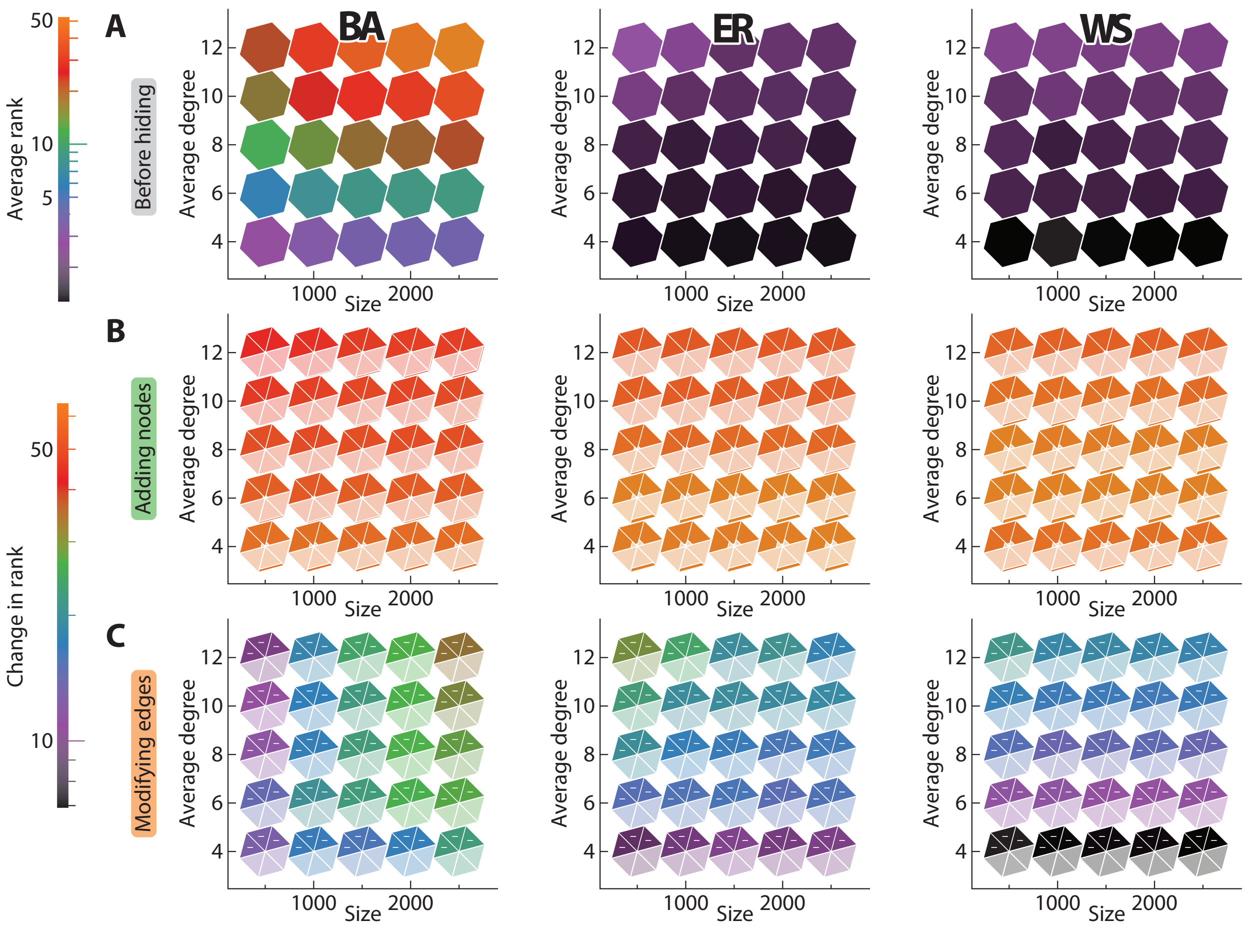

Figure S1: **The efficiency of hiding from the Degree source detection algorithm in networks with varying structure, size and density; related to Figure 2.** The same as Figure 2 in the main article, but for Degree source detection algorithm instead of Eigenvector.

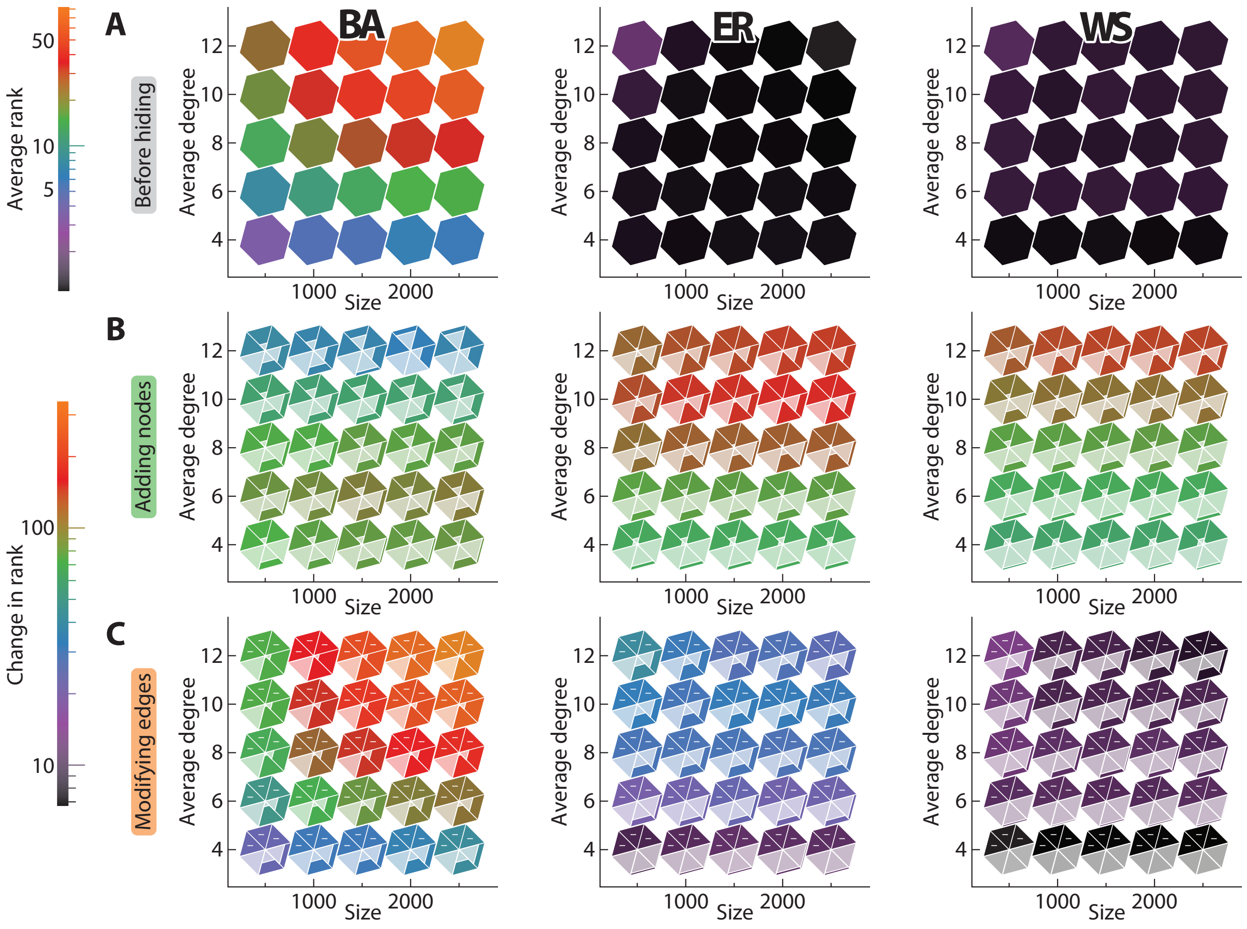

Figure S2: **The efficiency of hiding from the Closeness source detection algorithm in networks with varying structure, size and density; related to Figure 2.** The same as Figure 2 in the main article, but for Closeness source detection algorithm instead of Eigenvector.

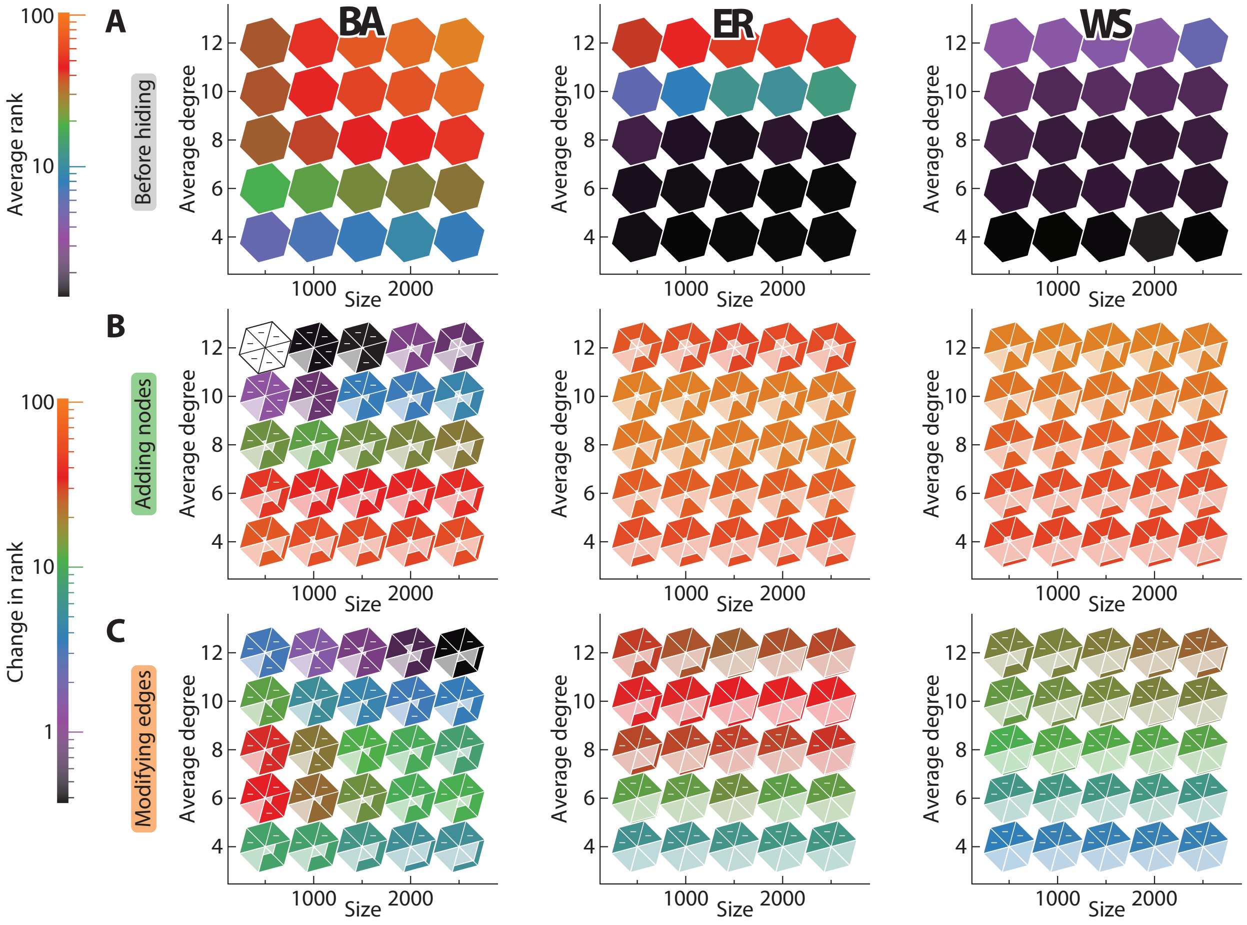

Figure S3: **The efficiency of hiding from the Rumor source detection algorithm in networks with varying structure, size and density, related to Figure 2.** The same as Figure 2 in the main article, but for Rumor source detection algorithm instead of Eigenvector. White hexagons imply that all hiding strategies increase the visibility of the evader.

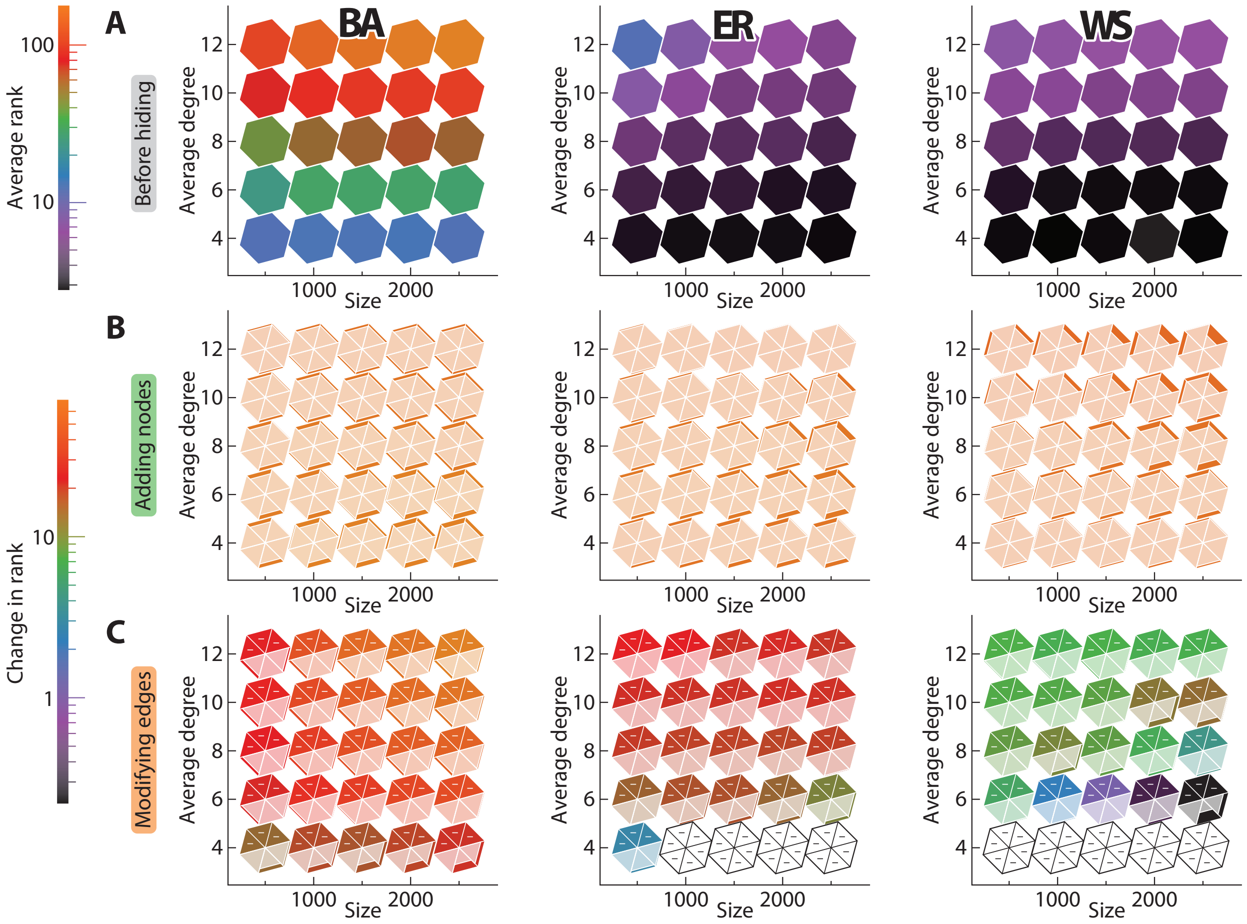

Figure S4: **The efficiency of hiding from the Random walk source detection algorithm in networks with varying structure, size and density, related to Figure 2.** The same as Figure 2 in the main article, but for Random walk source detection algorithm instead of Eigenvector. White hexagons imply that all hiding strategies increase the visibility of the evader.

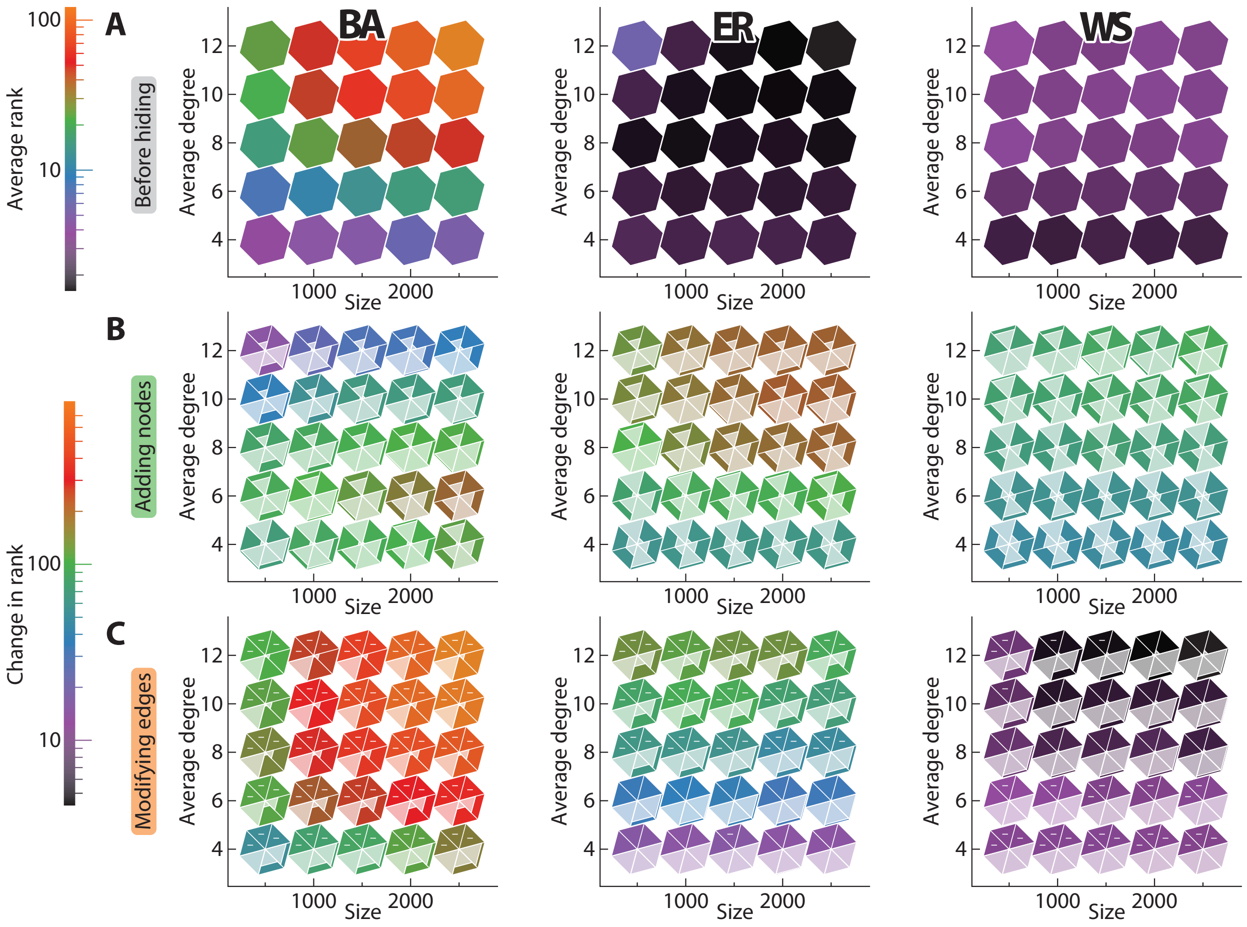

Figure S5: **The efficiency of hiding from the Monte Carlo source detection algorithm in networks with varying structure, size and density, related to Figure 2.** The same as Figure 2 in the main article, but for Monte Carlo source detection algorithm instead of Eigenvector.

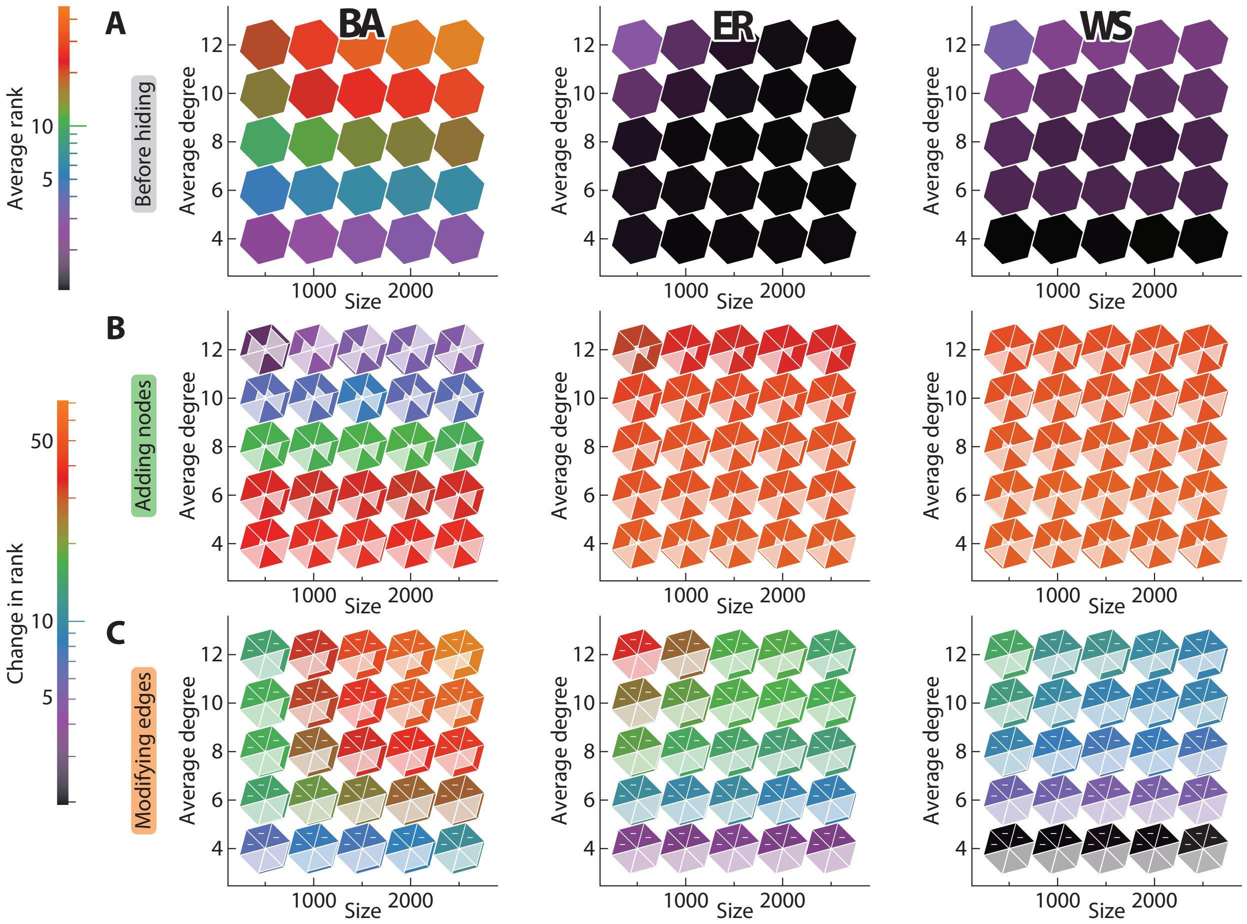

Figure S6: **The efficiency of hiding from the Betweenness source detection algorithm in networks with varying structure, size and density, related to Figure 2.** The same as Figure 2 in the main article, but for Betweenness source detection algorithm instead of Eigenvector.

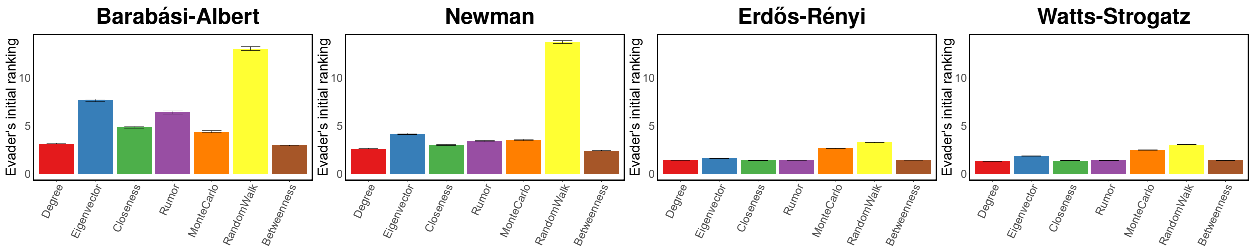

Figure S7: **Comparison of the effectiveness of different source detection algorithms before the hiding process in random networks consisting of 1,000 nodes, related to Figure 2.** The x-axis corresponds to different source detection algorithms, while the y-axis corresponds to the initial ranking of the evader. The error bars represent 95% confidence intervals.

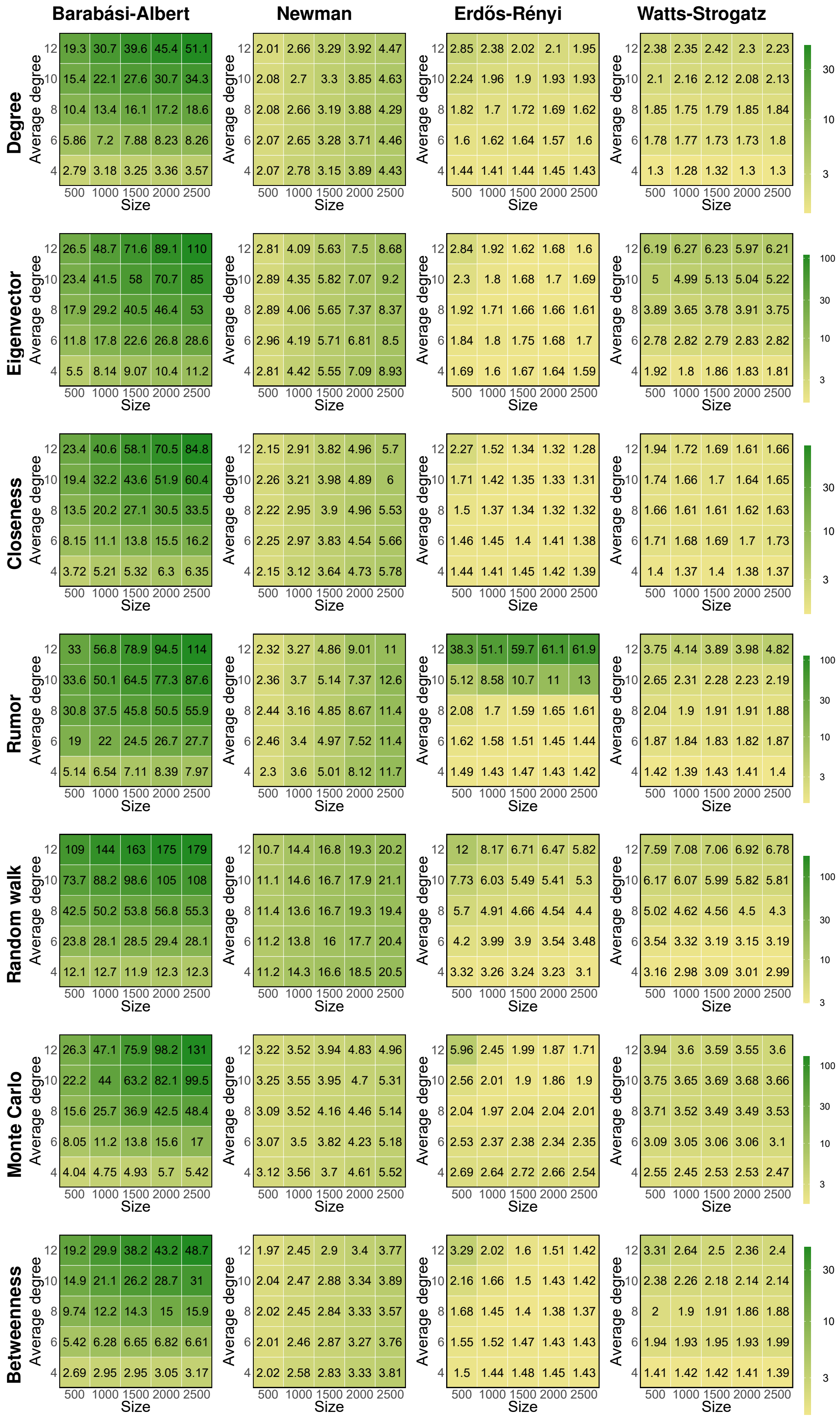

Figure S8: **Evader ranking before the hiding process, related to Figure 2.** Each row corresponds to a different source detection algorithm, while each column corresponds to a different network generation model. In each heatmap, the x-axis represents the number of nodes in the network, while the y-axis represents the average degree. The color of each cell represents the average position of the evader in the ranking computed using the source detection algorithm before the hiding process on a logarithmic scale (the lower the ranking, the more exposed is the evader). The results are presented as an average over 100 networks and over 10 evaders in each network.

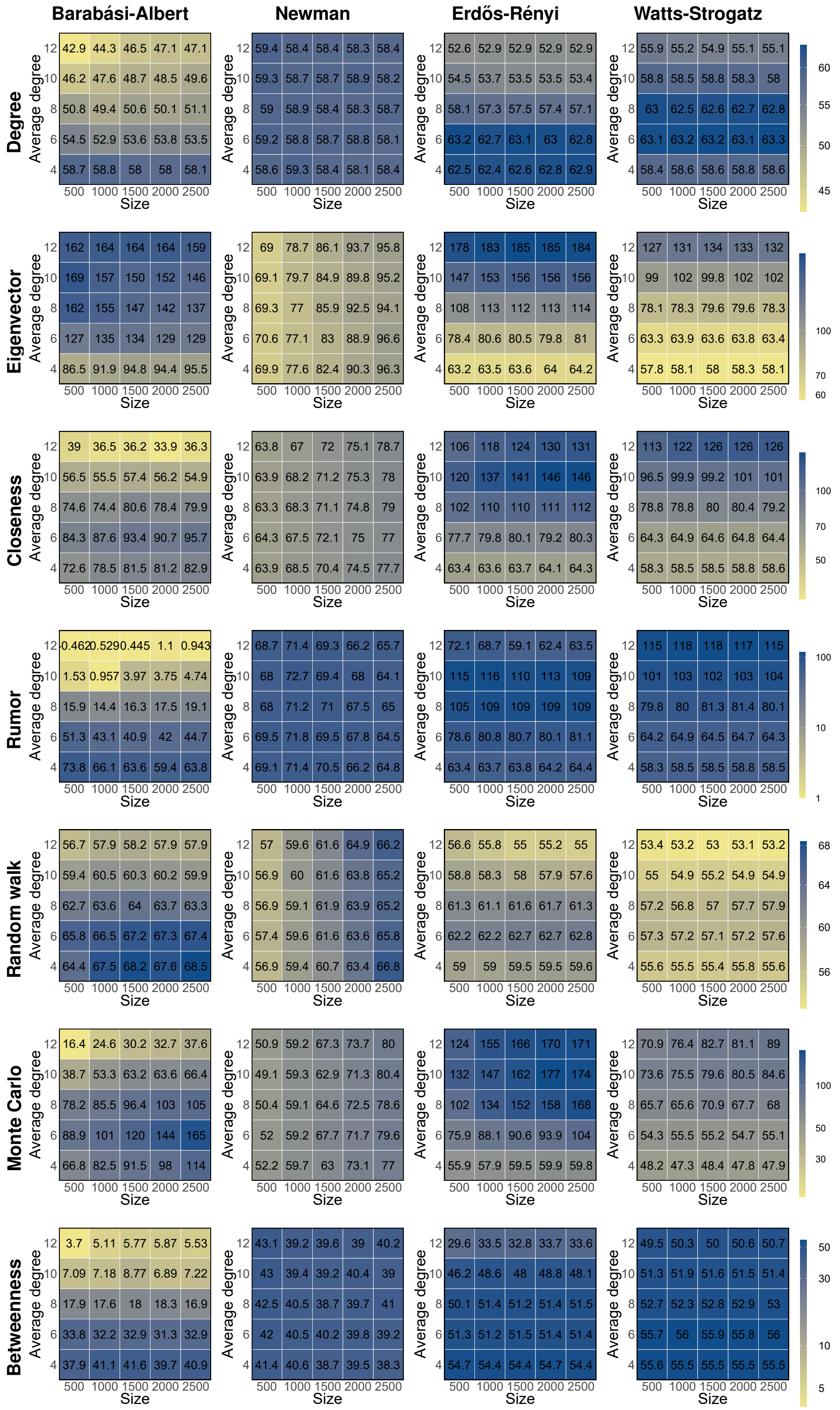

Figure S9: **The efficiency of hiding using the best considered heuristic that adds nodes, related to Figure 2.** Each row corresponds to a different source detection algorithm, while each column corresponds to a different network generation model. In each heatmap, the x-axis represents the number of nodes in the network, while the y-axis represents the average degree. The color of each cell represents the average difference in the evader's ranking as a result of the best hiding heuristic. The results are presented as an average over 100 networks and over 10 evaders in each network.

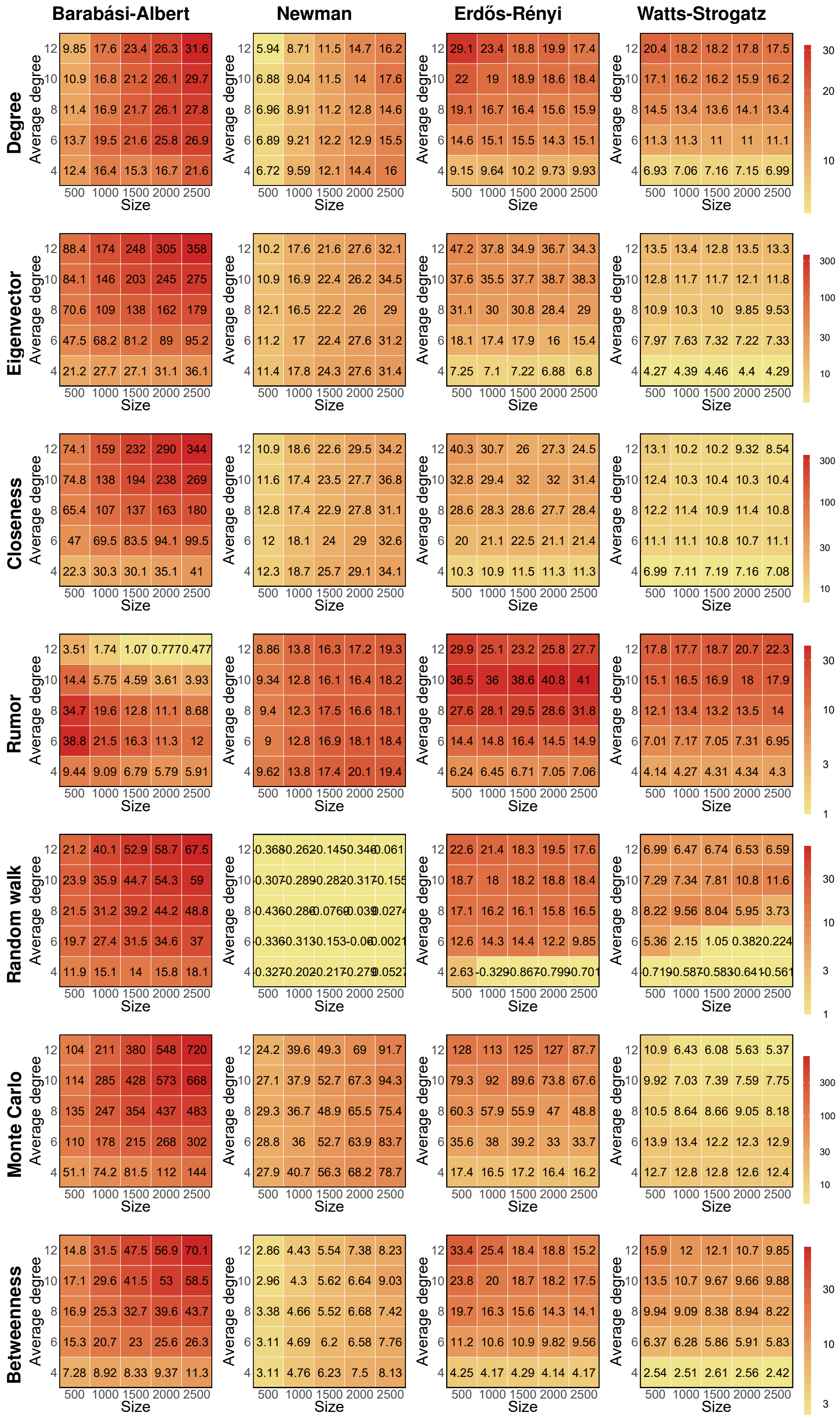

Figure S10: **The efficiency of hiding using the best considered heuristic that modifies edges, related to Figure 2.** Each row corresponds to a different source detection algorithm, while each column corresponds to a different network generation model. In each heatmap, the x-axis represents the number of nodes in the network, while the y-axis represents the average degree. The color of each cell represents the average difference in the evader's ranking as a result of the best hiding heuristic. The results are presented as an average over 100 networks and over 10 evaders in each network.

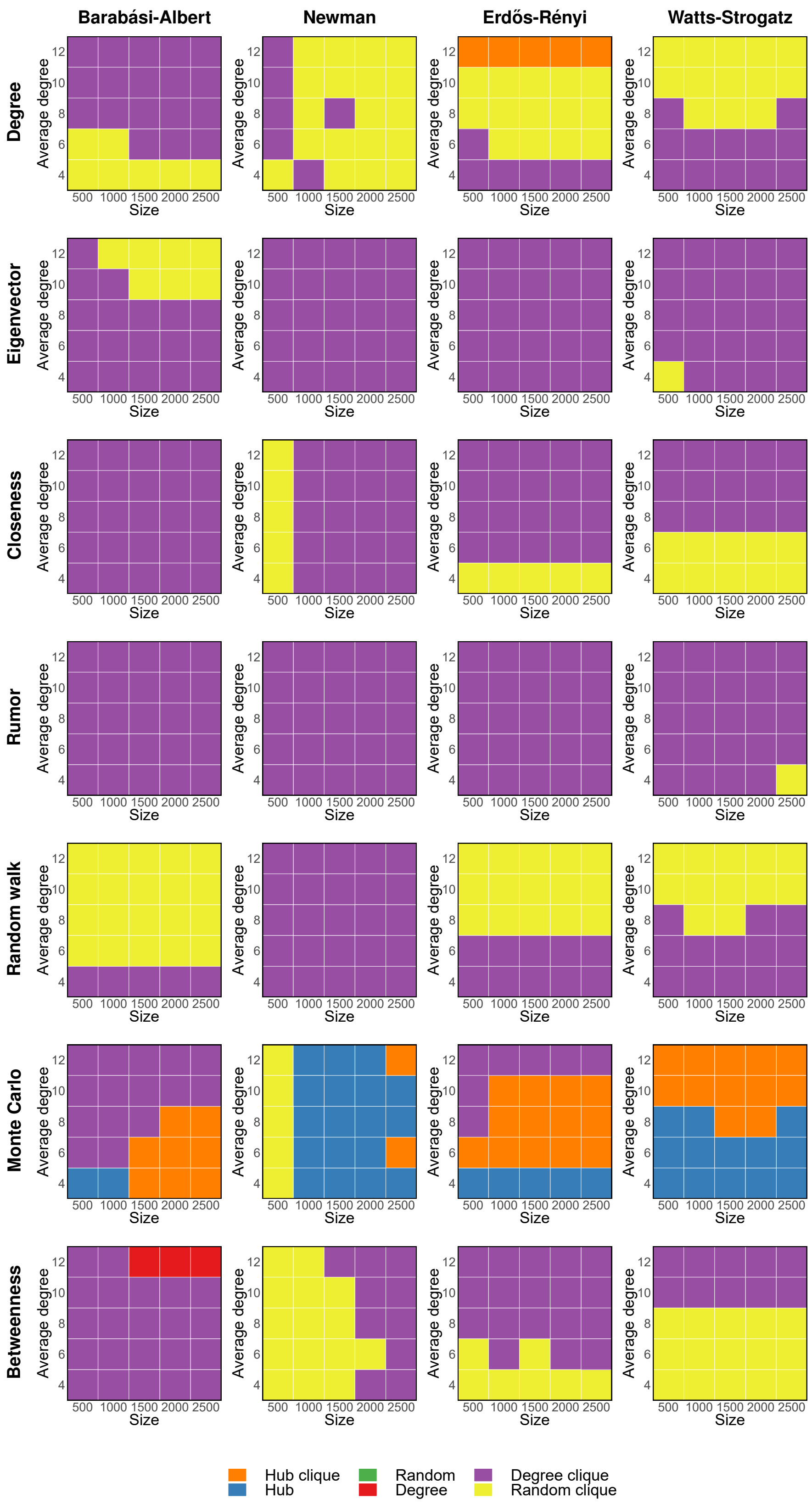

Figure S11: **The most effective heuristic that adds nodes in networks with varying structure, size and density, related to Figure 2.** Each row corresponds to a different source detection algorithm, while each column corresponds to a different network generation model. In each heatmap, the x-axis represents the number of nodes in the network, while the y-axis represents the average degree. The color of each cell represents the best considered hiding heuristic that adds nodes. The results are presented as an average over 100 networks and over 10 evaders in each network.

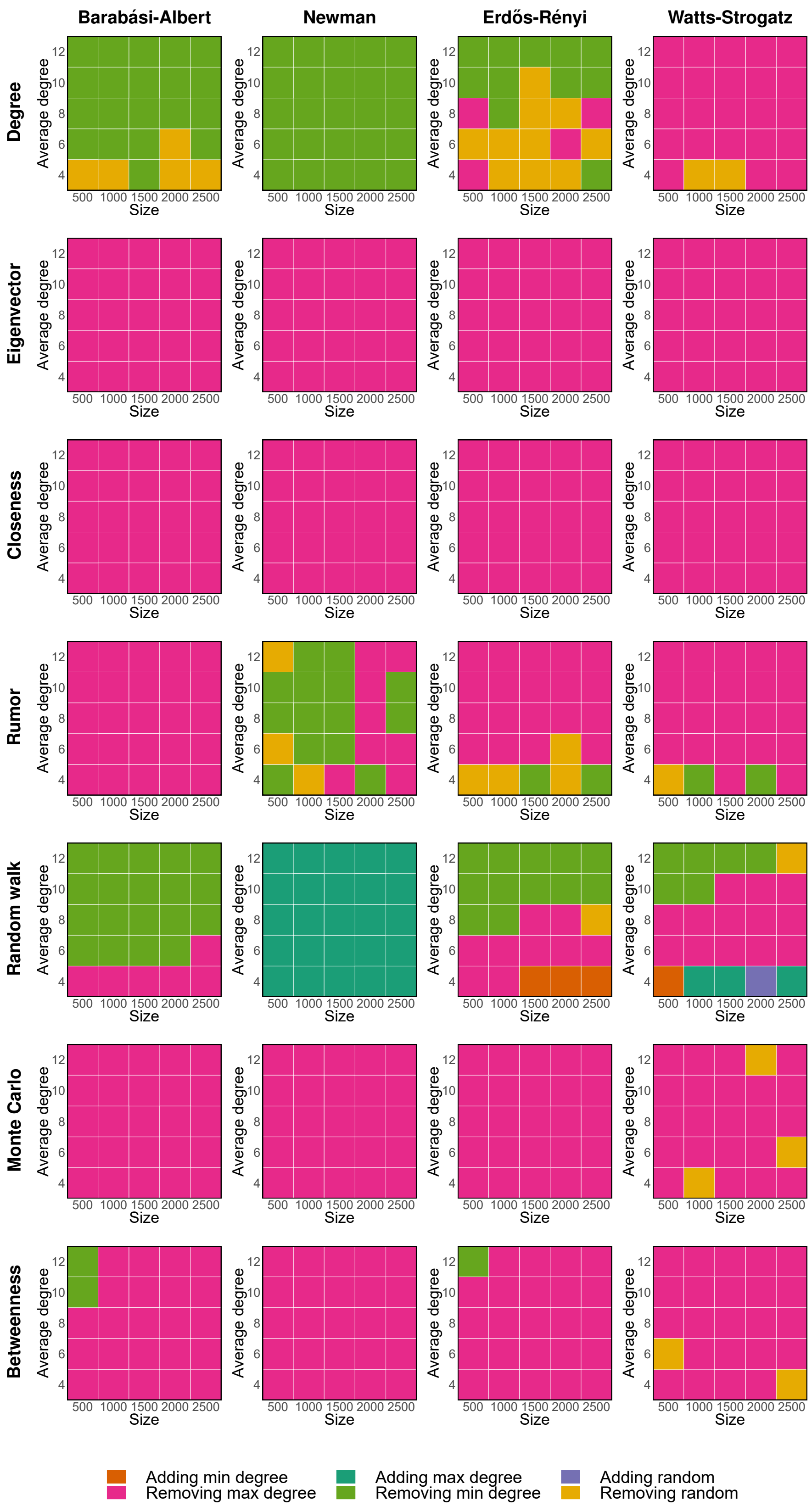

Figure S12: **The most effective heuristic that modifies edges in networks with varying structure, size and density, related to Figure 2.** Each row corresponds to a different source detection algorithm, while each column corresponds to a different network generation model. In each heatmap, the x-axis represents the number of nodes in the network, while the y-axis represents the average degree. The color of each cell represents the best considered hiding heuristic that modifies edges. The results are presented as an average over 100 networks and over 10 evaders in each network.

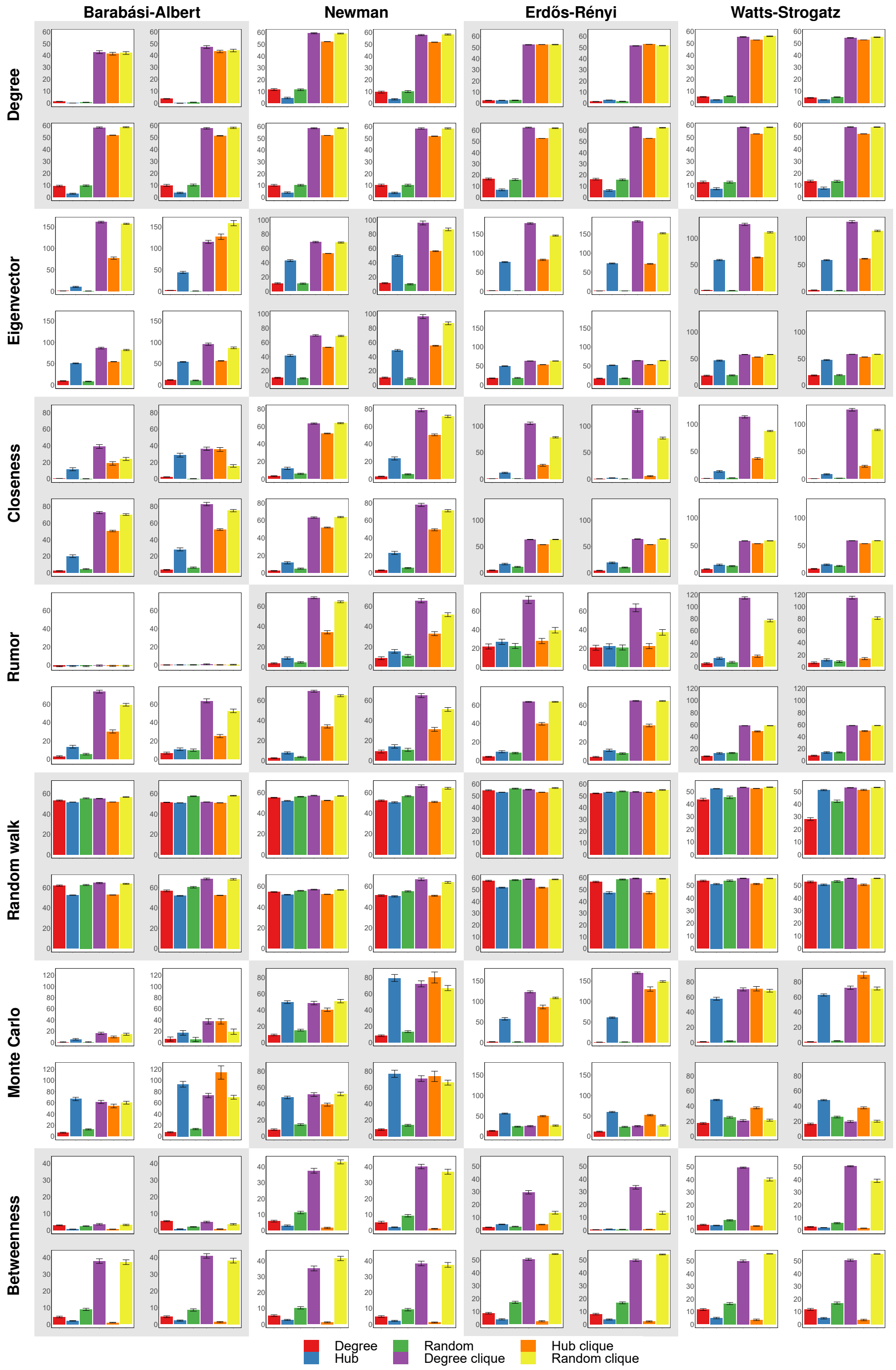

Figure S13: **The effectiveness comparison of heuristics that add nodes in networks with varying structure, size and density, related to Figure 2.** Each row corresponds to a different source detection algorithm, while each column corresponds to a different network generation model. In each group of four plots, the leftmost plots correspond to networks with 500 nodes, the rightmost to networks with 2500 nodes, the top to networks with average degree 12, and the bottom to networks with average degree 4. In each barplot, the x-axis represents different heuristics that add nodes, while the y-axis represents the effectiveness of the heuristic. The results are presented as an average over 100 networks and over 10 evaders in each network.

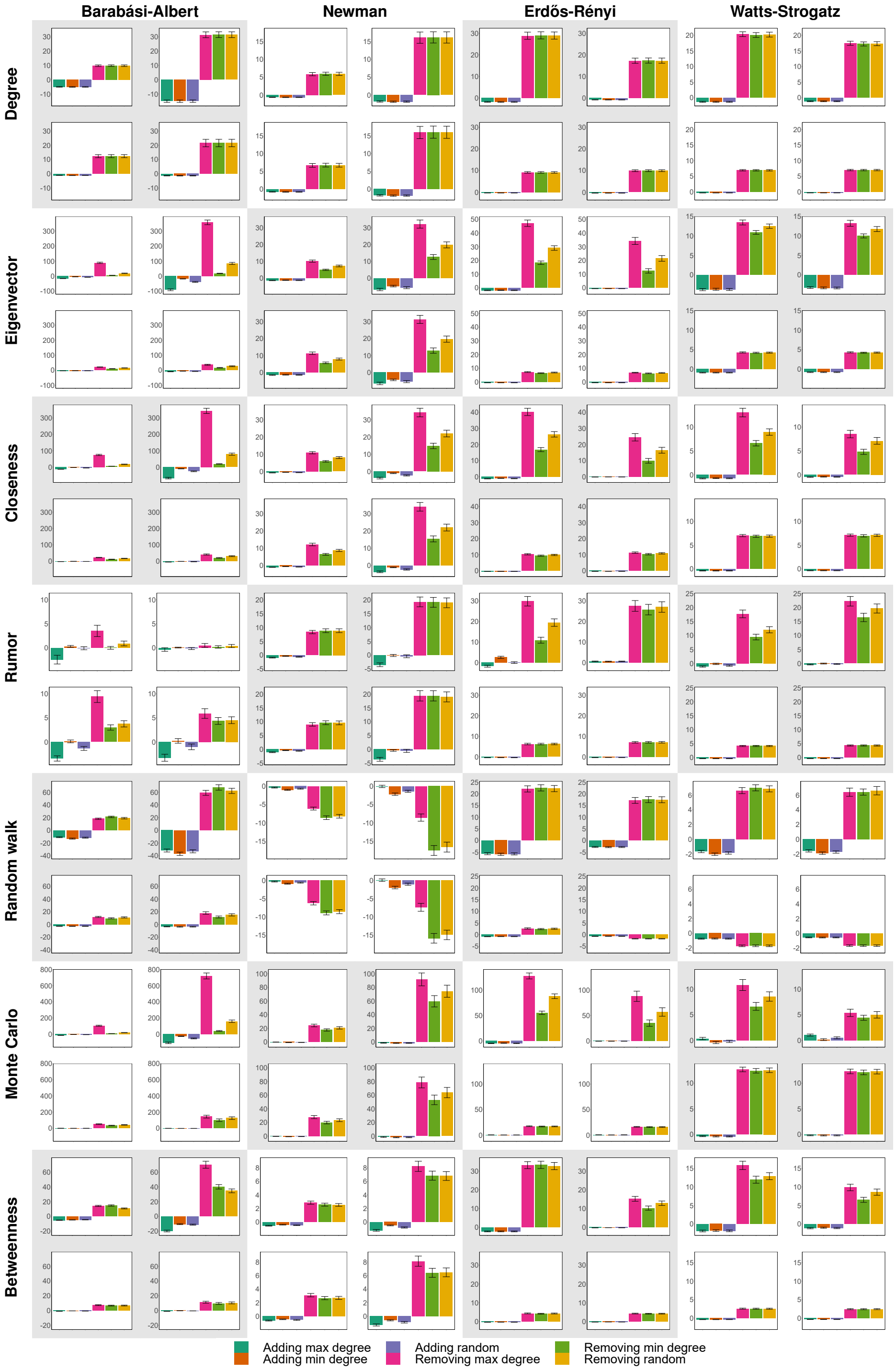

Figure S14: **The effectiveness comparison of heuristics that modify edges in networks with varying structure, size and density, related to Figure 2.** Each row corresponds to a different source detection algorithm, while each column corresponds to a different network generation model. In each group of four plots, the leftmost plots correspond to networks with 500 nodes, the rightmost to networks with 2500 nodes, the top to networks with average degree 12, and the bottom to networks with average degree 4. In each barplot, the x-axis represents different heuristics that modify edges, while the y-axis represents the effectiveness of the heuristic. The results are presented as an average over 100 networks and over 10 evaders in each network.

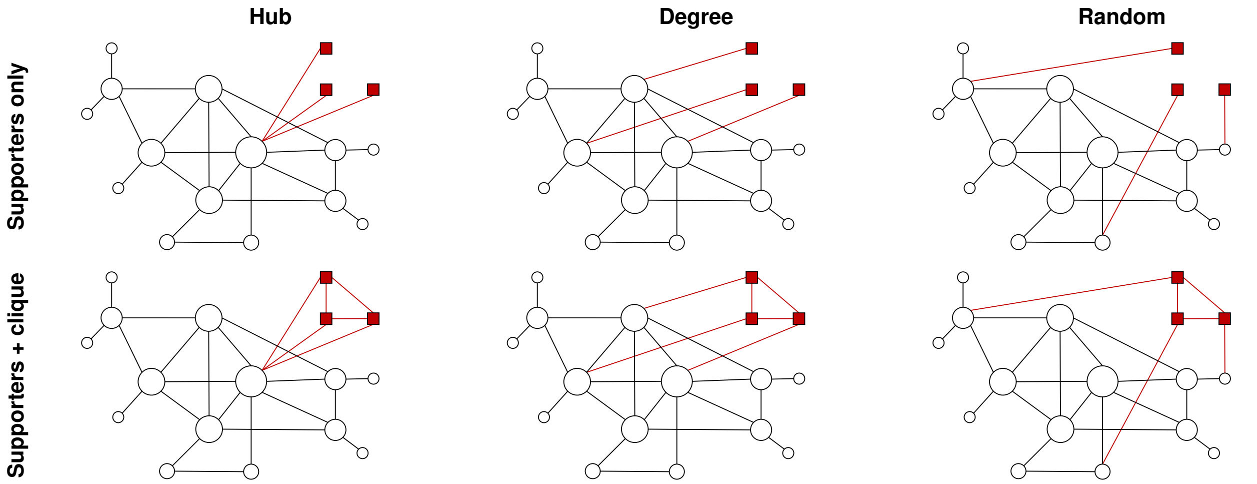

Figure S15: **Examples of heuristics that add new nodes to the network, related to Figure 2.** Red squared nodes represent confederates (new nodes), while rounded nodes represent network members. The size of each rounded node corresponds to its degree. Each column corresponds to a different way of selecting supporters to be connected with the confederates. The first row shows examples of heuristics that connects confederates with supporters only, while the second row shows examples of heuristics that additionally connect all confederates into a clique.

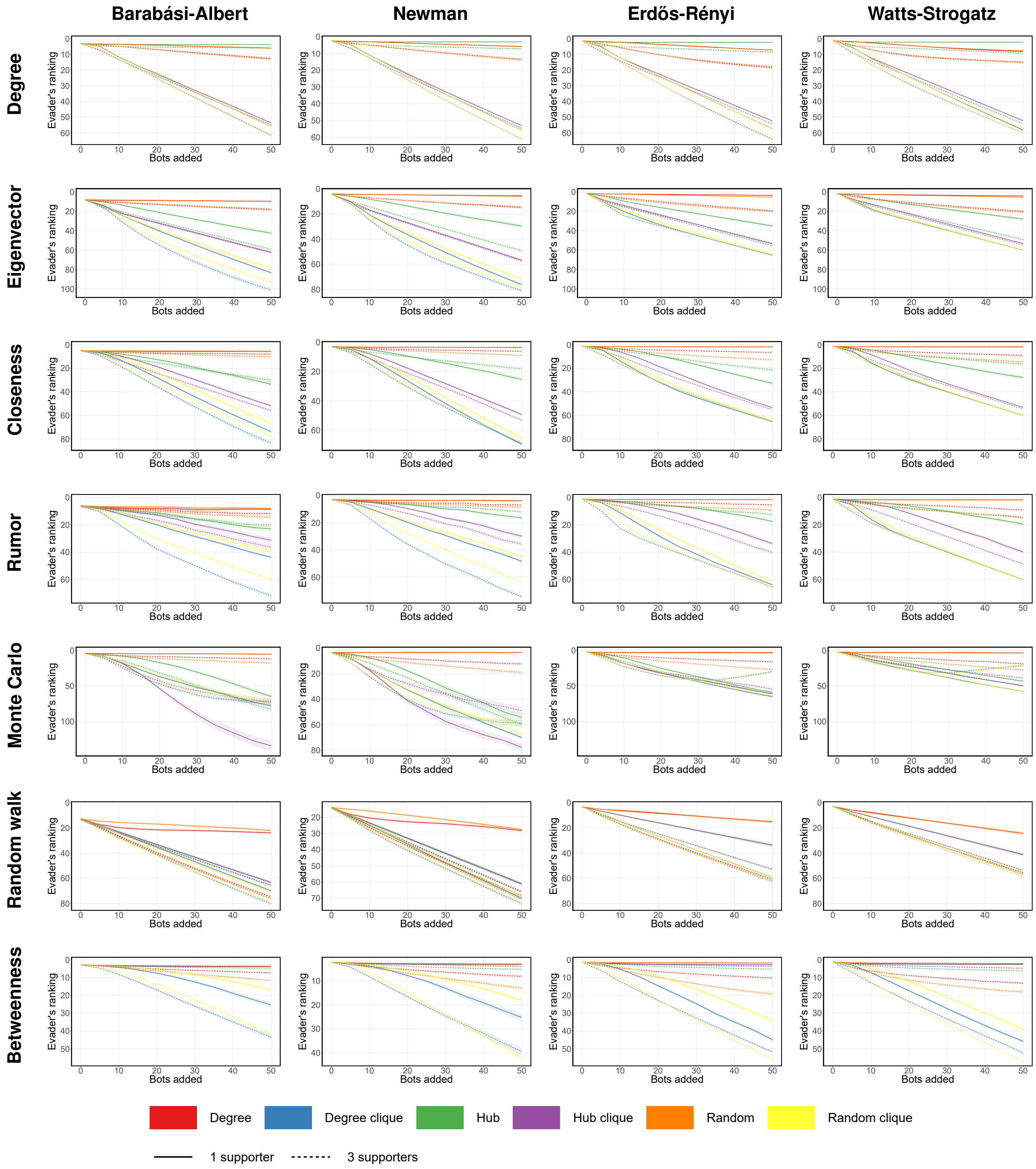

Figure S16: **Results of hiding the source of diffusion by adding nodes, related to Figure 2.** The y-axis corresponds to the ranking of the evader according to the source detection algorithm (greater values indicate more efficient hiding), while the x-axis corresponds to the number of nodes added to the network. Each color corresponds to a different heuristic. Solid lines correspond to cases where each confederate is connected to a single supporter, while dashed lines correspond to cases where each confederate is connected to three supporters. Results are presented for networks consisting of 1,000 nodes with an average degree of 4. Shaded areas represent 95% confidence intervals.

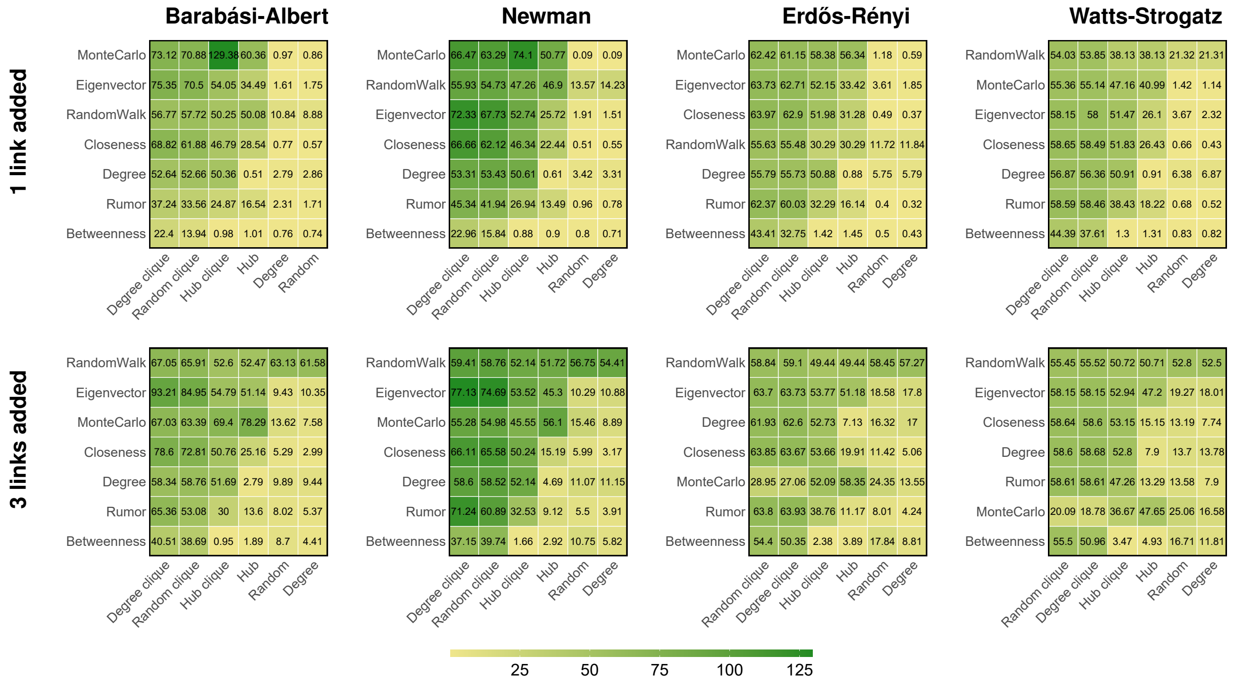

Figure S17: **Results of hiding the source of diffusion by adding nodes, related to Figure 2.** The y-axis of each heatmap corresponds to different source detection algorithms, whereas the x-axis corresponds to different heuristics. The value in each cell indicates the change in the evader's ranking according to the source detection algorithm after adding 50 confederates to the network using the heuristic. Results are presented for networks consisting of 1,000 nodes with an average degree of 4. Rows and columns are sorted by average value.

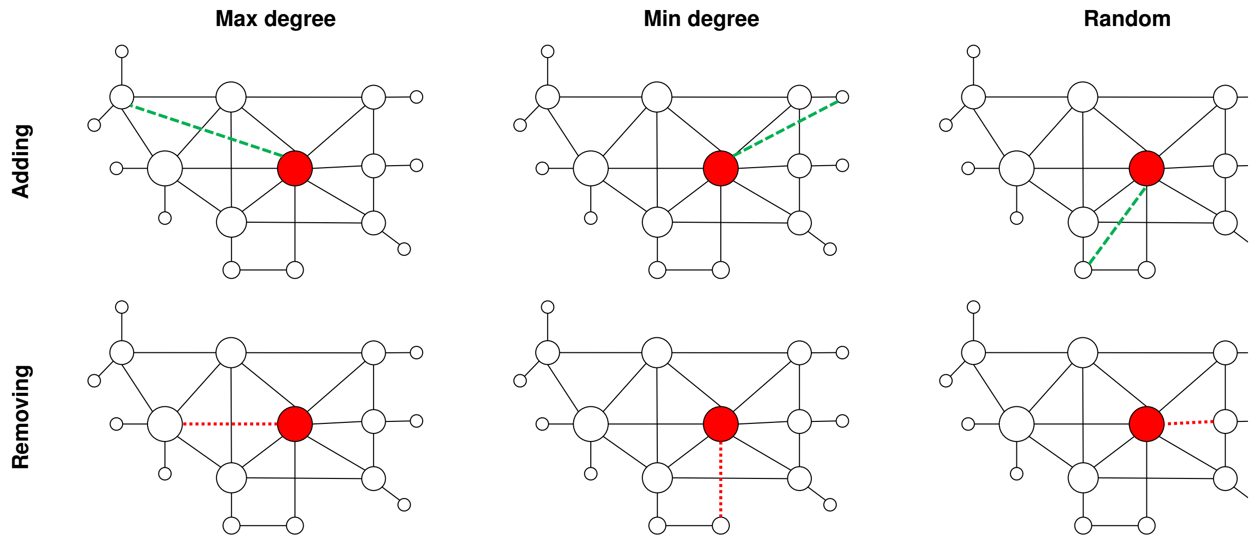

Figure S18: **Examples of heuristics that modify edges of the network, related to Figure 2.** The red node in each network represents the evader. The size of each node corresponds to its degree. Green dashed edges represent newly added connections, while dotted red edges represent newly removed connections. The first row shows examples of heuristics that add edges to the network, while the second row shows examples of heuristics that remove edges from the network. Each column corresponds to a different way of selecting the nodes that will be connected to, or disconnected from, the evader.

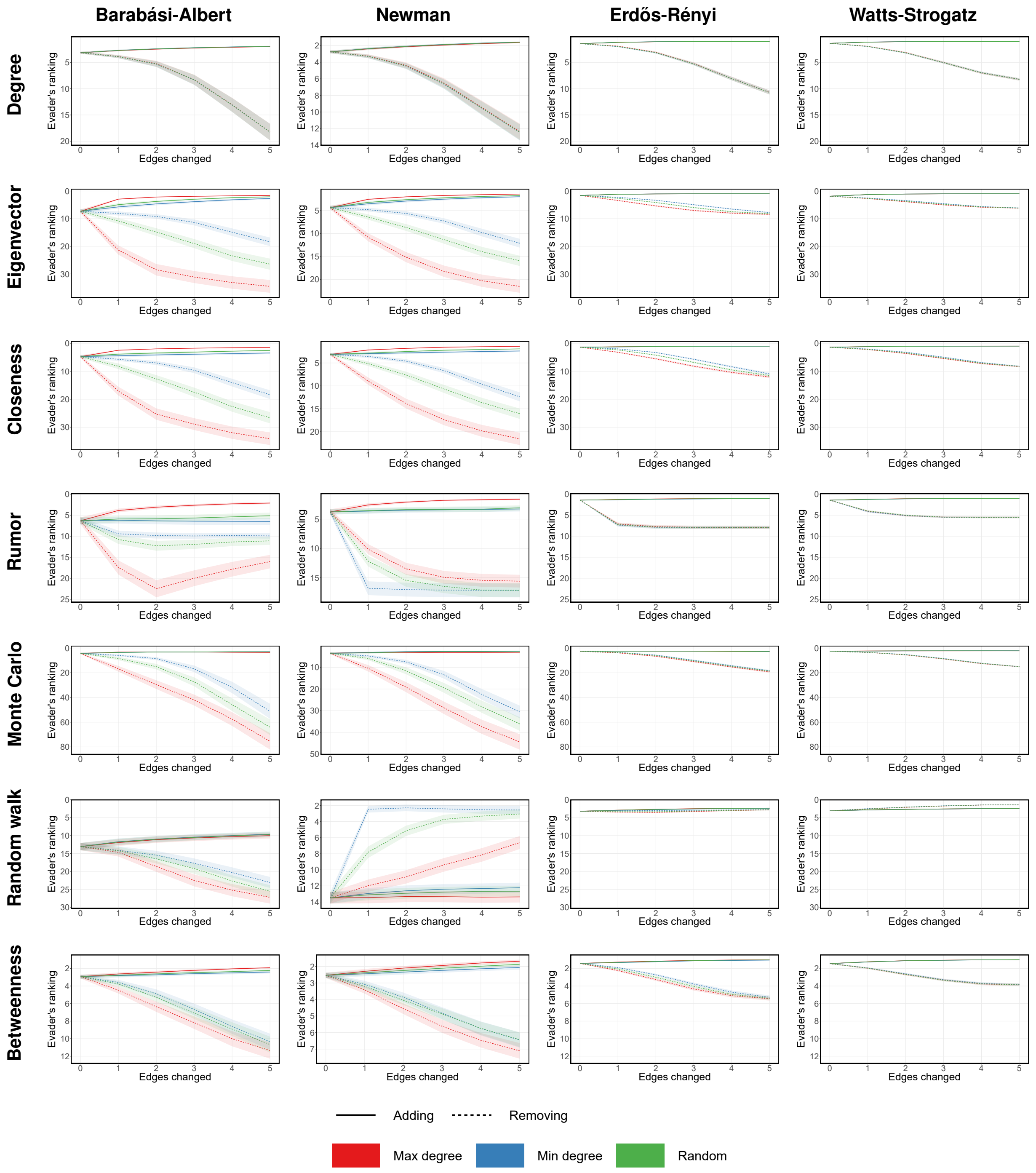

Figure S19: **Results of hiding the source of diffusion by modifying edges, related to Figure 2.** The y-axis represents the evader's ranking according to the source detection algorithm (greater value indicates more effective hiding); the x-axis corresponds to the number of edges added to, or removed from, the network. Each color corresponds to a different way of choosing edges, while each line type (dashed or solid) corresponds to either adding or removing. Results are presented for networks consisting of $1,000$ nodes with an average degree of 4. Shaded areas represent 95% confidence intervals.

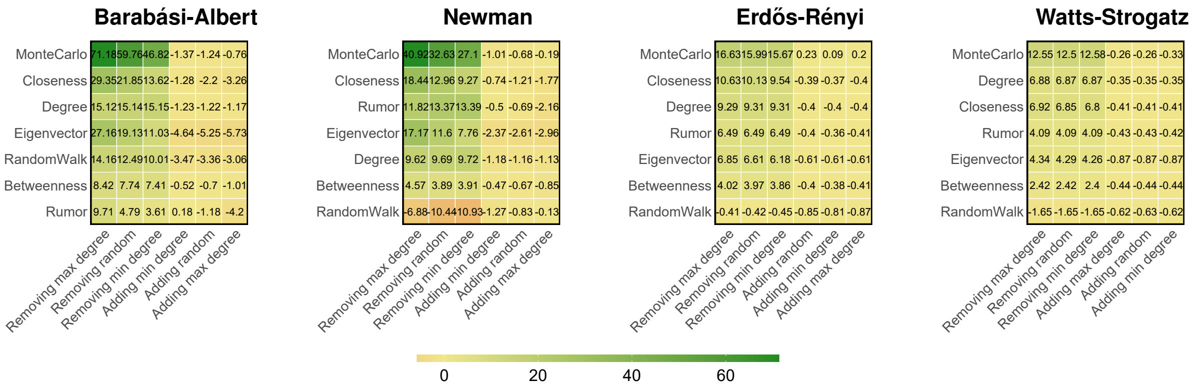

Figure S20: **Results of hiding the source of diffusion by modifying edges, related to Figure 2.** In each heatmap, rows correspond to different source detection algorithms, while columns correspond to different heuristics. The value in each cell indicates the change in the evader's ranking according to the source detection algorithm as a result of adding or removing 5 edges to the network, depending on the heuristic. Positive values indicate that the evader became more hidden, with greater values indicated a more effective disguise. In contrast, negative values indicate that the evader became less hidden. Results are presented for networks consisting of $1,000$ nodes with an average degree of 4. Rows and columns are sorted by average value.

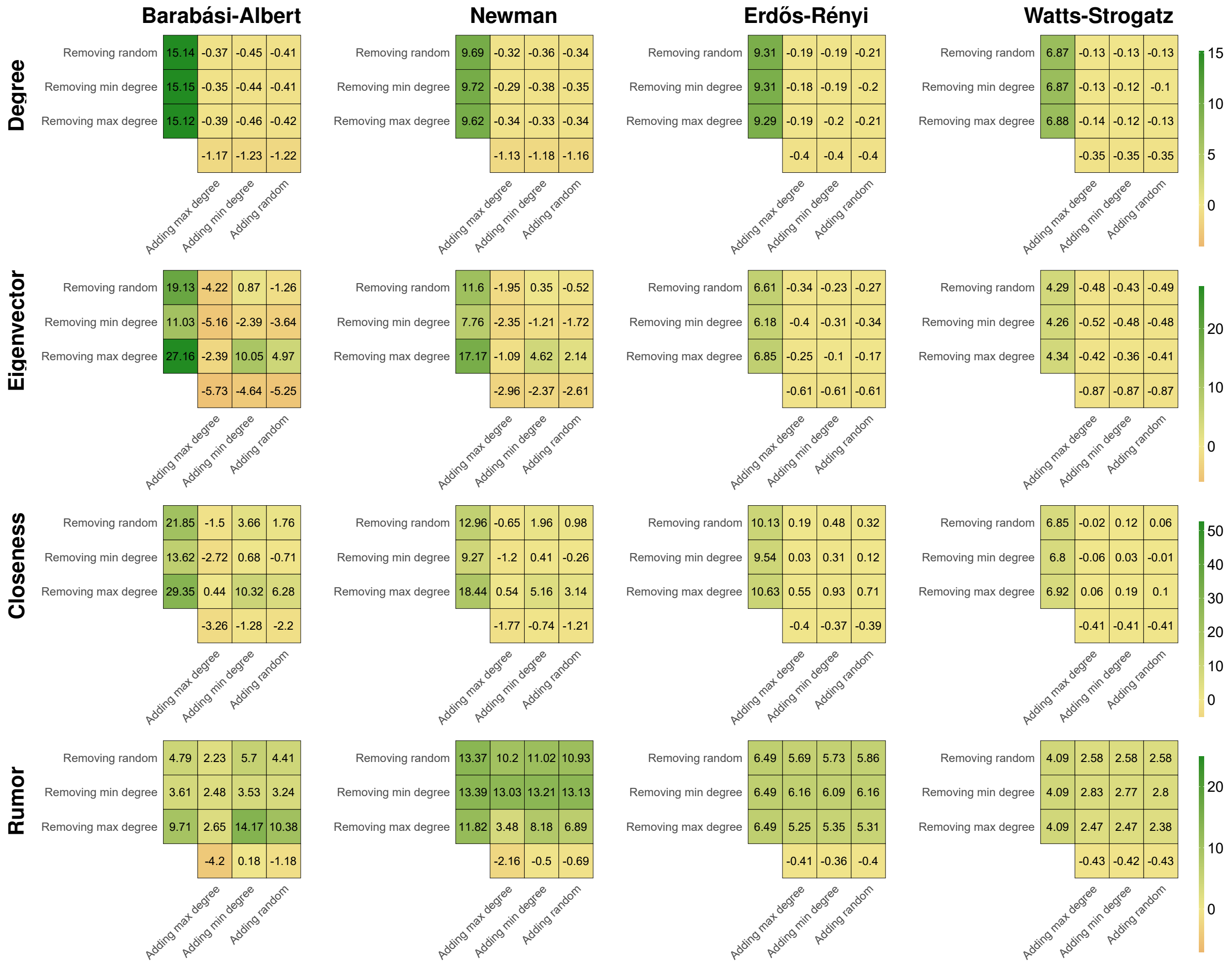

Figure S21: **Results of hiding the source of diffusion by modifying edges with mixed strategies, related to Figure 2.** In each heatmap, rows correspond to different removal heuristics, while columns correspond to different addition heuristics. The first column and the last row represent pure strategies, while the remaining cells represent mixed strategies. The value in each cell indicates the change in the evader's ranking according to the source detection algorithm after adding or removing 5 edges, depending on the heuristic. Positive values indicate that the evader became more hidden, with greater values indicated a more effective disguise. In contrast, negative values indicate that the evader became less hidden. Results are presented for networks consisting of $1,000$ nodes with an average degree of 4. Rows and columns are sorted by average value.

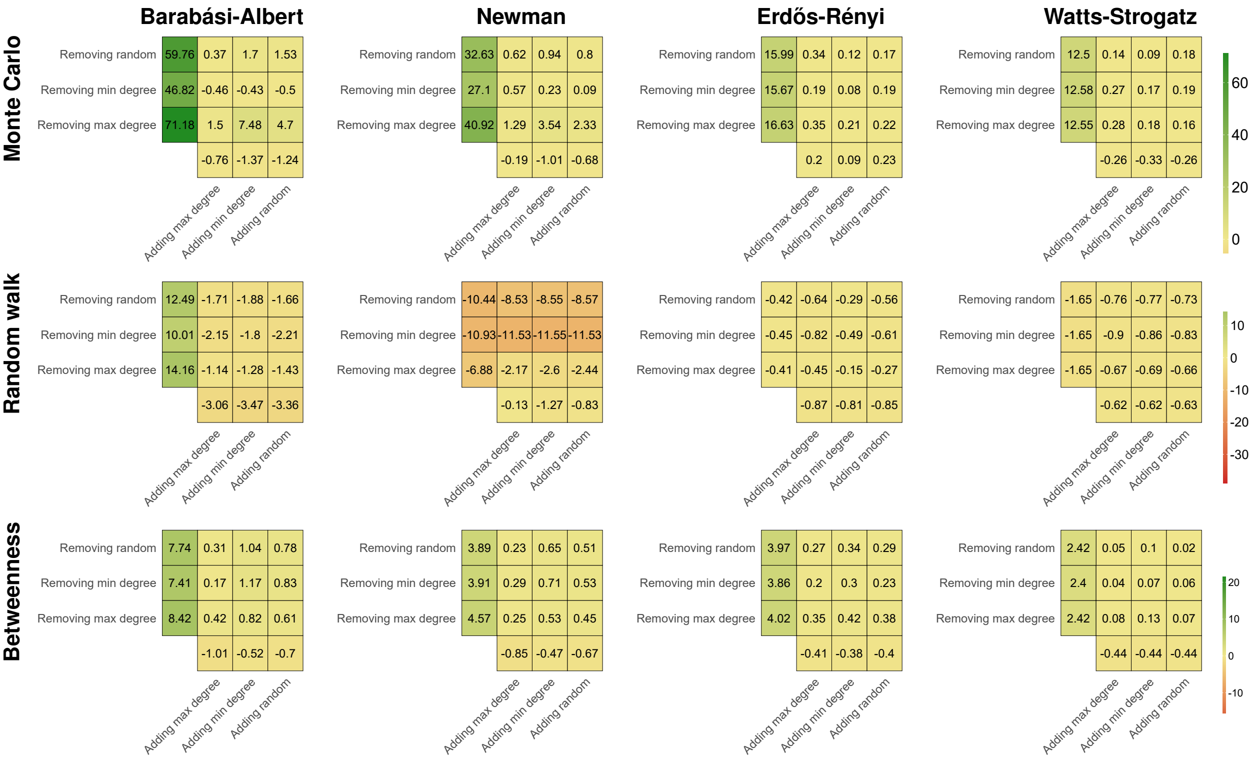

Figure S22: **Results of hiding the source of diffusion by modifying edges with mixed strategies, related to Figure 2.** In each heatmap, rows correspond to different removal heuristics, while columns correspond to different addition heuristics. The first column and the last row represent pure strategies, while the remaining cells represent mixed strategies. The value in each cell indicates the change in the evader's ranking according to the source detection algorithm after adding or removing 5 edges, depending on the heuristic. Positive values indicate that the evader became more hidden, with greater values indicated a more effective disguise. In contrast, negative values indicate that the evader became less hidden. Results are presented for networks consisting of 1,000 nodes with an average degree of 4. Rows and columns are sorted by average value.

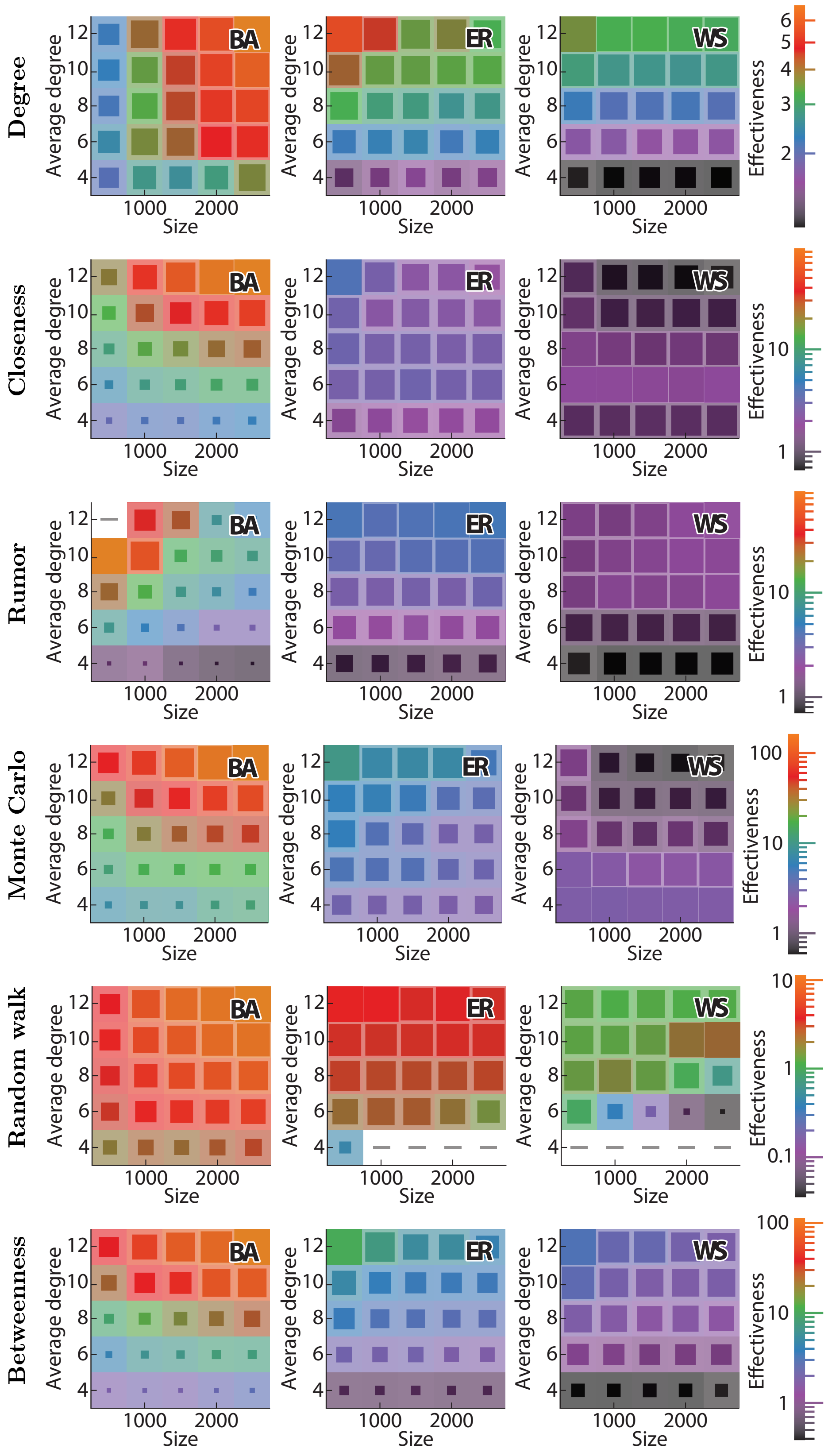

Figure S23: **Comparing the effectiveness of adding confederates vs. modifying edges, related to Figure 3.** The same as Figure 3 in the main article, except for the difference in the source detection algorithms being considered in the analysis. For cells marked with minuses, at least one type of heuristic (adding confederates or modifying edges) does not reduce the evader's ranking.

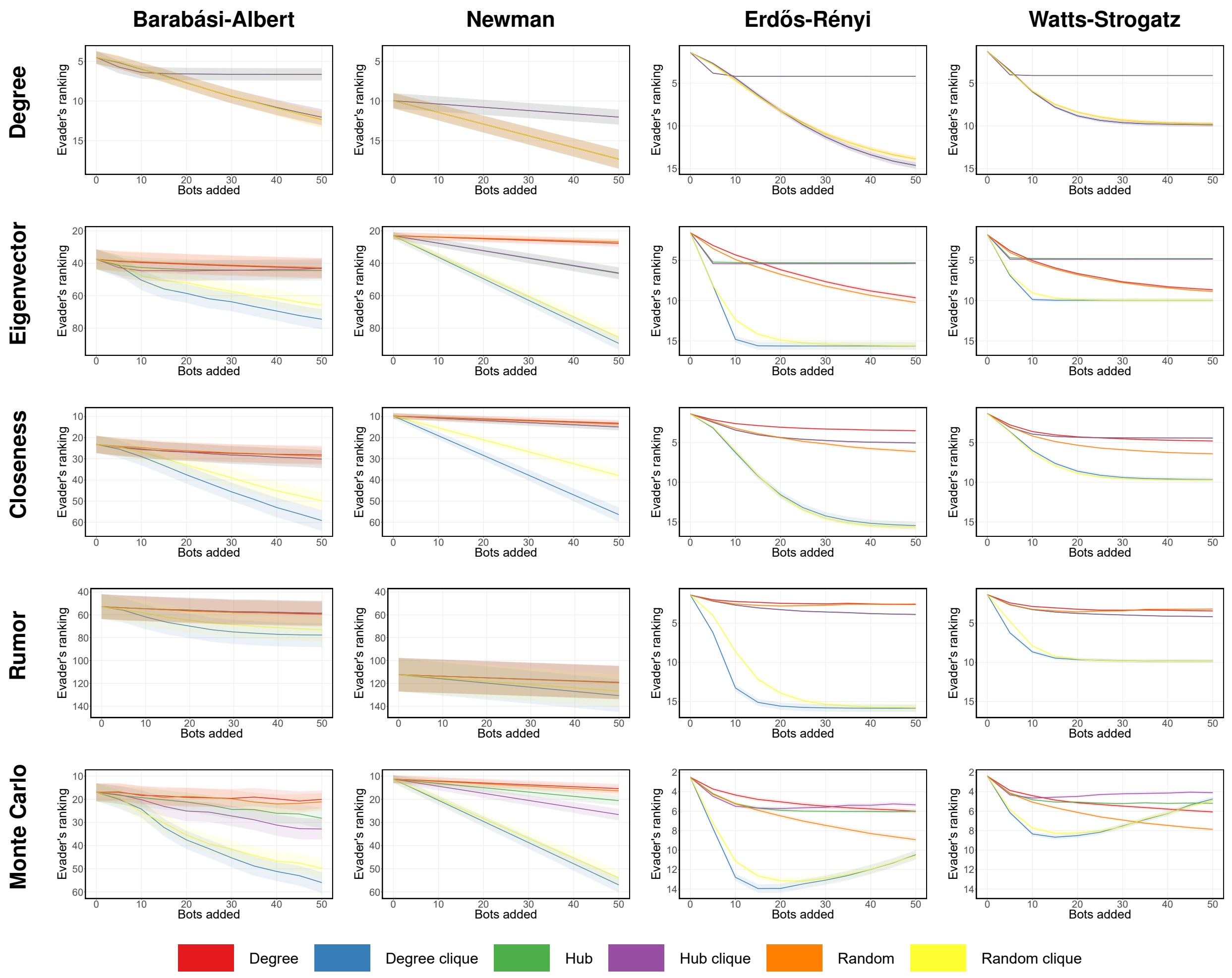

Figure S24: **The effectiveness of hiding in massive networks by adding nodes, related to Figure 5.** The y-axis corresponds to the ranking of the evader according to the source detection algorithm (greater values indicate more efficient hiding), while the x-axis corresponds to the number of nodes added to the network. Each color corresponds to a different heuristic, with each confederate being connected to three supporters. Results are presented for networks consisting of 100,000 nodes with an average degree of 4. Shaded areas represent 95% confidence intervals.

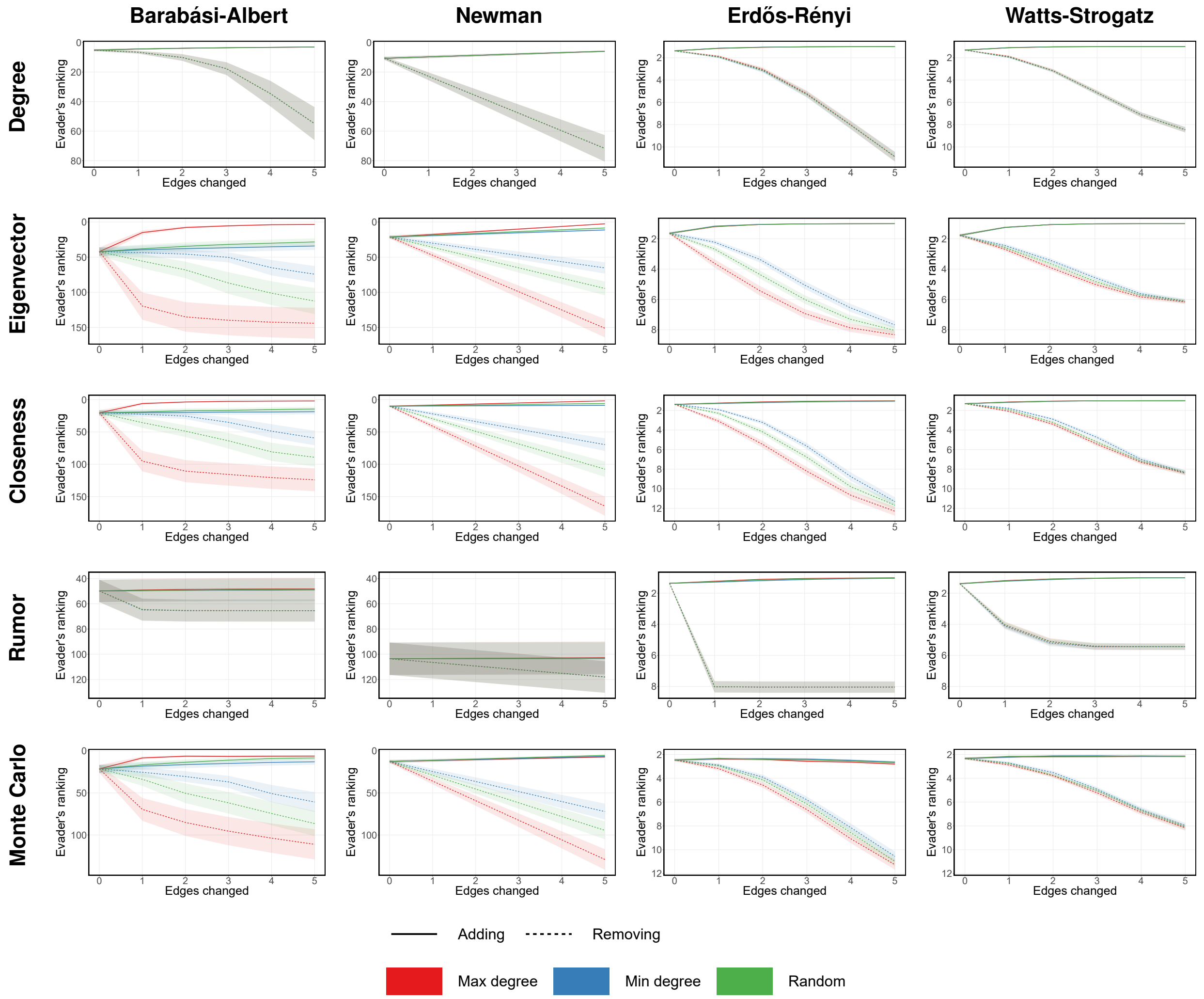

Figure S25: **The effectiveness of hiding in massive networks by modifying edges, related to Figure 5.** The y-axis represents the evader's ranking according to the source detection algorithm (greater value indicates more effective hiding); the x-axis corresponds to the number of edges added to, or removed from, the network. Each color corresponds to a different way of choosing edges, while each line type (dashed or solid) corresponds to either adding or removing. Results are presented for networks consisting of 100,000 nodes with an average degree of 4. Shaded areas represent 95% confidence intervals.

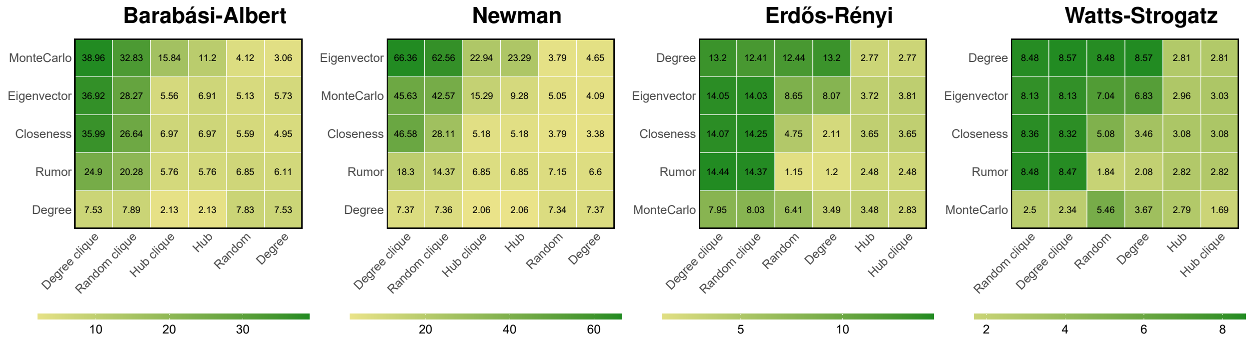

Figure S26: **Results of hiding in massive networks by adding nodes, related to Figure 5.** The y-axis of each heatmap corresponds to different source detection algorithms, whereas the x-axis corresponds to different heuristics. The value in each cell indicates the change in the evader's ranking according to the source detection algorithm after adding 50 confederates to the network and connecting each to 3 supporters using the heuristic. Results are presented for networks consisting of 100, 000 nodes with an average degree of 4. Rows and columns are sorted by average value.

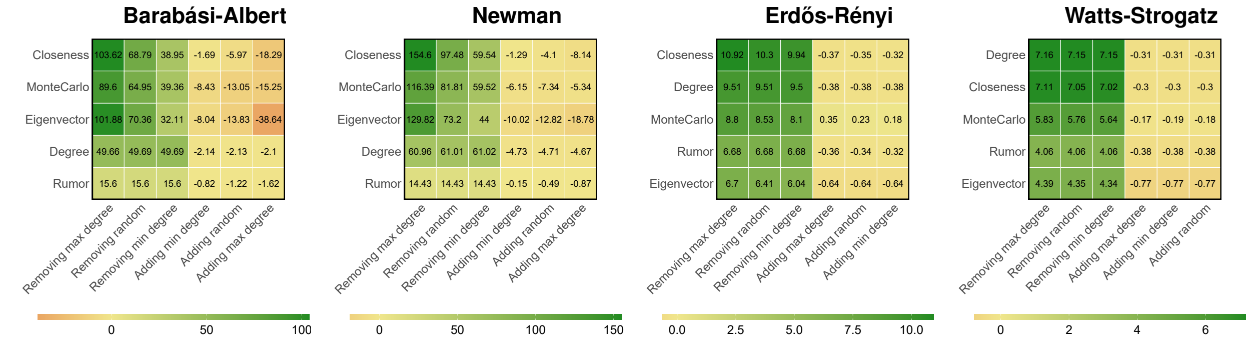

Figure S27: **Results of hiding in massive networks by modifying edges, related to Figure 5.** In each heatmap, rows correspond to different source detection algorithms, while columns correspond to different heuristics. The value in each cell indicates the change in the evader's ranking according to the source detection algorithm as a result of adding or removing 5 edges to the network, depending on the heuristic. Positive values indicate that the evader became more hidden, with greater values indicated a more effective disguise. In contrast, negative values indicate that the evader became less hidden. Results are presented for networks consisting of 100, 000 nodes with an average degree of 4. Rows and columns are sorted by average value.

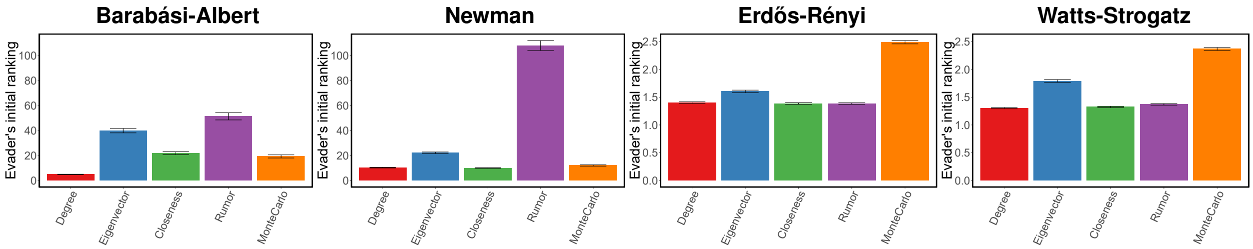

Figure S28: **Comparison of the effectiveness of different source detection algorithms before the hiding process in random networks consisting of 100,000 nodes, related to Figure 5.** The x-axis corresponds to different source detection algorithms, while the y-axis corresponds to the initial ranking of the evader. The error bars represent 95% confidence intervals.

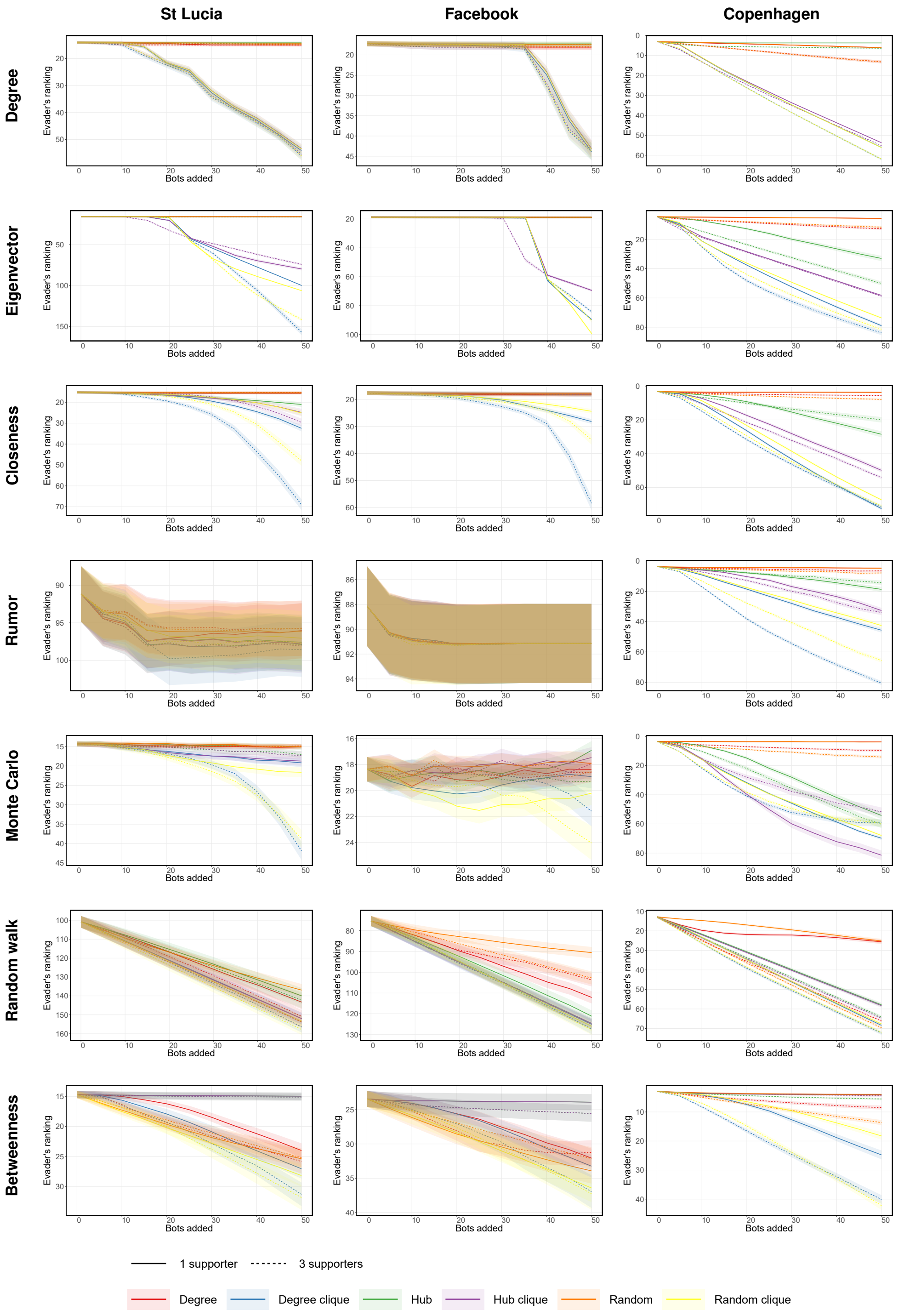

Figure S29: **Results hiding the source of diffusion by adding nodes in small real-life networks, related to Figure 6.** The y-axis corresponds to the ranking of the evader according to the source detection algorithm (greater values indicate more efficient hiding), while the x-axis corresponds to the number of nodes added to the network. Each color corresponds to a different heuristic. Solid lines correspond to cases where each confederate is connected to a single supporter, while dashed lines correspond to cases where each confederate is connected to three supporters. Shaded areas represent 95% confidence intervals.

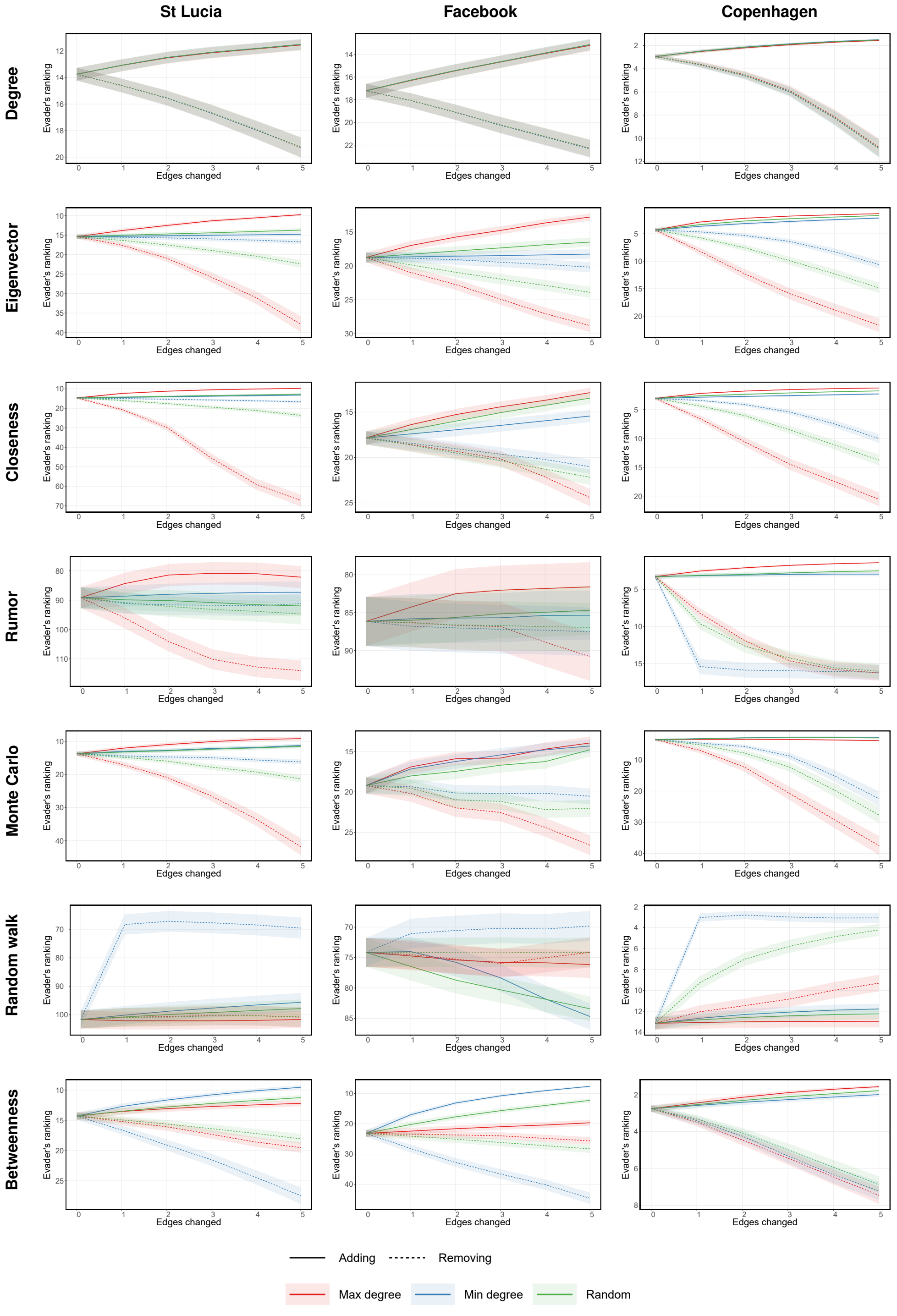

Figure S30: **Results of hiding the source of diffusion by modifying edges in small real-life networks, related to Figure 6.** The y-axis represents the evader's ranking according to the source detection algorithm (greater value indicates more effective hiding); the x-axis corresponds to the number of edges added to, or removed from, the network. Each color corresponds to a different way of choosing edges, while each line type (dashed or solid) corresponds to either adding or removing. Shaded areas represent 95% confidence intervals.

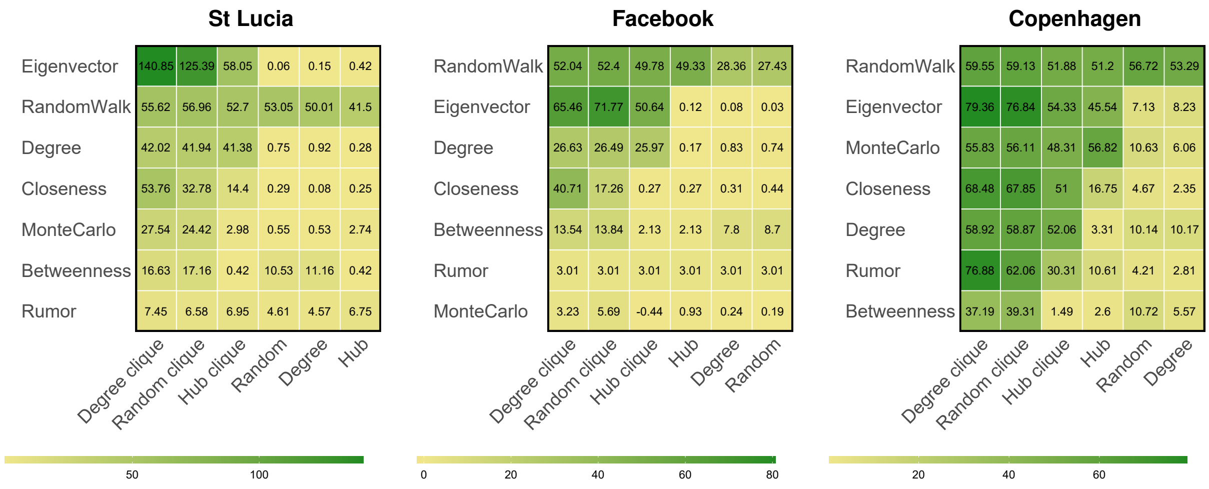

Figure S31: **Results of hiding the source of diffusion by adding nodes in small real-life networks, related to Figure 6.** The y-axis of each heatmap corresponds to different source detection algorithms, whereas the x-axis corresponds to different heuristics. The value in each cell indicates the change in the evader's ranking according to the source detection algorithm after adding 50 confederates to the network using the heuristic. Rows and columns are sorted by average value.

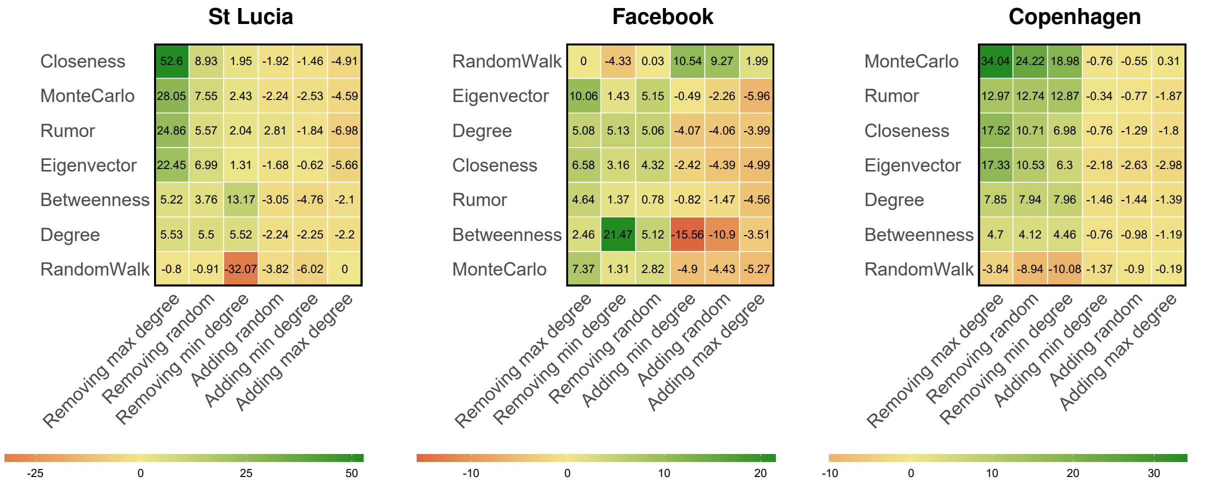

Figure S32: **Results of hiding the source of diffusion by modifying edges in small real-life networks, related to Figure 6.** In each heatmap, rows correspond to different source detection algorithms, while columns correspond to different heuristics. The value in each cell indicates the change in the evader's ranking according to the source detection algorithm as a result of adding or removing 5 edges to the network, depending on the heuristic. Positive values indicate that the evader became more hidden, with greater values indicated a more effective disguise. In contrast, negative values indicate that the evader became less hidden. Rows and columns are sorted by average value.

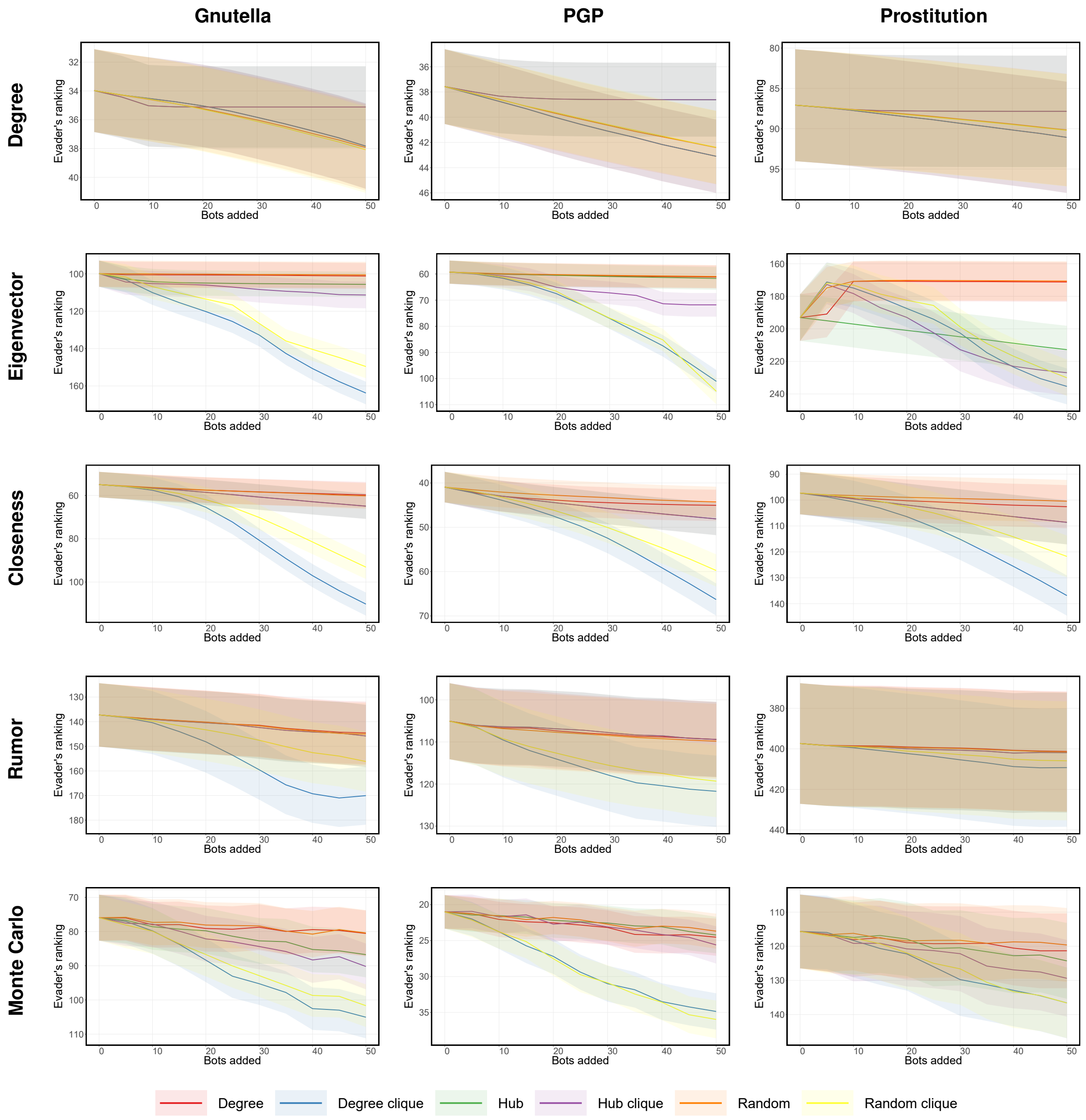

Figure S33: **The effectiveness of hiding by adding nodes in large real-life networks, related to Figure 6.** The y-axis corresponds to the ranking of the evader according to the source detection algorithm (greater values indicate more efficient hiding), while the x-axis corresponds to the number of nodes added to the network. Each color corresponds to a different heuristic, with each confederate being connected to three supporters. Shaded areas represent 95% confidence intervals.

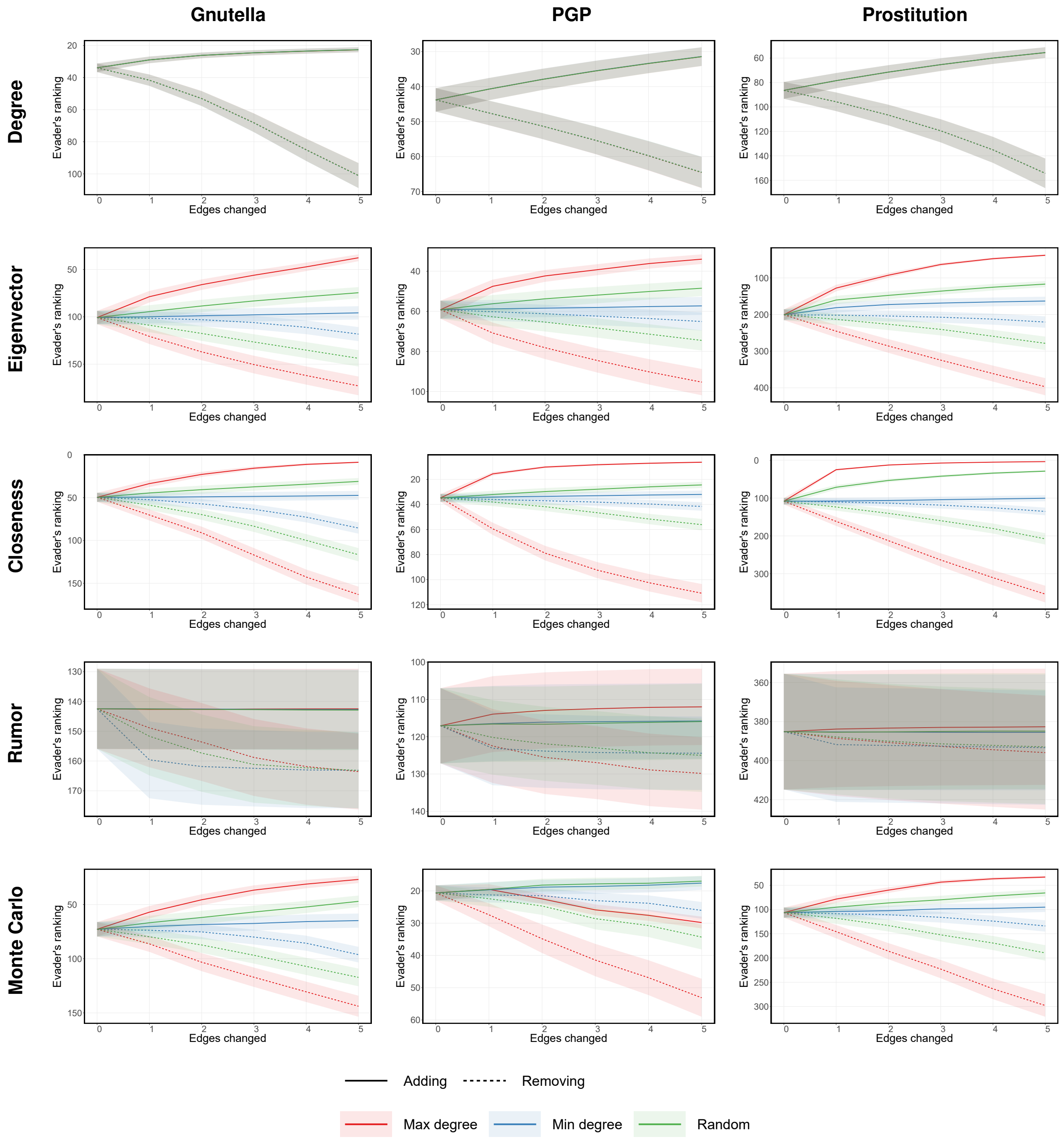

Figure S34: **The effectiveness of hiding by modifying edges in large real-life networks, related to Figure 6.** The y-axis represents the evader's ranking according to the source detection algorithm (greater value indicates more effective hiding); the x-axis corresponds to the number of edges added to, or removed from, the network. Each color corresponds to a different way of choosing edges, while each line type (dashed or solid) corresponds to either adding or removing. Shaded areas represent 95% confidence intervals.

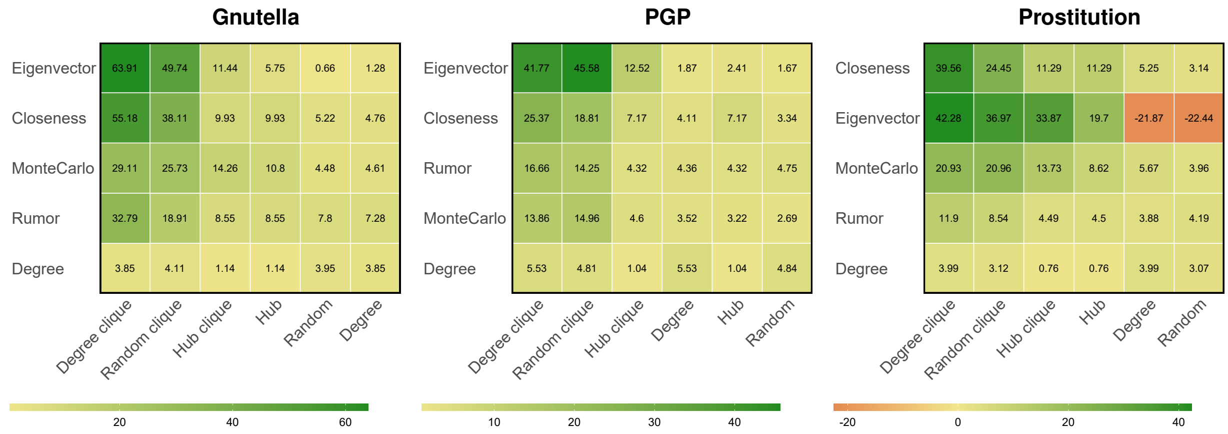

Figure S35: **Results of hiding by adding nodes in large real-life networks, related to Figure 6.** The y-axis of each heatmap corresponds to different source detection algorithms, whereas the x-axis corresponds to different heuristics. The value in each cell indicates the change in the evader's ranking according to the source detection algorithm after adding 50 confederates to the network and connecting each to 3 supporters using the heuristic. Rows and columns are sorted by average value.

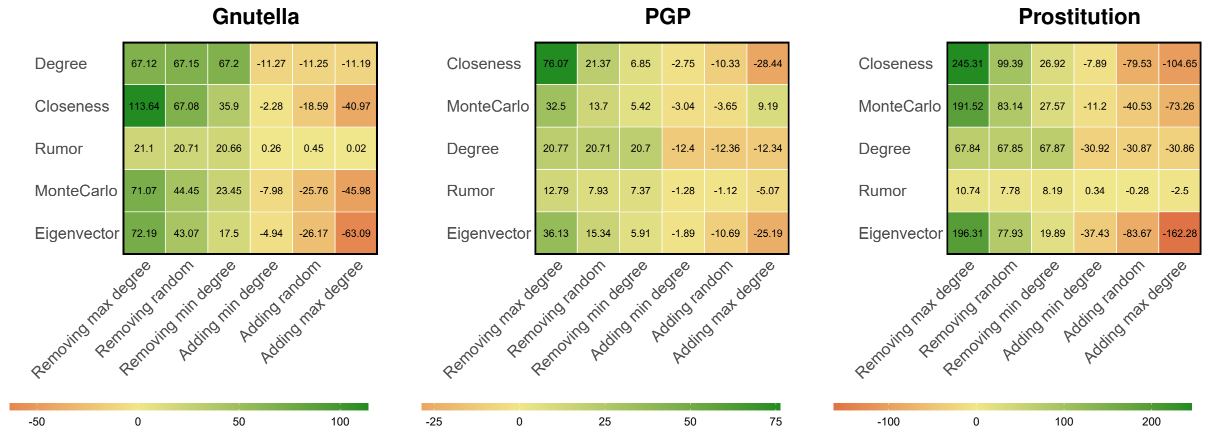

Figure S36: **Results of hiding by modifying edges in large real-life networks, related to Figure 6.** In each heatmap, rows correspond to different source detection algorithms, while columns correspond to different heuristics. The value in each cell indicates the change in the evader's ranking according to the source detection algorithm as a result of adding or removing 5 edges to the network, depending on the heuristic. Positive values indicate that the evader became more hidden, with greater values indicated a more effective disguise. In contrast, negative values indicate that the evader became less hidden. Rows and columns are sorted by average value.

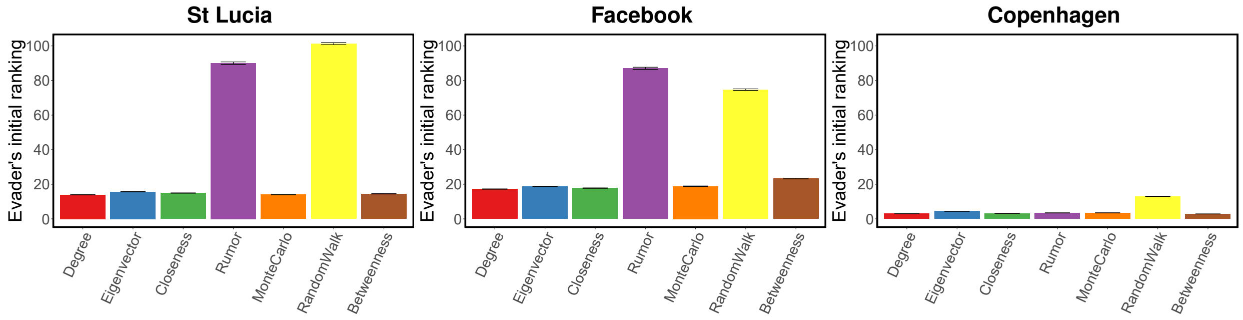

Figure S37: **Comparison of the effectiveness of different source detection algorithms before the hiding process in small real-life networks, related to Figure 6.** The x-axis corresponds to different source detection algorithms, while the y-axis corresponds to the initial ranking of the evader. The error bars represent 95% confidence intervals.

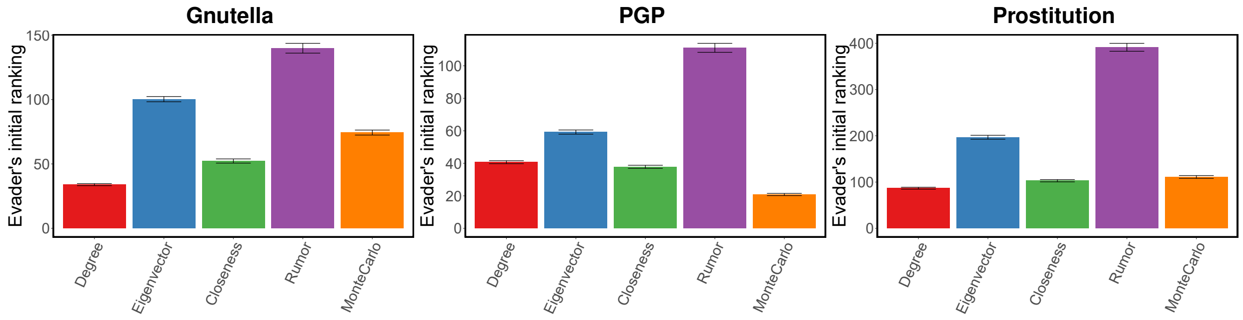

Figure S38: **Comparison of the effectiveness of different source detection algorithms before the hiding process in large real-life networks, related to Figure 6.** The x-axis corresponds to different source detection algorithms, while the y-axis corresponds to the initial ranking of the evader. The error bars represent 95% confidence intervals.

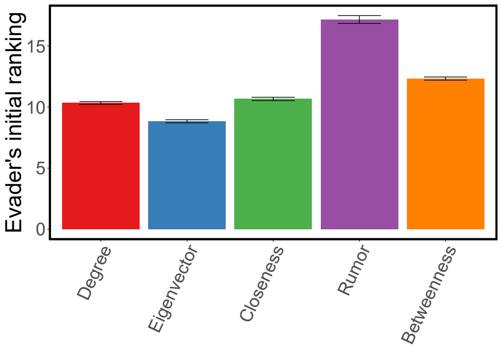

Figure S39: **Comparing the effectiveness of source detection algorithms for real-life cascades before the hiding process is initiated, related to Figure 6.** The x-axis corresponds to different source detection algorithms, while the y-axis corresponds to the initial ranking of the evader (i.e., their ranking before attempting to hide). Results are averaged over the eight cascades in the real-life dataset, with error bars representing 95% confidence intervals.

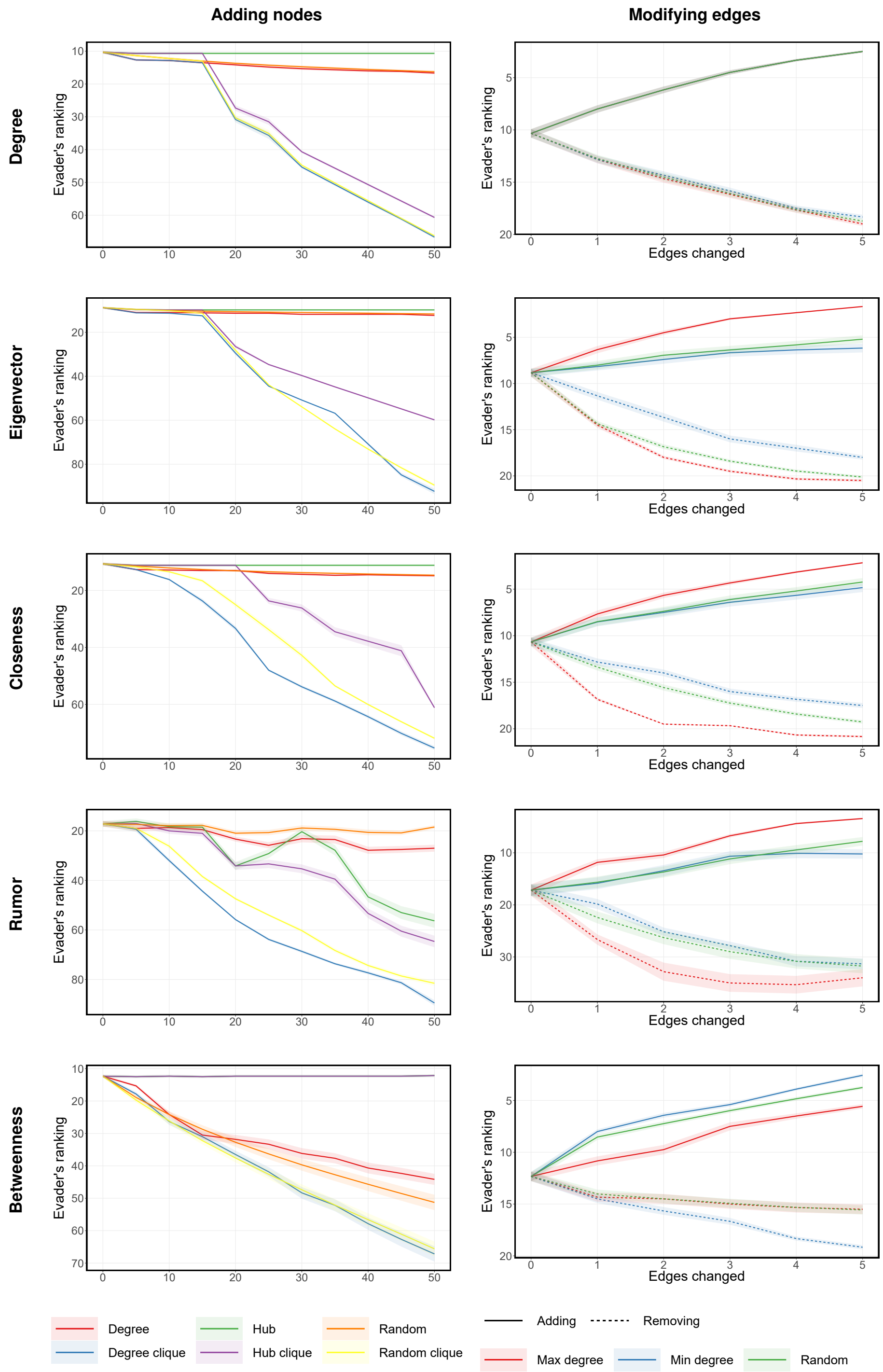

Figure S40: **Results of hiding the source of real-life cascades, related to Figure 6.** The y-axis corresponds to the ranking of the evader according to the source detection algorithm (greater values indicate more efficient hiding), while the x-axis corresponds to the number of nodes added to the network (left column) or the number of edges added to, or removed from, the network (right column). Each line correspond to different heuristics. Results are averaged over the eight cascades in the real-life dataset, with shaded areas representing 95% confidence intervals.

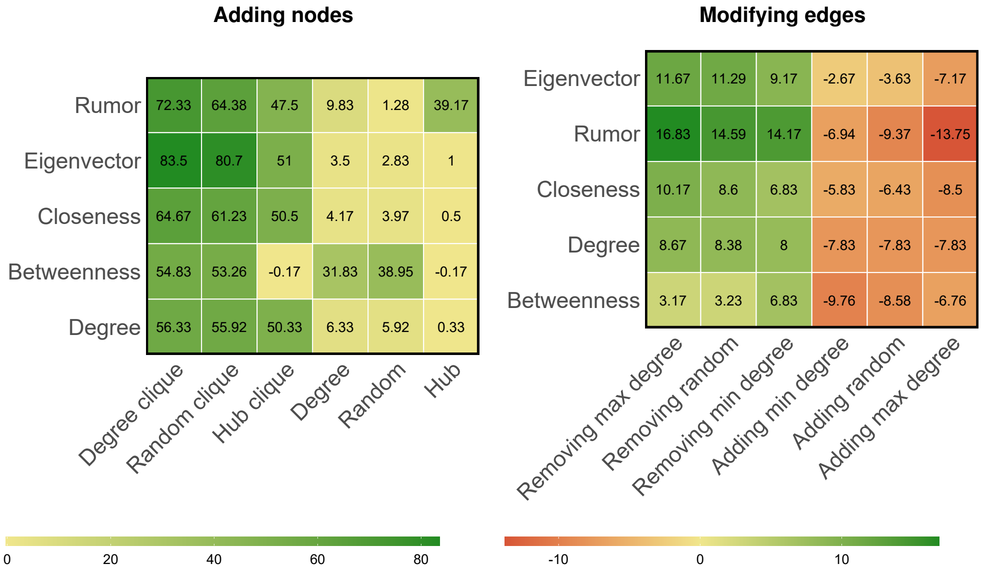

Figure S41: **Results of hiding the source of real-life cascades, related to Figure 6.** The y-axis of each heatmap corresponds to different source detection algorithms, whereas the x-axis corresponds to different heuristics. The value in each cell indicates the change in the evader's ranking according to the source detection algorithm after adding 50 confederates to the network (left plot) or after adding or removing 5 edges to the network (right plot). Rows and columns are sorted by average value, while the value in each cell is averaged over the eight cascades in the real-life dataset.

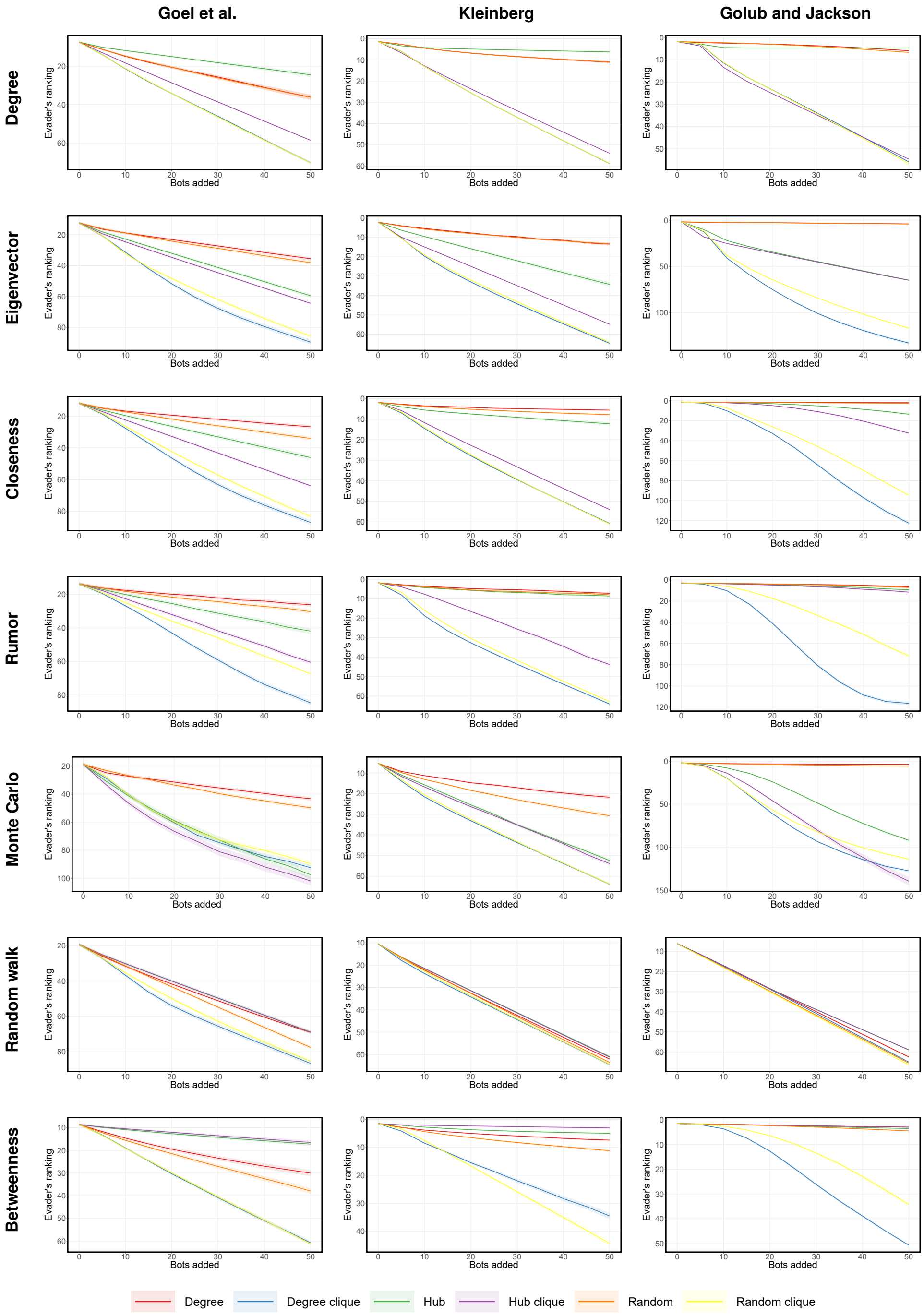

Figure S42: **Results of hiding the source of diffusion by adding nodes for alternative models of diffusion and network generation, related to STAR Methods.** The y-axis corresponds to the ranking of the evader according to the source detection algorithm (greater values indicate more efficient hiding), while the x-axis corresponds to the number of nodes added to the network. Each color corresponds to a different heuristic, with each confederate being connected to three supporters. Shaded areas represent 95% confidence intervals.

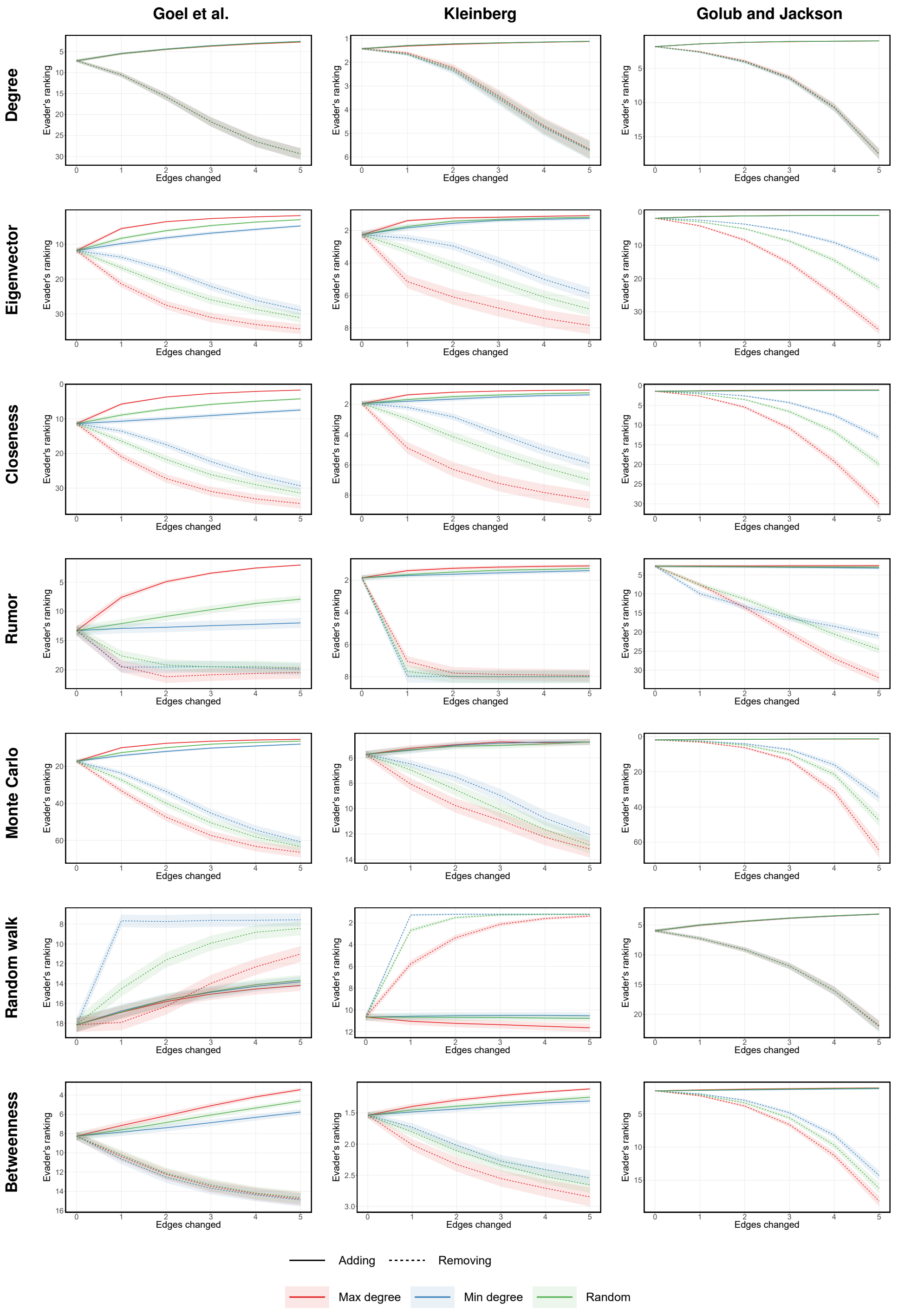

Figure S43: **Results of hiding the source of diffusion by modifying edges for alternative models of diffusion and network generation, related to STAR Methods.** The y-axis represents the evader's ranking according to the source detection algorithm (greater value indicates more effective hiding); the x-axis corresponds to the number of edges added to, or removed from, the network. Each color corresponds to a different way of choosing edges, while each line type corresponds to either adding or removing. Shaded areas represent 95% confidence intervals.

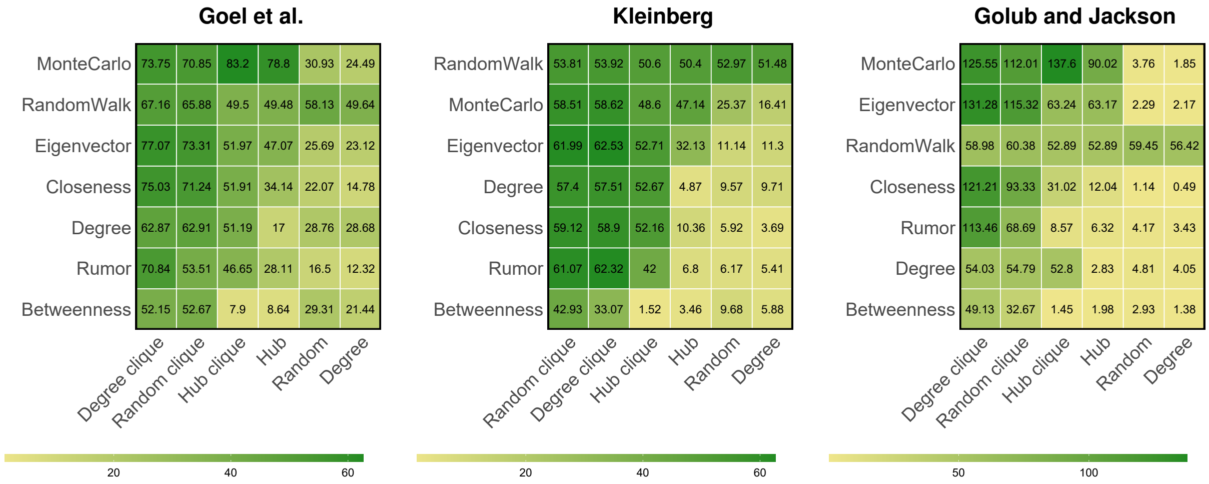

Figure S44: **Results of hiding the source of diffusion by adding nodes for alternative models of diffusion and network generation, related to STAR Methods.** The y-axis of each heatmap corresponds to different source detection algorithms, whereas the x-axis corresponds to different heuristics. The value in each cell indicates the change in the evader's ranking according to the source detection algorithm after adding 50 confederates to the network using the heuristic. Rows and columns are sorted by average value.

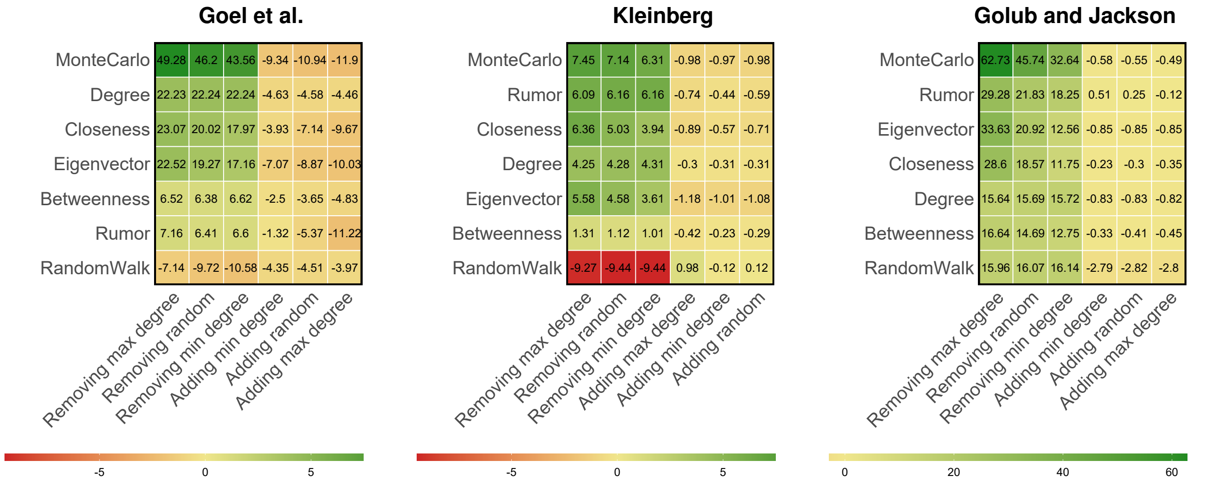

Figure S45: **Results of hiding the source of diffusion by modifying edges for alternative models of diffusion and network generation, related to STAR Methods.** In each heatmap, rows correspond to different source detection algorithms, while columns correspond to different heuristics. The value in each cell indicates the change in the evader's ranking according to the source detection algorithm as a result of adding or removing 5 edges to the network, depending on the heuristic. Positive values indicate that the evader became more hidden, with greater values indicated a more effective disguise. In contrast, negative values indicate that the evader became less hidden. Rows and columns are sorted by average value.

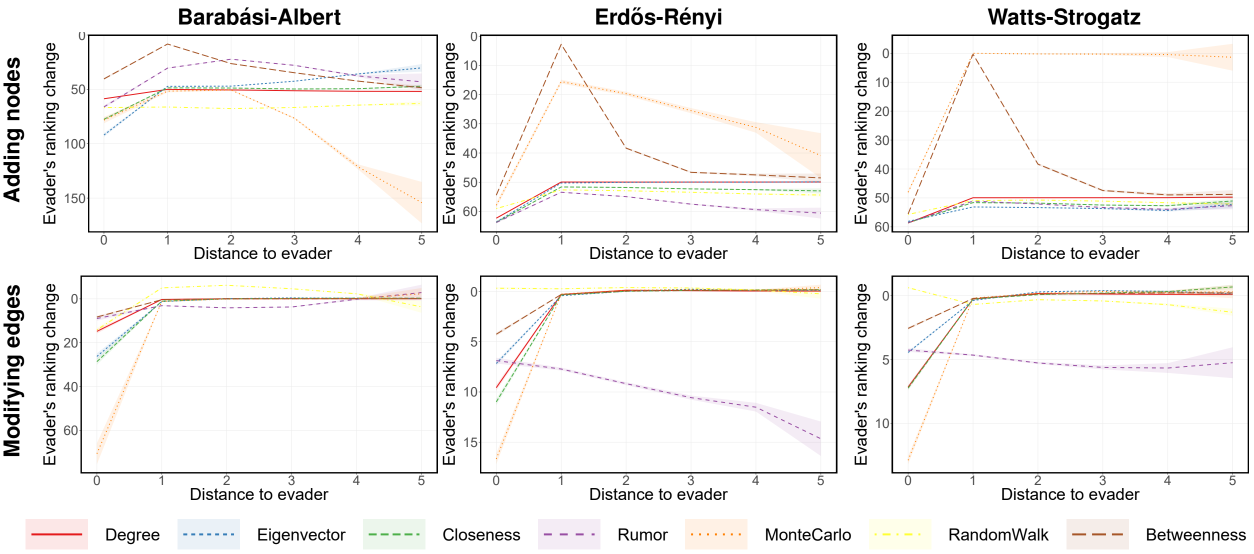

Figure S46: **Evaluating how the evader's ranking is affected when nearby nodes attempt to hide, related to STAR Methods.** The x-axis corresponds to the distance between the node running the heuristic and the evader in an unmodified network, i.e., before hiding (notice that distance 0 indicates that the evader is the one running the heuristic). The y-axis represents the change in the evader's ranking according to the source detection algorithm (greater value indicates more effective hiding). Results are presented for networks consisting of $1,000$ nodes with an average degree of 4. Shaded areas represent 95% confidence intervals.

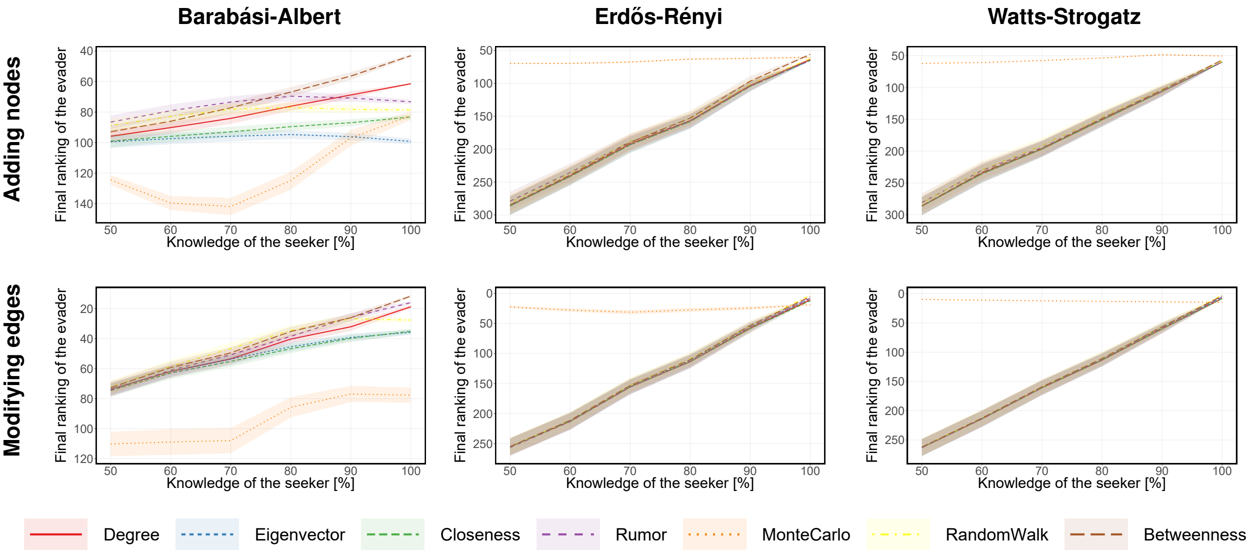

Figure S47: **Effects of imperfect knowledge of the seeker, related to STAR Methods.** The x-axis corresponds to the percentage of the network's edges that are visible to the seeker. The y-axis represents the final ranking the evader according to different source detection algorithms after the hiding process (greater value indicates more effective hiding). Results are presented for networks consisting of 1,000 nodes with an average degree of 4. Shaded areas represent 95% confidence intervals.

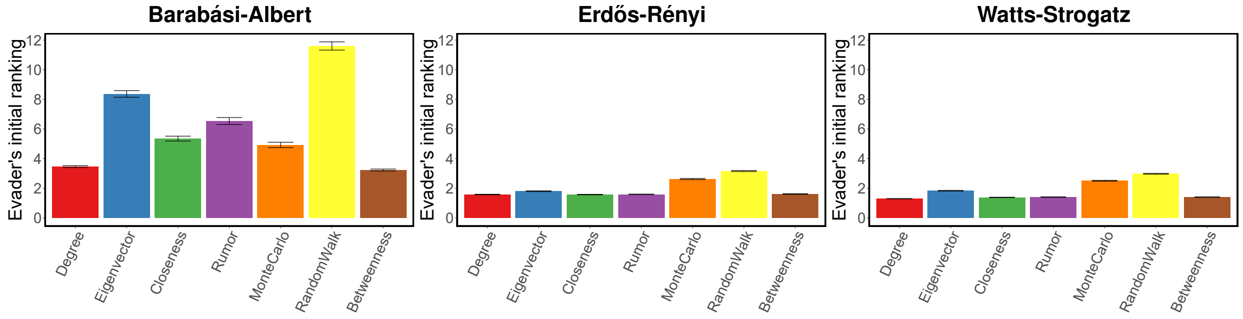

Figure S48: **Comparison of the effectiveness of different source detection algorithms before the hiding process when the evader is selected uniformly at random, related to STAR Methods.** The x-axis corresponds to different source detection algorithms, while the y-axis corresponds to the evader's ranking according to the different algorithms. Results are presented for networks consisting of 1,000 nodes with an average degree of 4. The error bars represent 95% confidence intervals.

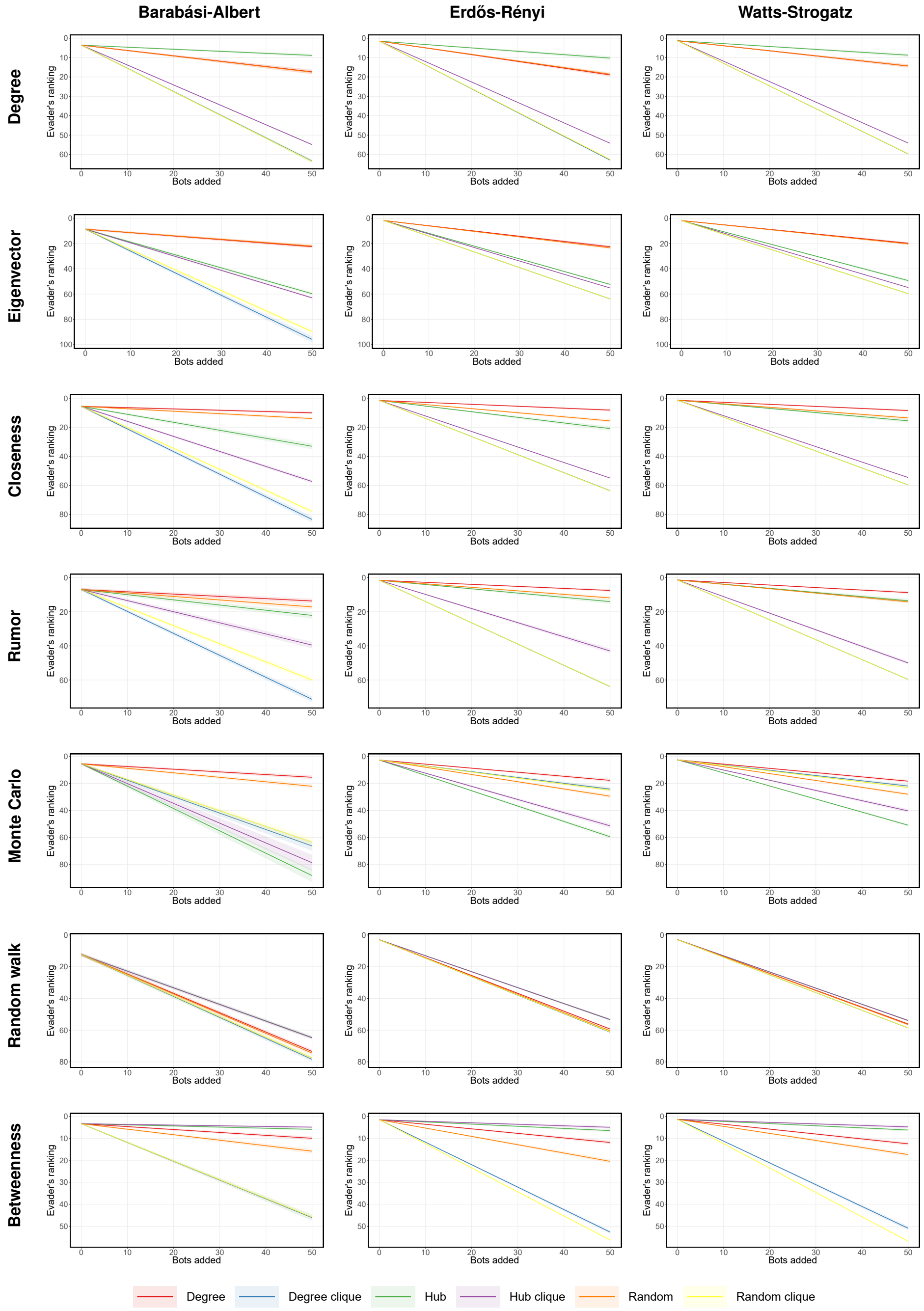

Figure S49: **Results of hiding the source of diffusion by adding nodes when the evader is selected uniformly at random, related to STAR Methods.** The y-axis corresponds to the ranking of the evader according to the source detection algorithm (greater values indicate more efficient hiding), while the x-axis corresponds to the number of nodes added to the network. Each color corresponds to a different heuristic, with each confederate being connected to three supporters. Results are presented for networks consisting of $1,000$ nodes with an average degree of 4. Shaded areas represent 95% confidence intervals.

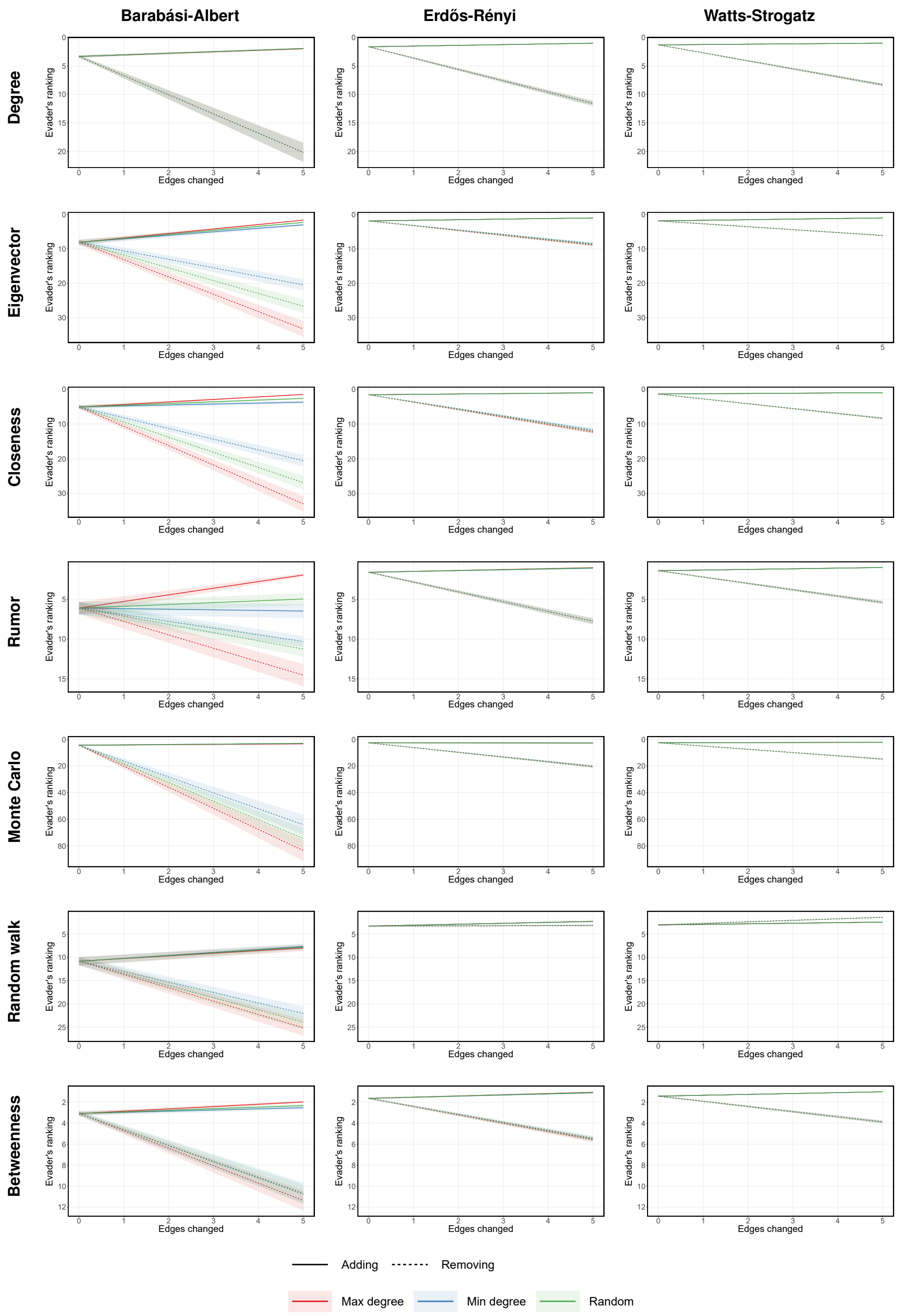

Figure S50: **Results of hiding the source of diffusion by modifying edges when the evader is selected uniformly at random, related to STAR Methods.** The y-axis represents the evader's ranking according to the source detection algorithm (greater value indicates more effective hiding); the x-axis corresponds to the number of edges added to, or removed from, the network. Each color corresponds to a different way of choosing edges, while each line type corresponds to either adding or removing edges. Results are presented for networks consisting of 1,000 nodes with an average degree of 4. Shaded areas represent 95% confidence intervals.

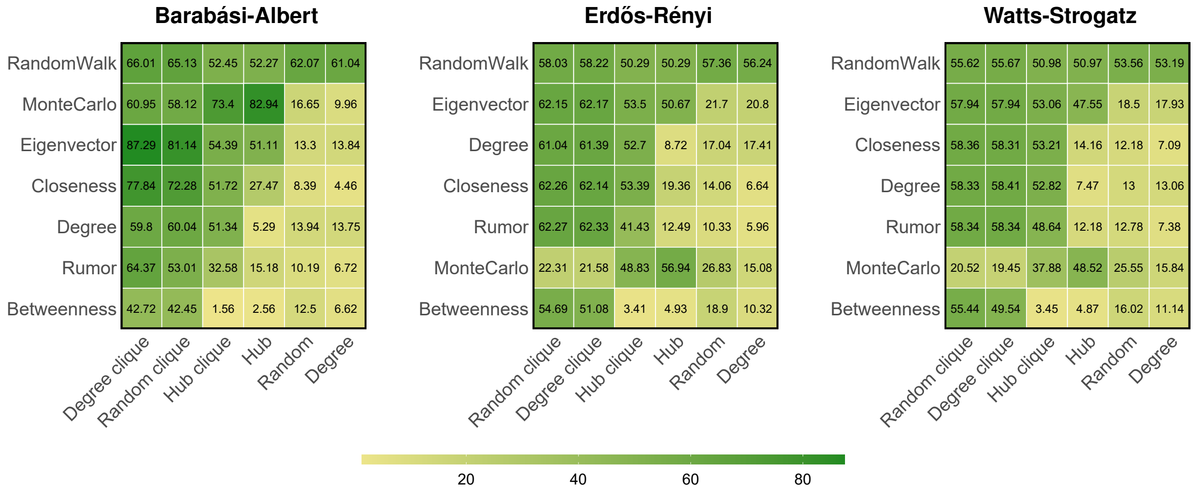

Figure S51: **Results of hiding the source of diffusion by adding nodes when the evader is selected uniformly at random, related to STAR Methods.** The y-axis of each heatmap corresponds to different source detection algorithms, whereas the x-axis corresponds to different heuristics. The value in each cell indicates the change in the evader's ranking according to the source detection algorithm after adding 50 confederates to the network using the heuristic. Results are presented for networks consisting of 1,000 nodes with an average degree of 4. Rows and columns are sorted by average value.

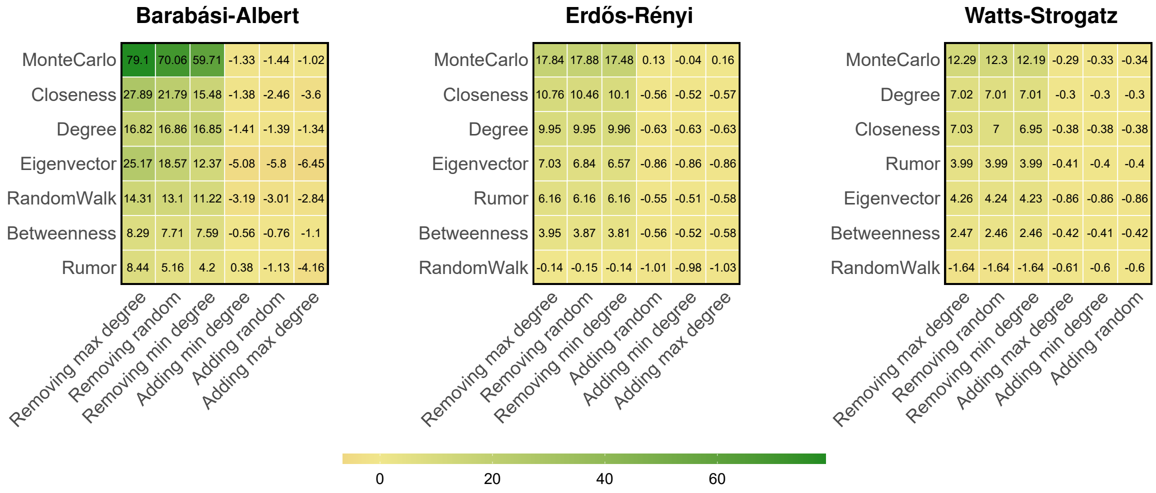

Figure S52: **Results of hiding the source of diffusion by modifying edges for alternative models of diffusion and network generation, related to STAR Methods.** In each heatmap, rows correspond to different source detection algorithms, while columns correspond to different heuristics. The value in each cell indicates the change in the evader's ranking according to the source detection algorithm as a result of adding or removing 5 edges to the network, depending on the heuristic. Positive values indicate that the evader became more hidden, with greater values indicated a more effective disguise. In contrast, negative values indicate that the evader became less hidden. Results are presented for networks consisting of 1,000 nodes with an average degree of 4. Rows and columns are sorted by average value.

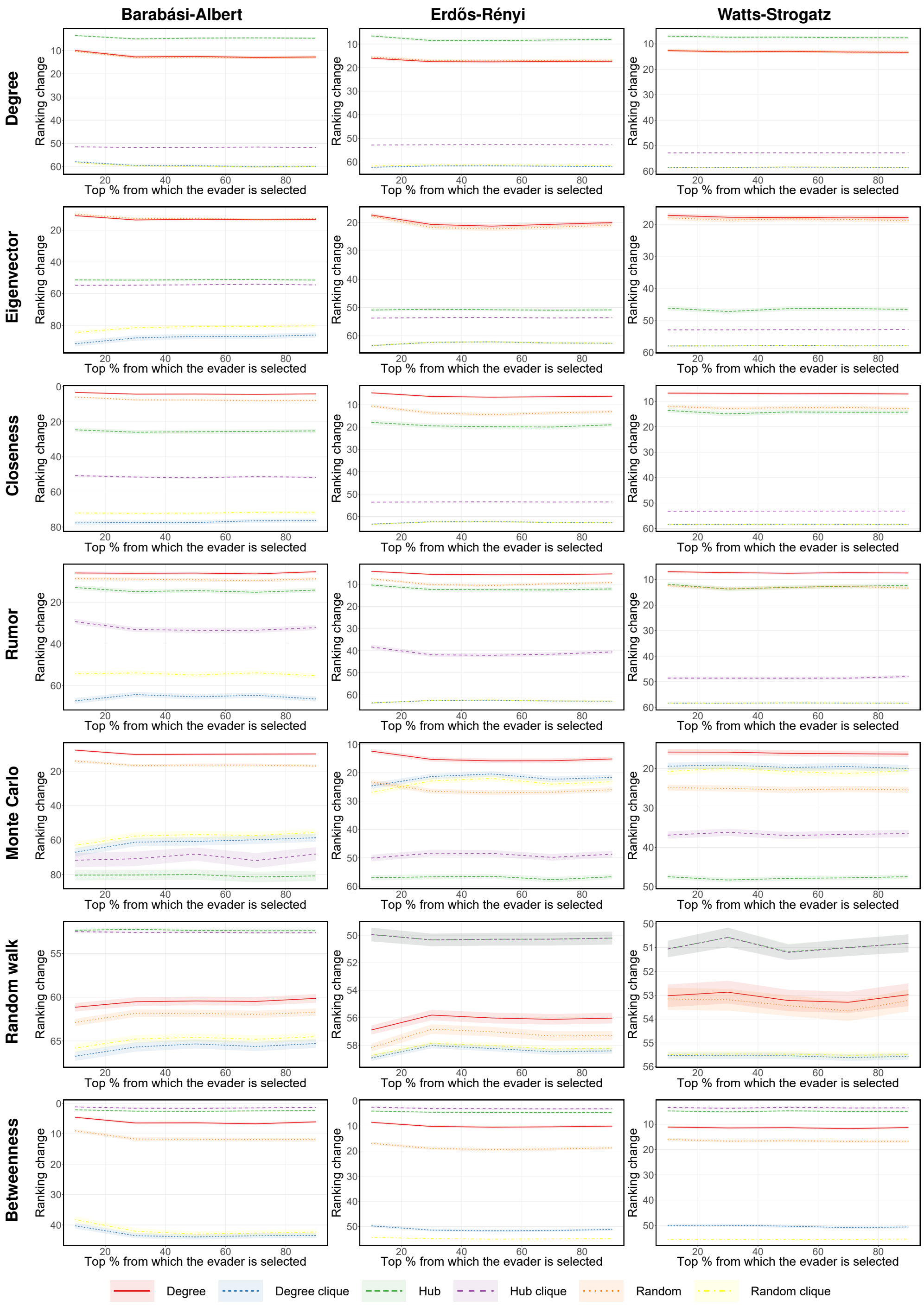

Figure S53: **Impact of the way of selecting the evader on the effectiveness of the evader's hiding for heuristics that add nodes, related to STAR Methods.** The x-axis corresponds to the percentage of top-ranked nodes (according to degree) from which the evader is randomly selected (e.g., $x = 30\%$ corresponds to the case when the evader is selected randomly from the 30% with the highest degrees). The y-axis represents the change in the evader's ranking after the hiding process (greater value indicates more effective hiding). Shaded areas represent 95% confidence intervals.

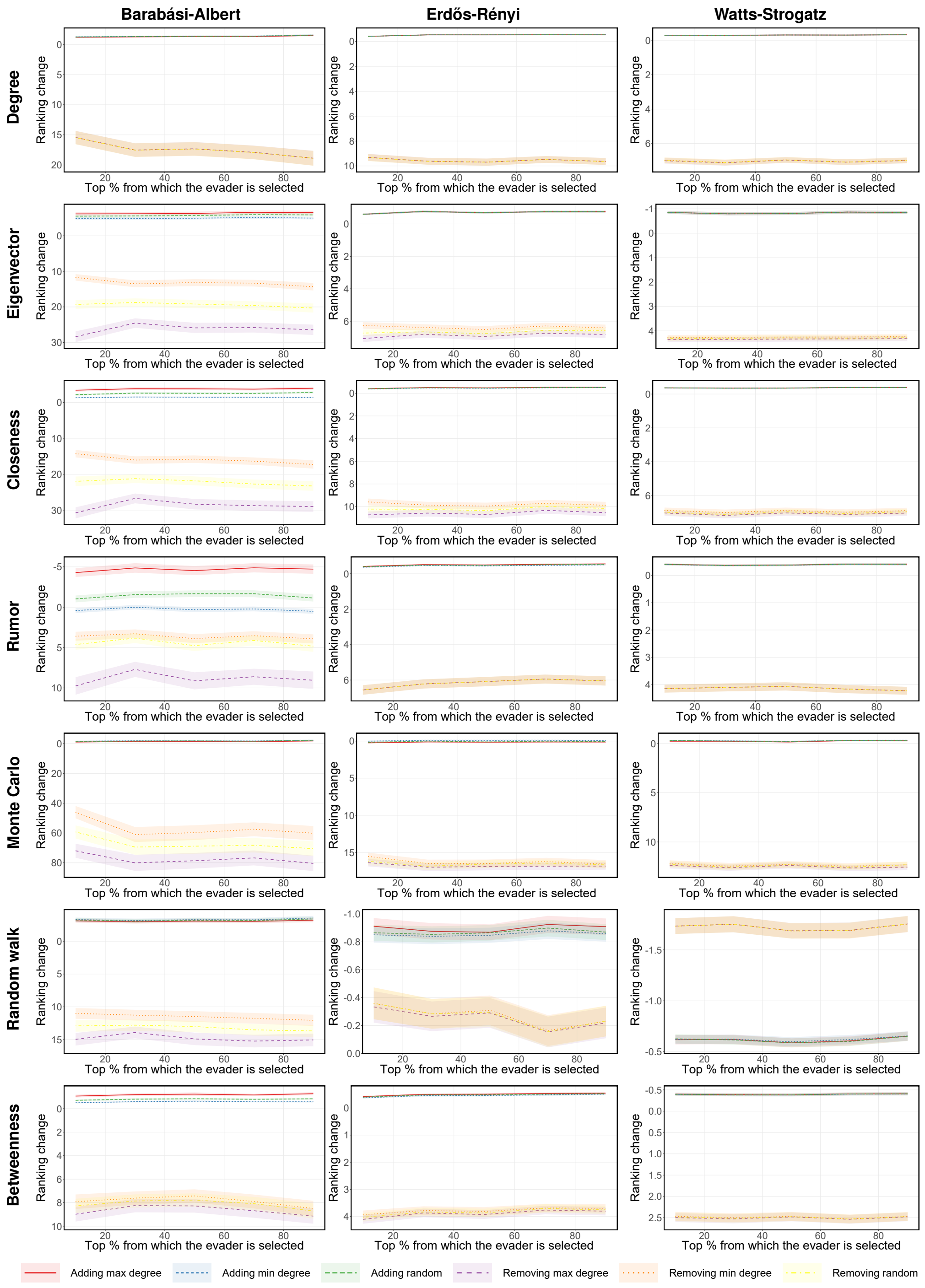

Figure S54: **Impact of the way of selecting the evader on the effectiveness of the evader's hiding for heuristics that modify edges, related to STAR Methods.** The x-axis corresponds to the percentage of top-ranked nodes (according to degree) from which the evader is randomly selected (e.g., $x = 30\%$ corresponds to the case when the evader is selected randomly from the 30% with the highest degrees). The y-axis represents the change in the evader's ranking after the hiding process (greater value indicates more effective hiding). Shaded areas represent 95% confidence intervals.

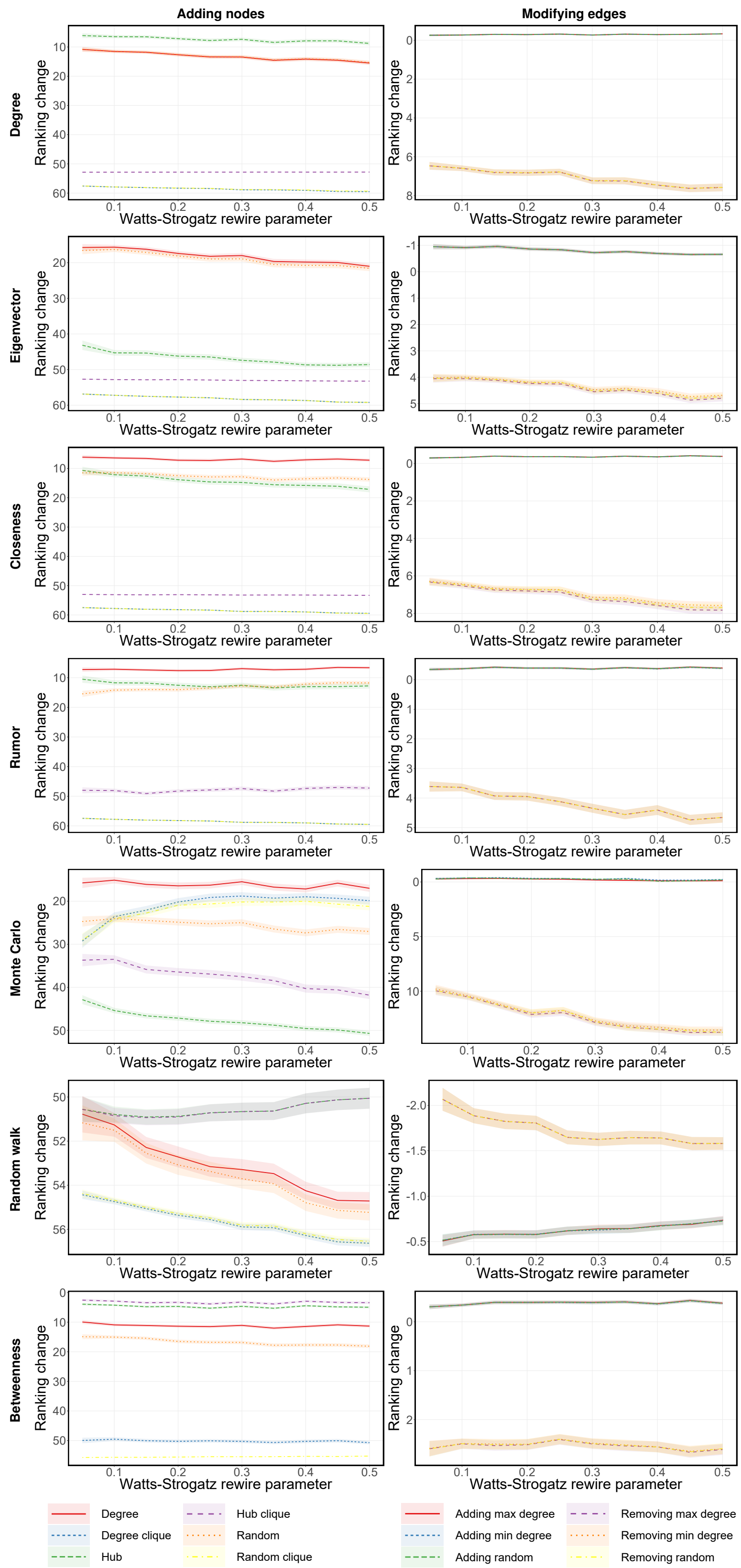

Figure S55: **The impact of the rewiring parameter of the Watts-Strogatz model on the effectiveness of the evader's hiding, related to STAR Methods.** The x-axis corresponds to different values of the parameter. The y-axis represents the change in the evader's ranking after the hiding process (greater value indicates more effective hiding). Shaded areas represent 95% confidence intervals.

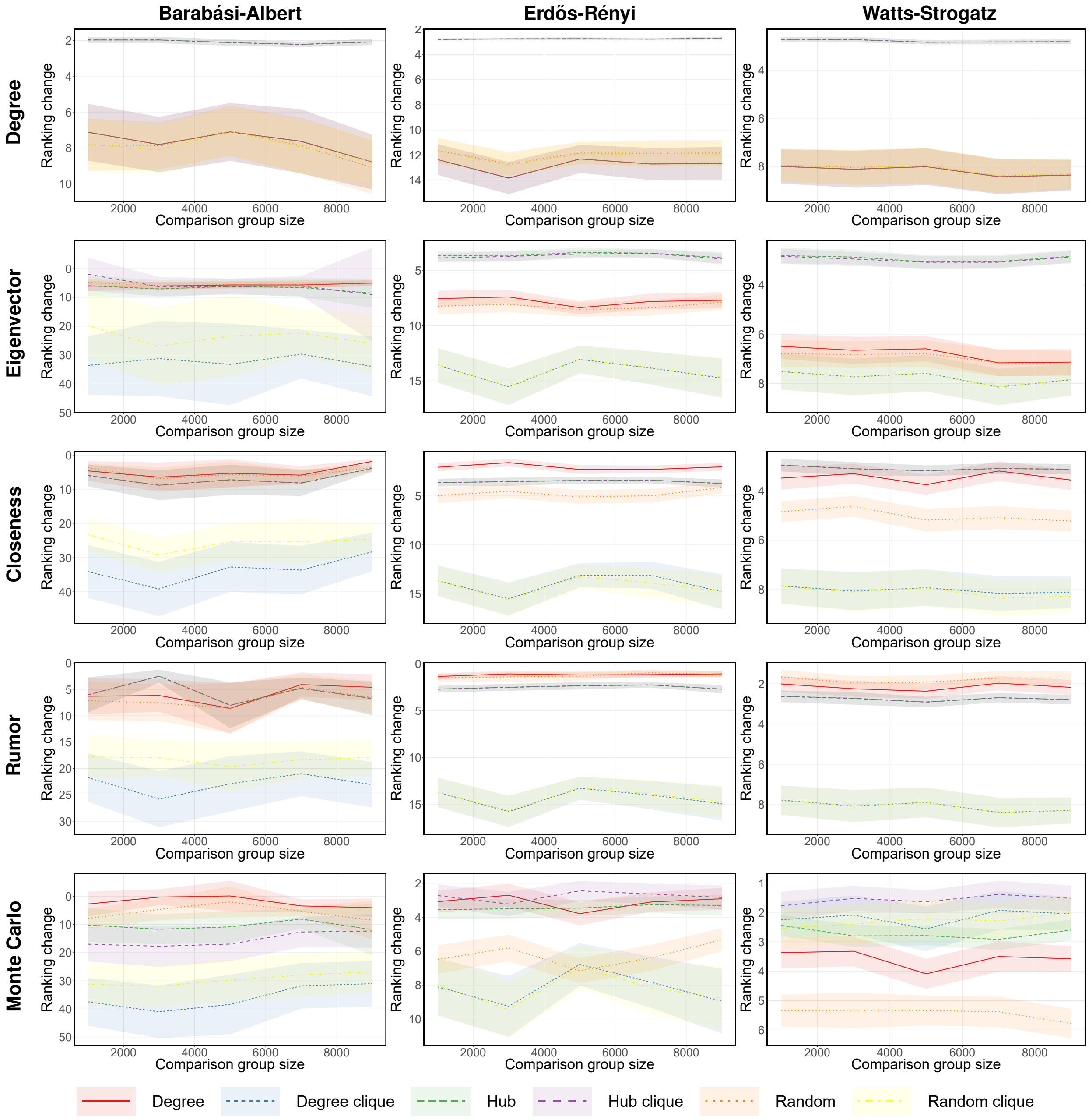

Figure S56: **The impact of the size of the comparison group on the experiments with large networks for heuristics that add nodes, related to STAR Methods.** The x-axis corresponds to the size of the comparison group. The y-axis represents the change in the evader's ranking according to different source detection algorithms after the hiding process (greater value indicates more effective hiding). Shaded areas represent 95% confidence intervals.

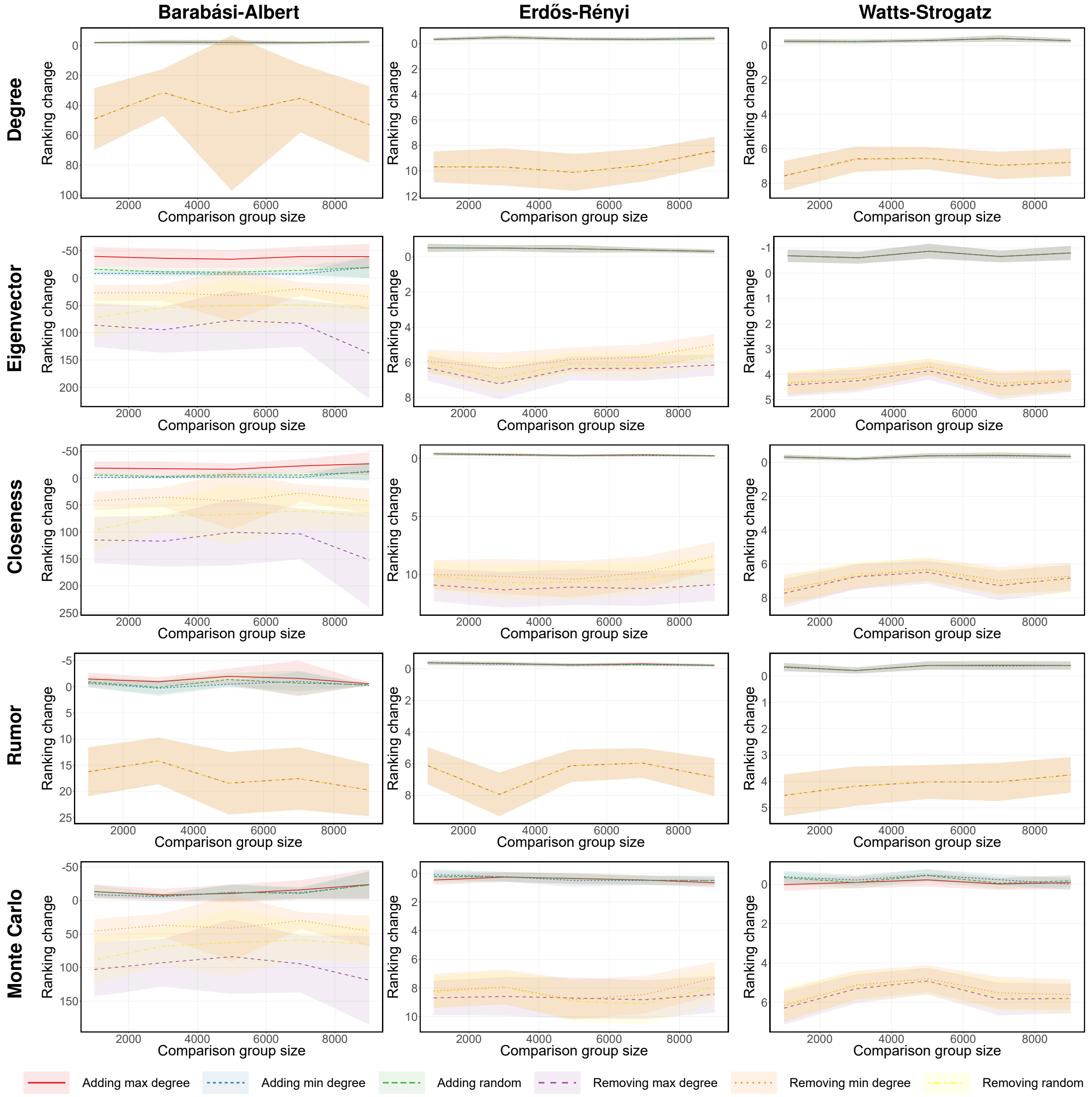

Figure S57: **The impact of the size of the comparison group on the experiments with large networks for heuristics that modify edges, related to STAR Methods.** The x-axis corresponds to the size of the comparison group. The y-axis represents the change in the evader's ranking according to different source detection algorithms after the hiding process (greater value indicates more effective hiding). Shaded areas represent 95% confidence intervals.
